# Systematic Review of Important Viral Diseases in Africa in Light of the ‘One Health’ Concept

**DOI:** 10.3390/pathogens9040301

**Published:** 2020-04-20

**Authors:** Ravendra P. Chauhan, Zelalem G. Dessie, Ayman Noreddin, Mohamed E. El Zowalaty

**Affiliations:** 1 School of Laboratory Medicine and Medical Sciences, College of Health Sciences, University of KwaZulu-Natal, Durban 4001, South Africa; ravendrachauhan@hotmail.com; 2 School of Mathematics, Statistics and Computer Science, University of KwaZulu-Natal, Durban 4001, South Africa; zelalem_getahune@yahoo.com; 3 Department of Statistics, College of Science, Bahir Dar University, Bahir Dar 6000, Ethiopia; 4 Infectious Diseases and Anti-Infective Therapy Research Group, Sharjah Medical Research Institute and College of Pharmacy, University of Sharjah, Sharjah 27272, UAE; anoreddin@sharjah.ac.ae; 5 Department of Medicine, School of Medicine, University of California, Irvine, CA 92868, USA; 6 Zoonosis Science Center, Department of Medical Biochemistry and Microbiology, Uppsala University, SE 75185 Uppsala, Sweden; 7 Division of Virology, Department of Infectious Diseases and St. Jude Center of Excellence for Influenza Research and Surveillance (CEIRS), St Jude Children Research Hospital, Memphis, TN 38105, USA

**Keywords:** Africa, emerging, re-emerging, infectious diseases, pandemic, SARS-CoV-2, COVID-19, virus, zoonosis, vector-borne, avian influenza, influenza A virus, coronaviruses, monkeypox, simian immunodeficiency, rabies, dengue, hemorrhagic fever, Rift Valley fever virus, West Nile virus, Ebola, one health, epidemiology

## Abstract

Emerging and re-emerging viral diseases are of great public health concern. The recent emergence of Severe Acute Respiratory Syndrome (SARS) related coronavirus (SARS-CoV-2) in December 2019 in China, which causes COVID-19 disease in humans, and its current spread to several countries, leading to the first pandemic in history to be caused by a coronavirus, highlights the significance of zoonotic viral diseases. Rift Valley fever, rabies, West Nile, chikungunya, dengue, yellow fever, Crimean-Congo hemorrhagic fever, Ebola, and influenza viruses among many other viruses have been reported from different African countries. The paucity of information, lack of knowledge, limited resources, and climate change, coupled with cultural traditions make the African continent a hotspot for vector-borne and zoonotic viral diseases, which may spread globally. Currently, there is no information available on the status of virus diseases in Africa. This systematic review highlights the available information about viral diseases, including zoonotic and vector-borne diseases, reported in Africa. The findings will help us understand the trend of emerging and re-emerging virus diseases within the African continent. The findings recommend active surveillance of viral diseases and strict implementation of One Health measures in Africa to improve human public health and reduce the possibility of potential pandemics due to zoonotic viruses.

## 1. Introduction

Africa is a large continent comprising 54 countries including some of the island nations within its geography. Various vector-borne and zoonotic virus diseases were reported from several African countries. Africa has a tropical climate, which enforces great diversity in its flora and fauna across the continent. The tropical climate, scarcity of resources, rampant poverty, and lack of knowledge coupled with cultural and traditional rituals and practices put many countries in the African continent at the edge of virus disease outbreaks. The high density of forest area and the tribes living within these forests mainly in west Africa, where human–animal conflicts are frequently encountered, put the communities at risk of disease progression and dissemination. One of the most challenging viruses, the human immunodeficiency virus (HIV), was reported to be transmitted to hunter gatherers in the forests of west Africa after conflicts with non-human primates during hunting for bushmeat. The Pygmy and Bantu tribes living within the forests in Western Africa have witnessed rampant infections of HIV [1].

The Ebola virus disease outbreak remains a global challenge and has recently been reported from several west African countries. An Ebola outbreak in non-human primates (chimpanzee and gorilla) as well as in the human population was reported from west Africa between 1994 and 2002 [2]. During the current Ebola outbreak in the Democratic Republic of Congo, as of 4 March 2020, the World Health Organization (WHO) has identified a total of 3444 Ebola cases, including 3310 confirmed cases and 134 probable cases. A staggeringly high mortality rate of 65.74% was observed in Ebola cases, with 2264 deaths reported so far [3]. A total of 1169 survivors are still under active care in the Democratic Republic of Congo [3]. The incidence of animal–human conflict and the close proximity to wild animals in the African wilderness were thought to be the primary factors behind the disease progression; however, human-to-human contact is another crucial factor for further disease dissemination [2].

Monkeypox is another zoonotic viral disease with a high prevalence in west Africa. The dependence of the local population on bushmeat is one of the major driving factors behind the spread of monkeypox in west Africa. Apart from this, exposure to the body fluids of infected individuals is another mode of human-to-human transmission of the disease [4].

Other parts of Africa have also reported different zoonotic virus diseases, including Rift Valley fever (RVFV), Crimean-Congo hemorrhagic fever (CCHFV), West Nile virus (WNV) disease, avian influenza, and rabies among several other viral diseases [5,6,7,8,9,10]. Countries all over Africa have reported different avian diseases affecting domestic and wild birds. Avian influenza is one of the most widely distributed avian viral diseases, which causes great economic losses to the poultry industry and an incipient threat to humans in the entire of Africa. The involvement of wild birds and waterfowls in the spread of avian influenza is well proven [11]. Various species of waterfowls though notably those belonging to the orders of *Anseriformes* and *Charadriiformes* have been reported to be the reservoir of avian influenza viruses [12]. IAV infections in wild birds and poultry have been reported from several African countries, including South Africa [5,13,14,15]. A recent incidence of highly pathogenic avian influenza virus (HPAIV) in an ostrich farm located in Western Cape Province of South Africa almost decimated the ostrich industry in South Africa [16]. Migratory wild birds have been reported to be responsible for the long-distance dissemination of highly-pathogenic avian influenza virus (HPAIV) subtype H5N1 [17]. Long-distance migration of wild birds is an important factor in the spread of avian influenza across the African continent [18]. Migratory waterfowls from European countries overwinter in the Rift Valley of Kenya, which is known as one of the favorite destinations of migratory birds for over-wintering [19]. The identification of a novel avian influenza virus H4N6 subtype from Kenya suggested that migratory water birds could act as a potential source of avian influenza transmission given that there was no earlier report of H4N6 subtype from the African continent [19]. Since influenza virus strains can cross the species barrier and therefore may emerge as new strains and recombination, they have a broader host range. It is suggested that the segmented nature of the influenza A virus genome may facilitate its evolution through re-assortment and mutation, and through these mechanisms, viruses would switch between hosts or find a new host and adapt or evolve in the new host [20,21].

Apart from poultry, swine farming is another large-scale industry in the African continent. The challenging aspect of swine farming is that swine are known to be a ‘mixing vessel’ for several viruses, including influenza viruses, which are reported to cause disease outbreaks in swine as well as in humans [20,21,22,23]. The evolutionary history of swine influenza viruses has been thoroughly investigated and reflects multiple introductions of these viruses into swine populations from other species [24,25]. Pigs are reported to be susceptible to influenza virus infection with both human as well as avian strains of the virus, and most interestingly, have been reported to be an important host for virus ecology and interspecies transmission of the virus [26,27,28]. The 2009 swine influenza H1N1 virus pandemic is thought to have originated from the avian strain, which was introduced into swine and further transmitted to humans [20,23]. There are various reports available on the incidence of influenza viruses worldwide, but only limited information is available from the African continent. Influenza A virus has recently been reported from pigs in Kenya [29]. Interestingly, pigs have recently been reported to be infected with the HPAIV H5N1 subtype in Nigeria [30] and with the 2009 pandemic H1N1 virus in Nigeria, Ghana [31], Cameroon [32], and Togo [33]. Interestingly, swine were found positive for the HPAI H5N1 subtype and low pathogenic avian influenza virus (LPAIV) H9N2 subtype in Egypt during 2014–2015 [34]. Currently, there is no information available on the prevalence of influenza viruses in pigs in South Africa.

Arboviruses, including yellow fever virus (YFV), dengue virus, and chikungunya virus infections have been reported from several African countries. These arboviruses are transmitted by mosquitoes and the abundance of mosquitoes in certain areas has resulted in a high incidence of these arboviruses [35]. West Nile virus (WNV) disease is a deadly zoonotic disease, which is thought to be transmitted by migratory birds into new regions [8]. Mosquitoes of the genus *Culex* are reported to be the main reservoir of WNV [36]. WNV has successfully been isolated from white storks, which are migratory birds [37]. Rift Valley fever (RVF) is another widely present disease in Africa, which largely affects ruminants [38]. Crimean-Congo hemorrhagic fever (CCHF) is a tick-borne disease, which develops hemorrhagic fever in the infected person with high fatality. CCHFV has been reported from several countries in the continent, including South Africa [39]. Apart from these, there are countless other significant virus zoonotic diseases that are in circulation in the African continent. Given the potential of virus evolution through reassortment and host switching, monitoring and continuous active surveillance of the virus diseases should be of utmost importance. Currently, there is no comprehensive information available on the status of vector-borne and zoonotic virus diseases in the African continent. Therefore, in this systematic review, virus diseases’ incidence and viral disease outbreaks in the African continent were reviewed, which provides insight into the current status of viral zoonotic diseases and possible risks of viral disease outbreaks in Africa. This is the first systematic review to be reported from Africa about viral diseases, including the most important zoonotic and vector-borne viral diseases in the continent.

## 2. Methods

### 2.1. Systematic Review Protocols

**Figure 1:**
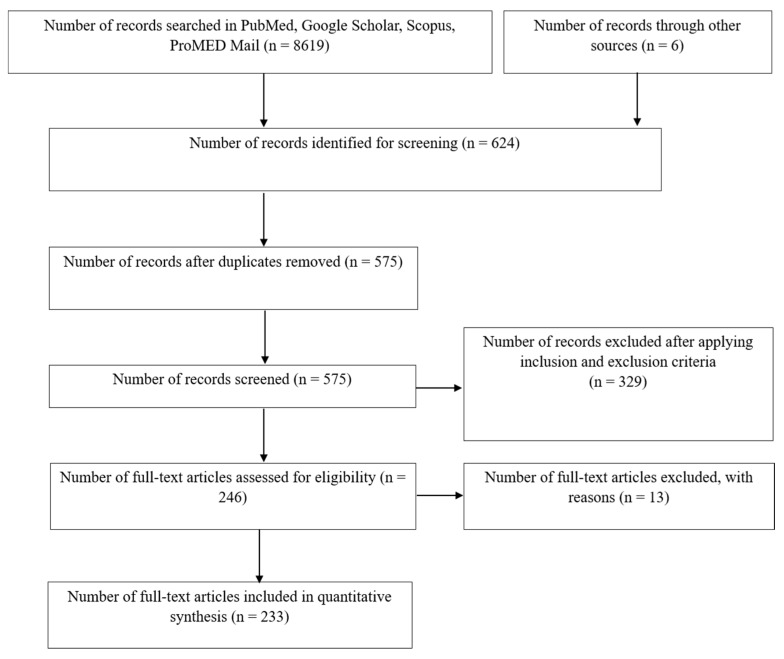
Preferred Reporting Items for Systematic Reviews and Meta-Analysis (PRISMA) flowchart illustrates the search strategy and selection process of the articles published until 25 September 2019 that were used in the present study. Based on the search criteria, a total of 8625 articles were identified, which were further refined as described in the PRISMA flowchart. Therefore, finally, 233 English language full-text articles were used for this systematic review.

The guidelines and procedures of the Preferred Reporting Items for Systematic Reviews and Meta-Analysis (PRISMA) [40] were followed in the current study (Figure 1).

### 2.2. Search Strategy and Eligibility Criteria

Original research and review articles that reported virus zoonotic and vector-borne diseases in humans and other species in localities within the African continent were searched for any available records published until 25 September 2019. Only original research, natural case reports, or review articles were included in this systematic review. Experimental studies that did not report natural cases were excluded from this study. The articles were primarily searched through four databases, including National Library of Medicine, National Center for Biotechnology Information (NLM-NCBI)-PubMed, Google Scholar, the Program for Monitoring Emerging Diseases (ProMED), and SCOPUS. The reported virus diseases in humans, livestock, wild birds, wild animals, pets, poultry, and slaughterhouses were screened. Initially, the databases were searched using variation of the search terms (“Zoonotic virus diseases in Africa” OR “Virus zoonoses in Africa”). Later, the search terms were further extended to search through the databases with individual country names for all the 54 countries that are located either within mainland African continent or its island nations. Therefore, the search terms were extended to, e.g., “Virus zoonosis in South Africa” OR “Zoonotic virus diseases in South Africa”, as well as “Virus zoonotic diseases in Zimbabwe”, “Virus zoonotic diseases in Madagascar”, “Virus zoonotic diseases in Mozambique”, and so on for all 54 African countries, including the island nations of Mauritius, Seychelles, Comoros island including Mayotte and Anjouan, as well as Cape Verde, Sao Tome and Principe, and La Reunion islands for the zoonotic virus diseases that were reported up until 25 September 2019. Full-length research or review articles were collected for this systematic review. Articles reported from outside Africa or those articles that did not report virus zoonosis within the African continent were not included for the drafting this systematic review. Additionally, editorials, conference proceedings, and articles in a language other than English were excluded from this systematic review. The article titles that reported a virus or zoonotic virus disease(s) in humans, domestic or wild animals, and birds were downloaded and stored for further refinement to be included in this study.

### 2.3. Data Screening

A database search was conducted, and the apparently relevant full text articles were accessed. The inclusion criteria were applied as follows:

- Only those articles reporting vector-borne and zoonotic viral diseases within Africa.
- The abstract of the stored articles were first read through to find out their relevance to be included, and, if necessary, the introduction and/or results and discussion sections of each article were thoroughly investigated to ensure that the articles met the inclusion criteria.

The articles thus selected were used as the background of the current study to discuss the vector-borne and zoonotic virus diseases reported throughout the African continent.

### 2.4. Statistical Analysis

Data were entered in a Microsoft Excel database (Microsoft, Redmond, WA, USA). The data were analyzed using the Statistical Package for Social Sciences (SPSS), version 25. Descriptive statistics, such as bar charts, were used to summarize the distribution of reported virus diseases by study year, region, host, and country.

## 3. Results and Discussion

**Figure 2:**
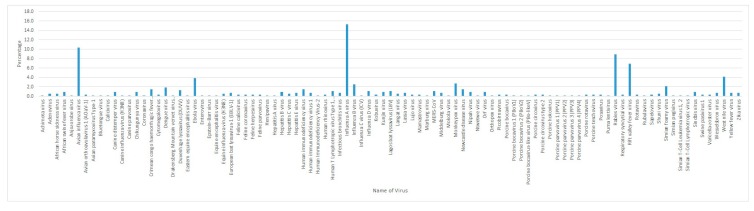
The overall frequency distribution of viral diseases in Africa in selected publications until September 2019.

Africa is a large continent comprising 54 mainland and island nations within the continent’s geographical area. The continent represents great diversity in its fauna and flora. Several vector-borne and zoonotic diseases of virus etiology have been reported from countries across the African continent in the past and to date (Figure 2 and Supplementary Table S1).

**Figure 3:**
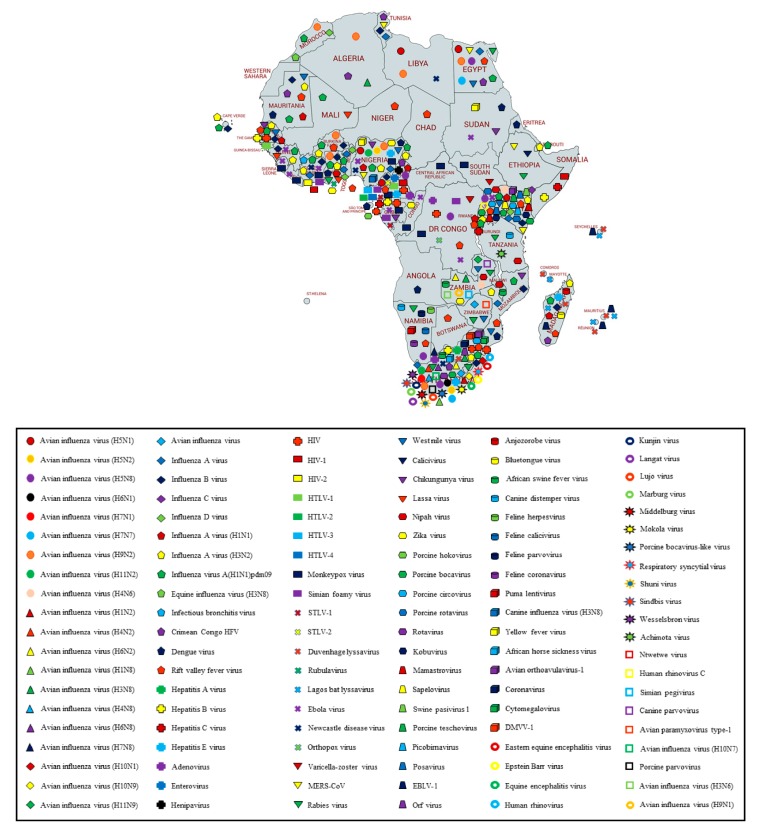
Map of Africa showing vector-borne and zoonotic virus diseases in selected scientific literature from Africa until September 2019.

Most of the reviewed vector-borne and zoonotic virus diseases were noticed to be reported mostly from Western, Eastern, and Southern African countries as compared to Northern African countries as shown in Figure 3.

The current systematic review illustrates a comprehensive analysis of all reported vector-borne and zoonotic viral diseases from all the countries within mainland Africa and its island nations. The current study discusses the virus diseases reported across five geographical regions of the continent viz., East Africa, West Africa, Central Africa, North Africa, and Southern Africa.

### 3.1. East Africa

**Figure 4:**
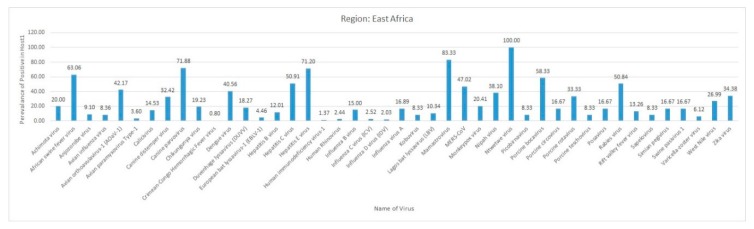
The overall frequency distribution of viral diseases in East Africa in selected publications until September 2019.

The eastern part of Africa comprises mainland countries, including Djibouti, Eritrea, Ethiopia, Kenya, Madagascar, Malawi, Mozambique, Somalia, Tanzania, Uganda, Zambia, and Zimbabwe, along with the island nations of Comoros, La Reunion, Mauritius, and Seychelles. The frequency distribution of the different reported virus diseases from African countries is shown in Figure 4.

#### 3.1.1. Comoros Island

Anjouan is part of the Comoros island nation in the Mozambique channel, located in the Indian Ocean between the southern African mainland and Madagascar in the eastern part of Africa. A study included 21 samples from different bat species, including blood from live bats species of *Miniopterus griveaudi* and *Chaerephon pusillus* and brain tissues from dead bats, to investigate the prevalence of lyssaviruses. These bats were primarily hunted for bushmeat purposes. Initially, the sera samples collected from bats were heated for 30 min at 56 °C to inactivate the complement and then lyssavirus-neutralizing antibodies were detected in the samples using a miniaturized rapid fluorescent focus inhibition test (RFFIT). Additionally, real-time RT-PCR was conducted to detect lyssavirus RNA in the bat samples using specific oligonucleotide primers targeting a conserved sequence of nucleoprotein gene of lyssaviruses under investigation, including Lagos bat lyssavirus (LBV), Duvenhage lyssavirus (DUVV), European bat lyssavirus-1 (EBLV-1), as well as rabies lyssavirus (RABV). In this study, only two bat sera samples were positive for LBV seroprevalence while two other sera were positive for DUVV prevalence out of eight samples that were tested. None of the samples were positive for lyssavirus RNA by real-time RT-PCR [41]. Many other viruses (Ebola, coronavirus, rabies, etc.) were reported to be transmitted due to exposure to or consumption of wild animals. The eating of or exposure to wild animals always puts humans at risk of zoonotic virus disease transmission.

Mayotte is one of the French islands and is a part of the Comoros island nation. A lyssavirus seroprevalence study was conducted in this region, targeting insectivorous and frugivorous bats between 2010 and 2015. Twenty-two sera samples were collected from the bats in Mayotte and samples were processed for lyssavirus diagnostics. Initially, sera samples were heated for 30 min at 56 °C to inactivate the complement and then lyssavirus-neutralizing antibodies were detected in the samples using the miniaturized RFFIT method. Additionally, real-time RT-PCR was conducted to detect lyssavirus RNA in the bat samples. As a result, two positive sera samples were observed for both LBV and DUVV antibodies in this investigation. The RNA samples were also screened for lyssavirus prevalence, but all were negative with real-time RT-PCR. Past exposure of bats to the lyssavirus was identified, hence any human–bat interaction may put humans at risk of disease transmission [41].

#### 3.1.2. Djibouti

After an influenza-like illness outbreak occurred at a US military base in Djibouti, nasopharyngeal swabs and nasal wash samples were collected from 32 symptomatic individuals of the active US troops and contractors during March–August 2009. Influenza viral RNA was identified in 27 samples: 25 were positive for H3N2 virus while two samples were positive for A(H1N1)pdm09 virus. Phylogenetic analysis showed that the hemagglutinin (HA) and neuraminidase (NA) genes of H3N2 viruses were closely related to the H3N2 sequences reported from the USA, Australia, and South-East Asia during the 2009 pandemic [42]. This finding suggested that the movement of the US troops or contractors would have introduced the viruses into the military camp.

#### 3.1.3. Eritrea

There were few outbreaks of dengue fever in Eritrea during 2005–2015. A study reported the prevalence of dengue fever virus and dengue fever outbreak in Eritrea during a 10-year period from 2005–2015. This was the first comprehensive study to provide information on the status of dengue fever from Eritrea [43]. Particularly, in this study, dengue fever cases having clinical symptoms reported to the hospitals were screened for the investigation. Additionally, sera from symptomatic clinical cases were collected for serological and virological investigations. Fifteen sera samples from patients having high fever, headache, joint or muscular pain, and anorexia, which are typical dengue fever symptoms, were collected from Agordet district, where the first outbreak was reported in 2005. Additionally, 26 sera samples were collected from clinical patients in Massawa district, where the second outbreak was reported in 2010. Serological investigation showed that five samples collected from Agordet district and 23 samples collected from Massawa district were positive for dengue virus antibodies. This was the first study to report the seroprevalence of dengue virus in Eritrea over a 10-year period [43].

#### 3.1.4. Ethiopia

Ethiopia is one of the world’s most affected countries for rabies, where 2771 people died during 2009–2010 because of rabies [44]. The study identified 55 rabies cases, including 32 humans and 23 animals exhibiting rabies-like symptoms, e.g., encephalitis, change in temper, vocalization, paralysis, and other visible neurological signs. Sixteen of these cases (3 humans and 13 animals) were bitten by dogs and died because of the disease severity [45]. Incidents of dog bites or contact with the saliva of suspected rabid dogs were reported among the human and animal cases, which suggested the zoonotic transmission of the disease from dogs to humans and other animals [45]. This finding was consistent with an earlier study, which reported that about 95% of rabies cases originated after dog bites [46].

A high seroprevalence of Middle East respiratory syndrome coronavirus (MERS-CoV) was reported in dromedary camels in Ethiopia during 2011–2013. Sera samples from 188 dromedary camels were collected across three provinces viz., Afar, Somalia, and Oromia. Serological investigation based on immunoglobulin G (IgG) antibodies detected MERS-CoV in 93% and 97% of the juveniles and adults, respectively. A total of 175 sera samples were found positive for MERS-CoV. The high genetic similarity of MERS-CoV isolates retrieved from humans and dromedary camels suggested the zoonotic transmission of the disease [47].

In total, 117 fecal samples were collected from either diarrheic or apparently healthy pigs with no other clinical signs of illness in Bishoftu, Ethiopia between June and September 2013. RT-PCR diagnostics detected 17 swine samples that were positive for caliciviruses. The PCR amplicons were purified on agarose gel and four of the RT-PCR-positive samples were sequenced using Sanger sequencing. Sequence analysis identified two human norovirus genomes as well as two porcine sapovirus sequences. The occurrence of human norovirus sequences in domestic pigs suggested the transmission events of noroviruses from humans to swine in Ethiopia. This represents a reverse zoonotic disease transmission (zooanthroponosis) and was the first investigation of the prevalence of enteric caliciviruses in Ethiopian domestic swine [48].

#### 3.1.5. Kenya

A surveillance study including migratory waterfowls, gulls, pelicans, and storks was conducted to investigate the prevalence of avian influenza A virus in these European migratory wild birds, which overwinter in the Rift Valley of Kenya [19]. Fecal swabs were collected from 2630 individual birds and pooled into 516 samples (3 to 5 samples per pool) for testing with real-time RT-PCR. A total of 12 pools (2.3%) were found positive for the matrix gene sequence of avian influenza virus. However, none of the pools were positive for the H5 or H7 subtypes, but, interestingly, two pools were positive for the H4N6 virus. This is an interesting observation because there was no earlier report of H4N6 virus from the African continent [19]. This study suggested that migratory water birds could act as a carrier for avian influenza A viruses.

During 1991–2015, a surveillance study was conducted to investigate the prevalence of influenza C virus (ICV) and influenza D virus (IDV) in cattle and camel populations in Kenya. A total of 931 sera samples from cattle and 293 sera samples from camels were collected, which revealed a 10.6% seroprevalence for ICV and 8.2% seroprevalence for IDV in camels [49]. Cattle samples were negative for both ICV and IDV. This finding revealed that the camel was a new host for ICV, but the source of ICV infection in camels could not be determined. However, the import of ruminants infected with IDV was suspected as a possible cause of IDV infection among camels [49].

Immunohistochemistry was used to determine RVFV infections in liver tissue samples obtained from six animal carcasses and 11 human corpses in Kenya [50]. The majority of these tissue samples exhibited extensive hepatocellular necrosis, suggesting severe RVFV infection [50].

A metagenomic study identified several viruses in pig fecal samples collected from 12 different smallholder swine farms located in Kenya and Uganda. Total viral RNA was extracted from the samples, which was subjected to Illumina sequencing on an MiSeq platform. Sequence analyses identified porcine circovirus, rotavirus, bocavirus, sapelovirus, mamastrovirus, posavirus, picobirnavirus, swine pasivirus 1, porcine teschovirus, and kobuvirus in swine fecal samples [51].

Many human cases of IAV and IBV infections were reported during 1999–2014. In total, 365 cases of H1N1, 1285 cases of A(H1N1)pdm09, 1183 cases of H3N2, and 1454 cases of IBV infections were reported [52], which reflected the circulation of influenza viruses. Another study in Kenya during 2005–2015 identified 140 poultry samples that were positive for Newcastle disease virus, which is now termed avian orthoavulavirus-1 (AOaV-1) [53].

A study was conducted to monitor the prevalence of IAV in different household animals in Kenya during 2010–2012. Overall, 1491 swine swab, 3863 chicken swab, 172 swine sera, and 1894 chicken sera samples were collected. Additionally, sera and swabs were also collected from other domestic animals and birds, including dogs, cats, ducks, and turkeys. Serology using ELISA using specific anti-IAV antibodies identified one cat, two chickens, three dogs, three ducks, and 13 pigs that were seropositive for IAV antibodies. Real-time RT-PCR for the matrix gene of IAV detected 24 chickens, four dogs, five ducks, and 11 pigs that were positive for the active infection. Subtyping using real-time RT-PCR for the HA and NA genes identified that eight pigs were infected with the A(H1N1)pdm09 virus. Phylogenetic analysis revealed that the A(H1N1)pdm09 virus identified in pigs was closely related to the A(H1N1)pdm09 virus, which has been in circulation in the human population in Kenya since 2009. Therefore, this investigation suggested a reverse zoonotic transmission of the A(H1N1)pdm 09 virus from humans to pigs in the country [29].

**Figure 5:**
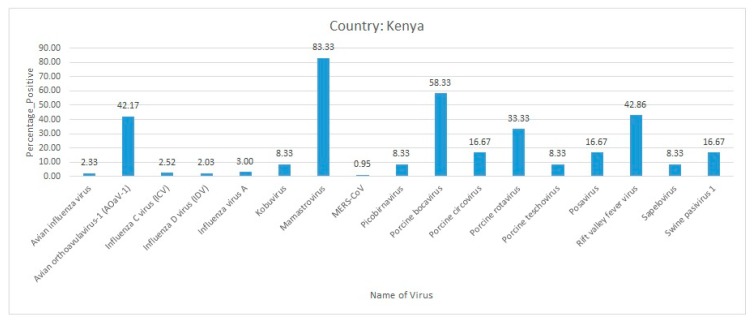
Frequency distribution of viral diseases in Kenya in selected publications.

During January–June 2018, 1163 plasma and nasal swab samples were collected from dromedary camels across 13 counties in Kenya. Most of the samples were collected from counties located in northern Kenya bordering with the Republic of Somalia. ELISA followed by a microneutralization (MN) assay using Vero B4 non-human primate cell lines identified 792 plasma samples that were positive for the MERS-CoV antibodies. Active infection of MERS-CoV was detected in only 11 nasal swab samples. Using Vero cells, two MERS-CoV isolates were successfully retrieved for whole-genome sequencing. Phylogenetic analysis found that these MERS-CoV sequences were distinct from the sequences reported from the Arabian Peninsula. This study was the first report of the MERS-CoV whole genome sequence from Kenya [54]. In general, avian orthoavulavirus-1, mamastrovirus, porcine bocavirus, and RVFV were reported with a high percent positivity in Kenya (Figure 5).

#### 3.1.6. La Reunion

La Reunion is officially a French island located in the Indian Ocean east of Madagascar and southwest of Mauritius. In this region, a study was conducted to determine the seroprevalence of lyssavirus in insectivorous and frugivorous bats (*Mormopterus francoismoutoui*) between 2010 and 2015. A total of 121 bat sera and tissue samples were collected and processed for lyssavirus diagnostics. Real-time RT-PCR was conducted to detect lyssavirus RNA in the bat samples using specific oligonucleotide primers targeting a conserved sequence of the nucleoprotein gene of lyssaviruses. Three bats were found seropositive for LBV while 14 sera samples were positive for DUVV and nine samples were positive for EBLV-1. Interestingly, all RNA samples were negative with real-time RT-PCR for lyssavirus [41], which suggested a past exposure to these viruses but no active infection.

#### 3.1.7. Madagascar

Anjozorobe virus is a representative virus of Thailand orthohantavirus (THAIV) in the family *Bunyaviridae*, which was named because of its discovery from Anjozorobe-Angavo forest in Madagascar. A surveillance was conducted to find out the prevalence of this virus in rodent species in Madagascar. A total of 1242 samples were collected from 7 representative species of rodents found in Madagascar. A total of 111/897 samples (12.4%) of *Rattus rattus* species and 2 out of 125 (1.6%) samples of *Mus musculus* species of rodents were found to be positive with nested PCR for the infection of Anjozorobe virus [55]. The study suggested a high zoonotic transmission risk to the human population living in households given the prevalence of Anjozorobe virus in household-dwelling rodent species [55].

Another country-wide serological surveillance was conducted across 106 administrative districts in Madagascar to investigate the seroprevalence of CCHFV. In this investigation, 1995 human participants working in slaughterhouses since at least 2007 were enrolled. This was considered a group of high-risk individuals given the nature of their occupation. The human sera samples were tested against CCHFV-specific immunoglobulin G- (IgG) and M (IgM) antibodies. As a result, only one human subject was identified with a recent CCHFV infection while 15 workers appeared to have a past exposure of the disease [7].

Bluetongue virus (BTV) is a member of the *Reoviridae* family and is reported to be transmitted by certain biting species of midges and mosquitoes [56]. In a surveillance to assess the prevalence of BTV in Madagascar, 4493 sera samples from cattle and small ruminants, including goat and sheep, as well as 12,785 adult mosquitoes were collected during August 2008 and April 2009. Mosquitoes were divided into 390 pools, grinded, and the supernatant was used for viral analysis. Virus isolation from the supernatant of mosquito pools was carried out using mosquito AP61 cell lines. Further, the supernatant from mosquito pools were tested for virus identification using an indirect immunofluorescence assay, which revealed that one of the pools of *Anopheles squamosus* mosquitoes was positive for BTV infection. Interestingly, 136 cattle had antibodies against BTV while 39 samples were seronegative. This was the first report of BTV circulation from Madagascar [57]. Bluetongue disease is an arthropod-vectored virus; hence it can be easily transmitted among livestock and wild ruminants.

Human cases of IAV and IBV infections have also been reported in Madagascar. A surveillance during 1999–2014 identified 109 H1N1, 1101 A(H1N1)pdm09, and 579 H3N2 subtype cases of IAV and 1004 cases of IBV infections [52]. Another investigation reported a seroprevalence of RVFV during May-June 2009 in Anjozorobe district. Several factors, including the proximity to the water point, forest, etc., were taken into consideration during sampling to monitor the risk factors for disease outbreak. The anti-IgM ELISA test detected seven cattle having RVFV antibodies. Five of these infected cattle appeared to have local infections. The study suggested that the close proximity of the cattle to the forest or water bodies might have played an important role in the disease dissemination as the *Culex* and *Aedes* mosquitoes may serve as a vector for RVFV [58].

In total, 301 samples including blood from live bats and brain tissues of dead bats from different bat species i.e., *Hipposideros commersoni*, *Miniopterus* species, *Chaerephon* species, *Mops* species, *Mormopterus jugularis*, *Otomops madagascariensis*, *Eidolon dupreanum*, *Pteropus rufus*, *Rousettus madagascariensis*, *Triaenops menamena*, and *Myotis goudoti* were investigated for the prevalence of lyssaviruses. These bats were hunted for bush meat purposes between 2010 and 2015. The lyssavirus-neutralizing antibodies were detected in the samples using miniaturized RFFIT. As a result, 23 samples were positive for LBV, 54 sera were positive for DUVV, and only one serum was positive for EBLV-1. None of the samples were positive for lyssavirus RNA through real-time RT-PCR. Since bats are a reservoir for several zoonotic viruses, the trade of bats for bushmeat in the region puts the human population at risk of zoonotic virus transmission [41].

A retrospective study identified 60 (14.1%) human sera samples having hepatitis E virus (HEV) antibodies. These sera samples were collected from slaughterhouse workers in 18 districts during September 2008 to May 2009. Additionally, sera and liver tissue samples were collected from 250 pigs between November 2010 and January 2011. Interestingly, 178 swine sera also had HEV antibodies. Then, total nucleic acid was extracted from the pig liver tissue samples and cDNA was synthesized, which was subjected to an HEV TaqMan qPCR assay for the detection of hepatitis E virus RNA. Positive amplicons were gel-purified for ligation into the pGEM-T Easy vector for sequencing. Out of 250 swine liver tissue samples, only 3 were positive for HEV RNA. This was the first serological as well as virological investigation confirming the past prevalence of HEV in human and swine populations in Madagascar [59].

#### 3.1.8. Malawi

Africa’s most common fruit bat (*Eidolon helvum*) is known as a reservoir of zoonotic virus diseases. Serological and genetic studies based on mitochondrial (cytochrome b) and nuclear DNA analyses reported that the panmictic continental population of *E. helvum* facilitates zoonotic transmission [60]. Urine, blood, and wing biopsy samples were collected from 22 bats. Antibodies specific to soluble G glycoproteins of Nipah virus (NiV) confirmed that four samples were serologically positive for Nipah virus antibodies [60]. Based on Bayesian analysis, no bats were identified as the recent migrant or the first-generation migrant to their population in the regions. Thus, this study concluded that the *E. helvum* population across the sample sites in the African continent, including sites in Malawi, represented panmictic continental populations. This finding raised the concern of a higher risk of transmission of zoonotic virus disease to human populations that may be exposed to the excreta or body fluids of *E. helvum* living in colonies near human settlements in the region [60].

A devastating outbreak of African swine fever virus (ASFV) disease occurred in domestic pigs during 1981–1984. As a result, a significant number of pigs were reported dead in the affected areas. ELISA and indirect immunofluorescence detected ASFV antibodies in 149 swine sera samples [61]. Additionally, 17,405 ticks (*Ornithodoros moubata*) were also collected from domestic pig kholas, houses, and a single warthog habitat across nine of the 24 districts in Malawi during 1982–1984. Ticks were pooled in different groups. A total of 181 pools of ticks collected from pig kholas were positive for ASFV while 48 pools collected from the houses in Mchinji district were positive [62]. This suggested a tick-borne transmission of ASFV in swine.

#### 3.1.9. Mauritius

Mauritius is an island nation in the Indian Ocean located approximately 1200 miles southeast of the African continent. In a lyssavirus seroprevalence study during 2010–2015, a total of 67 blood and tissue samples were collected from insectivorous and frugivorous bats of *Mormopterus acetabulosus* and *Pteropus niger*. This study identified seven LBV-, 19 DUVV-, and 2 EBLV-1-positive sera samples collected from different bat species within the island. On the contrary, real-time RT-PCR could not amplify lyssavirus sequences from the bat samples and hence failed to document an active infection [41].

#### 3.1.10. Mozambique

In a sero-surveillance conducted in Mozambique during 2012–2013, 78 sera samples collected from febrile patients living in Maputo city were screened for the chikungunya virus, dengue virus, RVFV, and WNV. Indirect immunofluorescence assays followed by ELISA found 15 sera with chikungunya virus and 10 with dengue virus antibodies. One serum had RVFV and three sera had WNV antibodies. This investigation revealed that the prevalence of vector-borne viruses is frequent among people living in the suburban areas of Maputo city in Mozambique, which suggested the need for active surveillance for these virus diseases in the region [63].

A study conducted at two different hospitals in Maputo city investigated the prevalence of influenza viruses. Nasopharyngeal and/or oropharyngeal swabs were collected from 1140 patients. Real-time RT-PCR identified 46 patients as positive for influenza virus active infection. Subtyping could be done for 20 of the 46 influenza-positive samples, which determined that 13 patients were positive for H3N2, 4 for A(H1N1)pdm09, and another 3 patients were infected with IBV. Phylogenetic analysis determined that the influenza viruses isolated in Mozambique were similar to the influenza viruses reported in Southern African regions [64]. This suggested a travel-related dissemination of the viruses.

#### 3.1.11. Seychelles

Seychelles is an archipelago nation located east of Africa in the Indian Ocean between the southern African mainland and Madagascar. A lyssavirus seroprevalence study was conducted in Seychelles in insectivorous and frugivorous bats of *Pteropus seychellensis* during 2010–2015. In total, 40 sera samples were collected from the bats and processed for the detection of lyssavirus-neutralizing antibodies using a miniaturized RFFIT test. As a result, six positive sera were detected for LBV and four for DUVV while only one sera sample was positive for EBLV-1. Additionally, real-time RT-PCR was conducted to detect lyssavirus RNA, which suggested no active infection. Although an active infection could not be identified, but since bats are the reservoirs of zoonotic viruses, the exposed human population is always at risk of contracting the disease [41].

#### 3.1.12. Somalia

The circulation of hepatitis B virus (HBV) was reported from three villages in Somalia. Practices of maintaining poor hygiene and cleanliness were reported in these villages. Additionally, the villages were over-crowded, and the residents were living under primitive housing conditions. The sera samples collected from 331 adults and 52 children were tested for the surface antigen of HBV-hepatitis B surface antigen (HBsAg), as well as anti-HBsAg, anti-hepatitis B core antigen (HBcAg), and anti- hepatitis B envelope antigen (HBeAg). A seroprevalence of 12.08% for HBsAg antibodies was observed in the samples, which reflected the circulation of HBV in Somalian villages [65].

In 1990, another study was conducted in three major urban areas of Somalia viz., Mogadishu, Chismayu, and Merca. In this study, 236 sera samples were collected from female prostitutes, 80 sera were collected from patients belonging to the sexually transmitted disease group, 79 sera belonged to male military personnel, and 43 sera were obtained from patients suffering from *Mycobacterium tuberculosis* disease. Overall, 438 sera samples were subjected to anti-HCV ELISA for the screening of hepatitis C virus (HCV) as well as for the seroprevalence of human immunodeficiency virus-1 (HIV-1). The repeatedly reactive sera for the HCV were further tested with the recombinant immunoblot assay (RIBA-2). Only those sera that were reactive to both assays were considered positive. On the contrary, reactive sera for HIV-1 were further tested with the Western blot assay and hence samples positive for both tests were considered positive. This study found that eight sera samples were reactive to both assays for HCV while only six sera samples were positive for HIV-1 ELISA and Western blot [66]. Therefore, only a very small proportion of the population was found to be infected with HCV and HIV-1 in this investigation. The results also suggested that there was a low risk of sexual transmission of HCV in Somalian villages at the time of this investigation [66].

#### 3.1.13. Tanzania

Bats are implicated to carry several novel emerging viruses of pandemic potential which can infect other animal species and humans when spillover occurs. A study was undertaken to assess the zoonotic potential of two novel paramyxoviruses named Achimota virus 1 (AchPV1) and Achimota virus 2 (AchPV2) in 25 sera samples collected from a roost of fruit bats (*Eidolon helvum*) near Dar es Salam. Sera samples were collected from 226 children in the age group of 2 months to 13 years having febrile illness and admitted to a hospital. Serology confirmed that only one human sample (0.4%) was positive for AchPV2. Interestingly, three (12%) bat samples were positive for AchPV1 and two (8%) were positive for AchPV2 [67]. This study reported the prevalence of novel rubulaviruses named AchPV1 and AchPV2 across bat populations in the region. The human population living in proximity to the roost of *E. helvum* would be at risk if they came in contact with the urine, tissues, or excreta of these bats [67].

Canine distemper virus (CDV) is a morbillivirus, which is primarily known to infect domestic dogs [68]. Serengeti National Park (SNP) is a protected wildlife reserve where dogs usually come in contact with wildlife species, including hyena, jackal, and lions, among others. The first known CDV outbreak in SNP killed 54 lions (*Panthera leo*) in the year 1994 in a population of 250 lions; the dead lions had neurological disease symptoms of seizures and pneumonia [69]. This study included 23 dead lions, 13 symptomatic, and 72 apparently healthy lions in the SNP. Additionally, sera samples from 111 healthy lions collected over a period of 10 years from 1984 to 1994 were also analyzed to assess the CDV seroprevalence. Approximately 85% of the SNP’s lion population was found serologically positive for anti-CDV antibodies [69]. CDV was successfully isolated from one lion. The investigation established that the virus in lions was closely associated with the virus reported from a dog found in the region, hence a transmission from dogs to lions was established, which not only affected the lions but other wildlife animals as well in the SNP [69].

A study was conducted to understand the transmission dynamics and persistence of zoonotic henipaviruses in *Eidolon helvum* bat populations. Serological and genetic studies based on mitochondrial (cytochrome b) and nuclear DNA analyses reported that the panmictic continental population of *E. helvum* would facilitate virus transmission across its colonies in the African continent, including Tanzania [60]. Urine, blood, and wing biopsy samples were collected from 263 bats in Tanzania. Nipah virus (NiV)-specific antibodies confirmed 117 seropositive samples. Microsatellite genotyping revealed high levels of allelic heterozygosity (0.75 ± 0.25) but low mitochondrial DNA diversity (0.011 ± 0.0011) among Tanzanian samples [60]. This investigation concluded that the *E. helvum* population across the sample sites in African continent, including sites in Tanzania, represented panmictic continental populations. This finding raised the concern of a higher risk of transmission of zoonotic virus disease to human populations that may be exposed to the excreta or body fluids of *E. helvum* [60].

During April–May 2018, sera samples were collected from 278 household dogs for the diagnostics of rabies virus. ELISA test was used to detect rabies virus antibodies in 94 samples. However, most of the households were aware of rabies but were not aware of its wide host range. The seroprevalence of rabies virus in dogs in households puts household members at risk of contracting the virus. This would explain the high death rate (1500 people per year) each year due to rabies virus infection in Tanzania [70].

#### 3.1.14. Uganda

There were two Ebola virus disease outbreaks reported from Uganda. The first outbreak was reported from Gulu in the year 2000 where 425 cases were reported with 224 deaths; Ebolavirus-Sudan (EBOV-S) was the etiological agent of this outbreak. The second outbreak was reported in November-December 2007 in Bundibugyo where 116 confirmed cases with 30 fatalities were reported; Ebolavirus-Bundibugyo (EBOV-B) was the etiological agent behind this outbreak [71]. In this study, the migration of bats was correlated with the outbreaks in African countries, including Uganda. The hunting and trade of bats for meat in these regions was considered a major risk factor for disease transmission. Additionally, human-to-human transmission can also not be ruled out [71]. Interestingly, a large Ebola outbreak erupted in Uganda in early 2019, but fortunately, it was quickly contained. The neighboring country, the Democratic Republic of Congo, is currently experiencing high mortality due to the Ebola virus disease [3].

Rhinovirus C has been reported from human populations in sub-Saharan Africa [72]. From February to September 2013, three different phases of a rhinovirus C outbreak among the chimpanzee population appeared in Uganda. During this outbreak, five chimpanzees (one infant and four adults) died in a population of 56, representing an 8.9% mortality rate [73]. The autopsy of a dead infant chimpanzee revealed that the morphology of lung parenchyma was affected, with hepatic congestion and hepatomegaly. It was observed that this infant chimpanzee died due to pneumonia. The deep sequencing analysis found the sequences of rhinovirus C, which was further confirmed using real time RT-PCR. Interestingly, it was observed that the Kibale National Park where the community of these chimpanzees is based is usually visited by a number of tourists, researchers, and other local people living nearby the reserve, hence the possibility of transmission of the disease from contact appears to be the most likely transmission pathway [73].

A study reported the transmission dynamics and persistence of zoonotic henipaviruses in *E. helvum* bat populations in Uganda [60]. Samples were collected from seven bats: NiV-specific antibodies were confirmed six seropositive samples. This finding raised the concern of a high risk of transmission of zoonotic virus disease to human populations that may be exposed to the excreta or body fluids of *E. helvum* [60].

A novel orthobunyavirus named Ntwetwe virus was reported from a three-year-old female child resident of Ntwetwe village in Uganda in February 2016. This child reported fever, abdominal pain, and headache, which worsened as days passed by. The patient went into a coma after two weeks of the onset of the disease. Diagnosis was carried out for the possible viral and bacterial diseases being circulated in the region but were negative [74]. Hence, cerebrospinal fluid (CSF) and plasma samples were used for viral metagenomic sequencing to investigate the possible etiological agent of the disease. However, the bulk of the sequence could not identify a probable virus, but two short reads exhibited some similarity with several orthobunyavirus genome L-segments [74]. Therefore, the technique of genome walking was used to further extend these reads up to 936 nucleotides. An orthobunyavirus known as Tataguine virus appeared to share some similarity with this 936-nucleotide sequence, but still there was significant genetic variation and thus, this sequence was considered of a novel orthobunyavirus, which was termed as Ntwetwe virus based on the name of the village of the patient. Ntwetwe virus was most likely referred to as an arbovirus vectored by *Anopheles* mosquitoes. There has not been any other case of Ntwetwe virus from this region; hence, the case of this girl child was a rare and unique event. The study suggested that mosquitoes or an animal reservoir might be the probable source of transmission of this virus disease [74].

WNV was first reported from the West Nile district of Uganda in the year 1937 [75]. WNV was first isolated from a Ugandan woman of about 37 years of age who was enrolled for a sleeping sickness study and reported a slightly elevated body temperature as the cause of concern but was never hospitalized [75]. The majority of the WNV cases have been reported to be sub-clinical and only a few showed symptoms. Headache, malaise, nausea, vomiting, arthralgia, and myalgia are among the most common symptoms of WNV disease in humans. A few cases have also been reported having polio-like symptoms with WNV infection [76]. WNV has been reported to be vectored by *Culex* mosquitoes. Certain avian species are considered as the reservoir host for WNV. Wide-scale transmission has usually been reported by migratory birds [76].

An outbreak of RVFV occurred in domestic animals in Kisoro district of Uganda in 2016, leading to frequent abortions in pregnant animals. A sero-surveillance was initiated, which included 338 cattle, 323 sheep, and 336 goats. Anti-IgG ELISA was conducted to assess the seroprevalence of RVFV antibodies in the collected samples. The results revealed that 70 cattle were seropositive for RVFV along with 22 sheep and 12 goats. The outcome of this investigation suspected the zoonotic spread of the disease in Kisoro district [9].

Patients visiting outpatient departments of different Ugandan hospitals during July 2009 through February 2010 and then July 2010 through April 2011 suffering from fever, sore throat, and cough were enrolled in a surveillance for influenza virus diagnostics. Samples were collected from the patients and screened for A(H1N1)pdm09 virus using the real-time RT-PCR assay. The positive samples were used for virus isolation; as a result, 199 virus isolates were retrieved. The genomes of virus isolates were also sequenced. Sequence alignment and phylogenetic analyses identified 73 A(H1N1)pdm09 virus isolates [77]. Since A(H1N1)pdm09 virus was the etiological agent of the 2009 influenza pandemic, the large number of virus isolates recovered in Ugandan people suggested a travel-related outbreak in the region.

**Figure 6:**
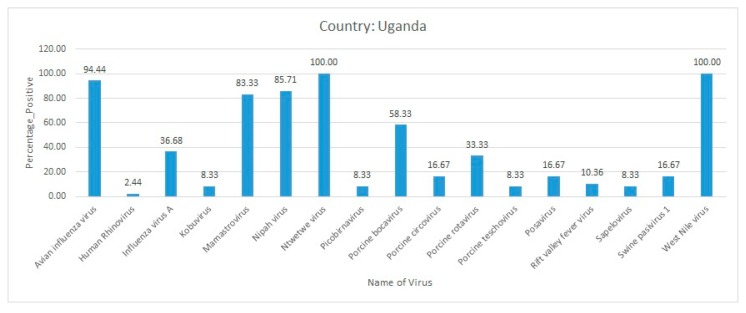
Frequency distribution of viral diseases in Uganda in selected publications.

In January 2017, high mortality was observed in white-winged black terns in Wakiso district of Uganda. Internal organs as well as oropharyngeal and cloacal swabs from dead birds were collected and investigated for IAV infections. Wild birds exhibited the clinical symptoms of torticollis, depression, lethargy, and convulsions just before death. Since the tern samples were positive for the IAV, subtyping for H5 virus was done, which identified few H5-positive tern samples. Immediately after this outbreak in terns, the disease was also observed in chickens and ducks in Masaka district. Eighteen clinical samples obtained from chickens and ducks were investigated. Molecular investigation followed by sequencing identified the H5N8 virus in 17 samples. Phylogenetic analysis identified a high similarity (99.5%) of Ugandan H5N8 virus sequences with the H5N8 sequences reported from the Democratic Republic of Congo and a relatively lower identity (98.8–99.1%) with the H5N8 virus sequences reported in 2017 from Egypt and South Africa [78]. This observation suggested a bird migration-related spread of H5N8 virus in the African continent. To summarize, a high percent positivity has been reported for avian influenza virus, mamastrovirus, Nipah virus, Ntwetwe virus, and WNV from Uganda (Figure 6).

#### 3.1.15. Zambia

After an African swine fever (ASF) outbreak in Zambia during 2013–2015, 56 tissue samples were collected from 16 dead or killed pigs to determine the disease epidemiology. The outcome reported the association of three different ASFV genotypes with this outbreak, viz., genotype I, genotype II, and genotype XIV [79]. Interestingly, genotype I was most widely distributed across the outbreak-hit regions and it was found that all the genotype I virus sequences were 100% similar for the nucleotide identity, which indicated that this genotype had a common origin [79]. Later, a second ASF outbreak occurred in April 2017 in domestic pigs. A total of 15 cases of ASFV with 11 fatalities in pigs were reported in a village. The sequencing and phylogeny confirmed that this outbreak was caused by the ASFV genotype II, which was circulating in the regions of South-Eastern Africa [80]. It was suggested that the genotype II-associated outbreak in Zambia might be due to the import of swine from the neighboring country of Tanzania [79].

Serological and genetic studies based on mitochondrial (cytochrome b) and nuclear DNA analyses reported the transmission dynamics and persistence of zoonotic NiV in *E. helvum* bat populations in Zambia [60]. Five out of 12 samples collected from bats were found seropositive for NiV-specific antibodies. High allelic heterozygosity and low mitochondrial DNA diversity among bat samples from Zambia concluded that the *E. helvum* population across the sample sites in the African continent, including sites in Zambia, represented panmictic continental populations [60]. This investigation raised the concern of a higher risk of transmission of zoonotic NiV disease to human populations that may be exposed to the excreta or body fluids of *E. helvum* [60].

Since non-human primates have been found to be reservoirs of several zoonotic viruses, a recent investigation studied the prevalence of novel simian arteriviruses, pegiviruses, and lentiviruses in 12 samples of Green African Monkeys (Malbroucks) sampled from three different sites in Zambia [81]. Plasma samples were collected from these monkeys, and quantitative reverse transcriptase PCR using the specific TaqMan probes was carried out followed by deep sequencing of the positive samples. This study reported a novel arterivirus, which was termed as Zambian malbrouck virus (ZMbV-1). Two malbroucks (17%) out of 12 samples were found to be infected with ZMbV-1 [81].

A recent investigation reported the first isolation of WNV from *Culex* mosquitoes in Zambia. A total of 9439 mosquitoes were collected during 2012–2016 and were segregated into 464 pools according to species [82]. Each pool had at least one or up to 40 mosquitoes of the same species accurately identified morphologically. RNA was extracted from individual pools and subjected to a pan flavivirus RT-PCR to detect WNV, dengue virus, and yellow fever virus (YFV). Two pools of *Culex* mosquitoes which were collected during 2016 were positive for WNV using RT-PCR [82]. Sequencing and phylogenetic analysis revealed that the sequences belonged to the WNV lineage 2 strain, which was the first report of this virus from Zambia [82]. Intriguingly, a recent serological investigation reported that some human individuals had antibodies against WNV, which pointed towards the circulation of WNV in the country [83].

Domestic dogs exhibiting clinical signs of diarrhea and vomiting were investigated for canine parvovirus (CPV) infection. Thirty-two diarrheic dogs that were taken to a veterinary hospital in Lusaka, Zambia were included in the study. Fecal samples were taken from these dogs and total DNA was extracted, which was subjected to conventional PCR reaction to amplify a 583-bp region of the VP2 gene of CPV. The amplicons were gel-purified and sequenced. This study found 23 samples were positive for CPV infection mostly in unvaccinated dogs. Three variants of CPV viz., CPV-2a, CPV-2b, and CPV-2c were identified, with the highest prevalence of the CPV-2c variant in the diarrheic dogs. Interestingly, variant CPV-2a showed a 100% sequence identity at the nucleotide level with the CPV-2a variant reported from India in the year 2012 while the CPV-2c variant was highly identical to the CPV-2c variant reported from Argentina in the year 2011. Intriguingly, the study found that while all three variants of CPV were co-circulating in the country, the highest predominance was of CPV-2c, which was detected in 91.3% of the CPV-positive samples. This study reported the prevalence of the CPV-2c variant of the virus for the first time in sub-Saharan Africa [84].

In a comprehensive country-wide surveillance for rabies virus during a 16-year period from 1999 until 2015, brain tissue samples were collected and preserved from 46 domestic dogs obtained from different households, including one cat, one pig, one human, two jackals, and eight cows. Samples were subjected to the direct fluorescent antibody test (DFAT) followed by nested PCR for the detection of the N and G genes of rabies virus. Interestingly, all of the samples tested in this study were positive for rabies virus N and G genes, with only one exception where one dog was positive for the G gene and was negative for the N gene through nested PCR. Overall, this investigation showed a 100% prevalence of rabies virus in the brain tissues of dead animals and the human sample, which reflected a serious threat of rabies to humans as well as other domestic animals and wildlife in the country [85].

Fresh fecal samples from 51 healthy wild white pelicans were collected in August 2006 from Lochinvar National Park located in the Southern province of Zambia. Virus isolation followed by HI and NI subtyping identified one isolate to be avian influenza virus H3N6 subtype. For genetic analysis, the complete genome was amplified using RT-PCR. Amplicons were purified from agarose gel and sequenced using Sanger sequencing. The PB2, HA, and NS genes were highly similar with the H3N6 virus reported from a duck in South Africa, which would be due to reassortment, which might have occurred within sub-Saharan Africa because of the interaction of wild birds sharing the intra-African flyways [86]. In another investigation, 3094 fecal samples from wild waterfowls, pelicans, wild ducks, and geese were collected from Lochinvar National Park during April 2008 and November 2009. The investigation identified three subtypes of avian influenza viruses viz., H6N2, H3N8, and H11N9, in wild ducks. Subtypes of H3N8, H4N6, and H11N9 were also isolated from geese. Two avian influenza virus H3N6 and H9N1 subtypes were isolated from white pelicans. Most of the virus isolates obtained in this study clustered with virus isolates reported from wild and domestic birds in South Africa [87]. This study illustrated the transmission dynamics of the avian influenza viruses in the wild birds.

**Figure 7:**
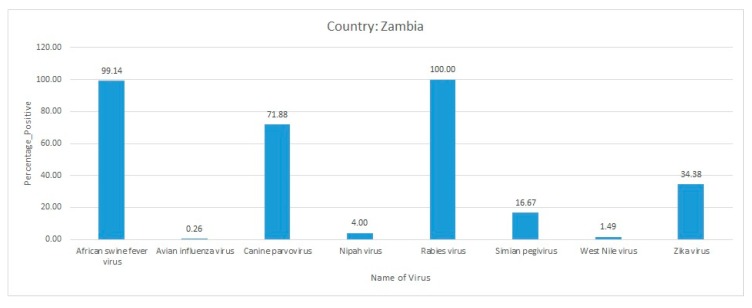
Frequency distribution of viral diseases in Zambia in selected publications.

Sera samples were collected from non-human primates in southern and eastern Zambia during 2009–2010. Forty-eight blood and spleen tissue samples were taken from Zambian malbrouck monkeys, 25 from chacma baboons, and 23 from yellow baboons. The mitochondrial cytochrome b gene was sequenced to determine the species of non-human primates. A plaque reduction neutralization test was conducted to investigate the prevalence of Zika virus, YFV, and tick-borne encephalitis virus, which resulted in a total of 33 Zika virus-positive samples. More precisely, 16 of the Zambian malbrouck monkeys, 12 of the chacma baboons, and 5 of the yellow baboons were seropositive for the Zika virus antibodies. In contrast, all spleen tissues were negative for Zika virus and YFV using real-time RT-PCR, which reflected a negative active infection. Serology also confirmed that the sera samples were negative for YFV and tick-borne encephalitis virus. The findings of this study revealed that Zika virus was in circulation among non-human primates [88]. Overall, the rabies virus, African swine fever virus, and canine parvovirus reported a high percent positivity from Zambia (Figure 7).

#### 3.1.16. Zimbabwe

Tracheal and cloacal swabs were obtained from 417 swallows (a species of wild bird) and other domestic bird populations at two different lakes near the capital city of Harare in February 2010 and October 2011. A triplex RT-PCR for the detection of avian influenza virus, avian paramyxovirus Type-1 (APMV-1), and WNV was conducted, which detected five avian influenza virus infections, 15 APMV-1 infections, and 11 WNV-positive samples in the study population. Interestingly, the birds captured for sampling appeared healthy, hence they were released successfully after sampling [89].

**Figure 8:**
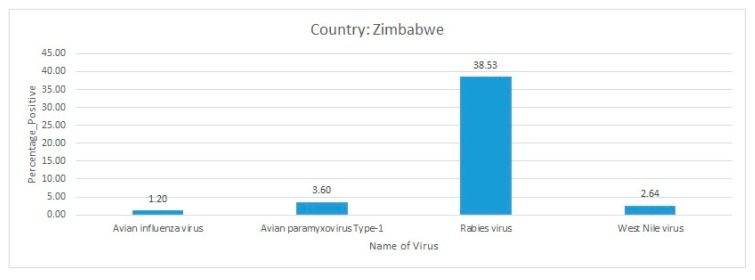
Frequency distribution of viral diseases in Zimbabwe in selected publications.

From 2010 to 2017, over an eight-year surveillance period, total of 552 brain tissue samples were collected from different animal species, including dogs, cats, bats, rodents, lions, horses, jackals, and zebras, among others, who were suspected of rabies. The brain tissue samples were submitted to the Central Veterinary Laboratory located in Harare, where samples were investigated for rabies using the direct fluorescent antibody test (DFAT) and direct rapid immunohistochemical test (DRIT). A large proportion of the tested samples (316/552) were found positive for rabies [90]. As expected, the highest prevalence of rabies was in dogs (60.13%), where 264 samples were positive out of the 439 tested. Additionally, 23 of the rabies-positive samples were further considered for virus genome sequencing using the Sanger sequencing method. The phylogenetic analysis revealed that the rabies virus sequences retrieved in this study appeared to be related to the sequences reported from south-east African countries [90]. In another comprehensive country-wide surveillance of rabies virus in Zimbabwe during 2014, brain tissue samples were collected from 13 domestic dogs from different households, as well as from one goat and 3 cows. Samples were subjected to the DFAT assay followed by nested PCR for the detection of the G and N genes of rabies virus. Interestingly, most of the samples tested in this study were positive for both the G and N genes while all of the samples were positive for the rabies virus G gene. Therefore, this investigation resulted in a 100% prevalence of rabies virus in the brain tissue samples of the dead canine, feline, and bovine animals included in this investigation. The reports on rabies virus predominance in Zimbabwe suggest a serious threat to the humans and domestic animals as well as wildlife in the country [85]. Interestingly, rabies virus was the most predominant disease reported from Zimbabwe (Figure 8).

### 3.2. West Africa

The western part of Africa includes Benin, Burkina Faso, Cape Verde, Cote d’Ivoire, Gambia, Ghana, Guinea, Guinea-Bissau, Liberia, Mali, Mauritania, Niger, Nigeria, Senegal, Sierra Leone, and Togo.

#### 3.2.1. Benin

Influenza D virus (IDV) seroprevalence was reported among cattle in Benin. IDV is a member of the *Orthomyxoviridae* family and is reported to cause a mild influenza-like respiratory disease in pigs and animals [49]. A total of 308 sera samples were collected from cattle, sheep, and goats for the screening of IDV. Only four cattle samples (1.9%) were found positive for IDV antibodies [49]. Sheep and goat sera samples were negative for IDV. Another study in 2009 included 62 swine nasal swab samples for IAV detection using RT-PCR. All the samples were negative for the IAV-active infection. Additionally, a total of 10,189 oropharyngeal and cloacal swab samples along with 100 sera samples collected from birds were subjected to IAV detection, but none of them were positive [91]. These results suggested that IAV was not circulating in the country while a low seroprevalence of IDV was identified only in cattle.

#### 3.2.2. Burkina Faso

Ruminants living in wetland areas, including ponds, swamps, and dams, in northern and central Burkina Faso were included in a sero-surveillance for RVFV. Cattle, goats, and sheep that grazed around or nearby these wetland areas were sampled between 2005 and 2007. Blood samples were collected from 120 cattle and 200 sheep and goats each. Sera were harvested and were first subjected to IgG ELISA; the positive samples were then subjected to IgM ELISA for further confirmation of seropositivity. A serum neutralization test was conducted to assess the virus-neutralizing efficacy of the RVFV antibodies. The investigation found that 18 cattle, 8 goats, and 14 sheep were seropositive for the RVFV antibodies. Interestingly, the study observed a varying seroprevalence among the sampling sites [92].

During January 2014–December 2015, 743 children up to five years of age suffering from influenza-like illness (ILI) and 181 children suffering from severe acute respiratory infection (SARI) were enrolled for treatment at six healthcare centers within Burkina Faso. Children with ILI reported a fever, cough, and sore throat. SARI cases reported having a fever, cough, and/or difficulty in breathing. RNA was extracted from the collected samples for the detection of IAV by real-time RT-PCR followed by IAV subtyping. Real-time RT-PCR samples with a cycle threshold (Ct) value up to 30 were considered for HA gene sequencing. The molecular investigation identified 112 IAV-positive children suffering from influenza-like illness while 12 children were IAV positive and suffering from the severe acute respiratory infection. Subtyping identified that 23 ILI cases occurred due to the A(H1N1)pdm09 virus, while 51 were found to be positive for the IAV subtype H3N2. Interestingly, influenza B virus (IBV) was detected in 38 of the ILI cases. The study reported that 15 ILI cases belonged to the B/Victoria lineage of IBV while 10 IBV-positive samples belonged to the B/Yagamata lineage, but 13 other samples could not be ascertained. Similarly, seven cases of the SARI were due to IAV infection, five were because of IBV infection, two were due to the A(H1N1)pdm09 virus, and another five were due to the H3N2 virus infection. This study reported that different types and strains of influenza viruses were responsible for inflicting ILI and SARI among children in Burkina Faso [93].

Thirty tracheal swabs as well as 10 organ samples were submitted from a poultry farm in Burkina Faso upon suspicion of infectious bronchitis virus in the flock, which reported a decrease in egg production along with respiratory disease symptoms. Molecular diagnostics identified that the flock at the poultry farm was infected with IAV H9N2 subtype and was negative for infectious bronchitis virus. The phylogenetic analysis revealed that the H9N2 strain belonged to the G1 lineage, which is known for its high zoonotic potential. The IAV H9N2 subtype which was reported from the poultry flock in Burkina Faso clustered with H9N2 isolates reported earlier from the United Arab Emirates in 2015 and Morocco in 2016 [94].

#### 3.2.3. Cape Verde

After an influenza-like illness occurred in the country during 2009–2010, a large-scale surveillance was conducted for the detection of influenza viruses. Nasopharyngeal and oropharyngeal swabs were collected from 498 symptomatic patients; extracted viral RNA samples were subjected to the molecular detection of IAV and IBV using one-step real-time RT-PCR assays. Only IAV-positive samples were further subjected to a second subtype specific real-time RT-PCR assay to distinguish among H1, H3, and A(H1N1)pdm09 viruses. Out of 498 samples, 131 samples were found to be positive for IAV while only one sample was positive for IBV. More precisely, only eight patients were found to be infected with the IAV subtype H3N2 virus while 123 patients were positive for the A(H1N1)pdm09 virus. Interestingly, no patients were positive for the IAV subtype H1N1. The HA1 gene sequences were retrieved from one of the isolates, which phylogenetically clustered together with the sequences reported from the USA and China. [95].

#### 3.2.4. Cote d’ Ivoire

Zoonotic transmission of Ebola virus was reported from Cote d’ Ivoire in 1994 from chimpanzees to humans [71]. There are four African strains of Ebola virus reported so far: Zaire Ebola virus, Ebola virus Sudan, Ebola virus Ivory Coast, and Ebola virus Bundibugyo [71]. Ebola virus infection is known to cause hemorrhagic fever in humans, with high fatality. Apart from Ebola virus, Cote d’Ivoire has also been hit by monkeypox. Up to 1990, two confirmed human cases of MPXV had been reported from the country [96,97]. One common practice that has been reported from the MPXV outbreak-prone belt of the African continent is that monkeys are eaten as a delicacy, which may be a significant source of disease transmission apart from other activities, like hunting and butchering.

A Western blot and PCR-based investigation revealed frequent infection of simian foamy virus (SFV) in free-living chimpanzees in Tai National Park. This study reported 12 chimpanzees having SFV infection out of 14 samples [98]. This study also suggested a high risk of interspecies transmission of the virus in the nearby rural population. In another investigation, HIV-1 and HIV-2 lineages were reported in the rural population surrounding the Tai National Park while simian immunodeficiency virus (SIV) infections were reported from the monkeys. Interestingly, an eight-year-old boy was reported to have fed upon bushmeat and was found positive for HIV-2; which would be the most probable source of infection [99].

In a surveillance for influenza virus infections among human population during 1999–2014, 344 cases of A (H1N1)pdm 09 virus, 171 human infections of H3N2 subtype of IAV, and 446 cases of IBV infections were reported [52]. Interestingly, no human cases of H1N1 influenza viruses were reported from the country in this period. Another investigation during 2009–2010 which included a total of 12,493 oropharyngeal and cloacal swab samples from birds, which were subjected to IAV diagnostics using the RT-PCR assay, and it was reported that all tested samples were found to be negative for the IAV-active infection. Additionally, 1283 sera samples were collected from birds and subjected to serological testing using ELISA and HI assays. All the sera samples were also found to be negative for the IAV antibodies. Further, 457 pig sera samples were subjected to ELISA, HI test, and RT-PCR but none were found to be positive for the IAV infection. Additionally, out of 1548 swine nasal swab samples that were tested, none were positive for the IAV infection [91]. These findings suggested a lack of prevalence of IAV during the study period.

In a large-scale surveillance of Newcastle disease virus (NDV) and infectious bronchitis virus during 2010–2012, tracheal and cloacal swab samples were collected from 14,508 backyard chickens, 7322 commercial chickens, 650 ducks, and 326 guinea fowls. Additionally, 1943 sera samples were also collected from different birds. Infectious bronchitis virus antibodies were detected in 1401 sera samples using the ELISA test while 420 sera samples had NDV antibodies using the HI test. Nested PCR was conducted using random hexamers to assess the prevalence of NDV and infectious bronchitis virus in tracheal and cloacal swabs, which detected 363 and 272 infectious bronchitis virus-positive pools in the backyard chickens and commercial chickens, respectively. Sixteen pools from duck samples and 15 pools from guinea fowl samples were also positive for the infectious bronchitis virus infection. On the other hand, 421 pools from backyard chicken samples while 230 pools from commercial chicken samples were positive for the NDV disease. Fourteen pools from ducks and five pools from guinea fowls were positive for NDV. Therefore, this investigation identified NDV and infectious bronchitis virus in backyard and commercial poultry, ducks, and guinea fowls during 2010–2012 [100].

**Figure 9:**
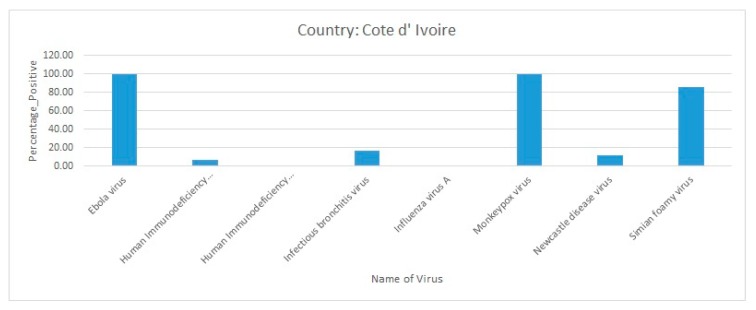
Frequency distribution of viral diseases in Cote d’Ivoire in selected publications.

Briefly, Ebola virus, MPXV, and SFV infections were predominantly present in Cote d’Ivoire. The other comparatively less predominant virus diseases that were circulating in the country were HIV-1, infectious bronchitis virus, and NDV (Figure 9).

#### 3.2.5. Gambia

A study was conducted to screen the incidence of hepatitis B virus (HBV) in pregnant women visiting the maternity department of Edward Francis Small Teaching Hospital in Gambia between 1 May and 31 July 2015. Blood samples were collected from 424 pregnant women during the study period. The seroprevalence of HBV was determined by screening for HBsAg. Interestingly, 39 (9.2%) samples were found to be positive for HBsAg; however, only eight women reported a history of jaundice. Additionally, the sera samples were simultaneously tested for HIV infection. Only three women were found to be positive for HIV [101].

#### 3.2.6. Ghana

Nasal swab samples from 50 pigs were collected at a Kumasi abattoir during January-February 2014. Samples were investigated for IAV infection using H3N2-specific anti-HA monoclonal antibody ELISA. Two samples (4%) were found to be positive for H3N2 antibodies [102]. In another study, nasal swab samples collected at a Kumasi abattoir during January to March 2014 were investigated for A(H1N1)pdm09 virus, which identified five A(H1N1)pdm09 virus-positive samples [31]. This finding speculated that there might be an incidence of zoonotic transmission of A(H1N1)pdm09 to the exposed swine workers [31]. In a more recent investigation, 19 pig handlers working either at an abattoir or piggeries were enrolled for an investigation aimed at the detection of IAV. Simultaneously, nasal swab samples from 132 pigs were collected from an abattoir and piggeries in Kumasi during 2014–2015. The one-step RT-PCR for matrix gene detection followed by HA subtyping using one-step RT-PCR confirmed that two swineworkers were infected with A(H1N1)pdm09 virus. One of these infections was from an abattoir worker while the other infection was from a piggery worker. Interestingly, 13 pig samples were also found to be positive for A(H1N1)pdm09 virus while three pig samples were infected with H3N2 virus. These findings suggested a probable zoonotic transmission of A(H1N1)pdm09 virus between swine handlers and the pigs under investigation [103].

Forty-eight samples, including tracheal swabs and tissues from the lung, brain, spleen, and trachea, were collected from 44 different poultry farms between November 2017 and January 2018. Samples were tested for IAV using the RT-PCR with matrix gene as well as for H5, H7, and H7 subtypes along with IBV and NDV. In this investigation, seven complete IAV genomes and one sequence of the HA cleavage site of IAV were successfully recovered along with one partial S1 gene of IBV. Sequence analyses identified the H9N2 subtype of IAV in the poultry samples. Additionally, H9N2 virus was successfully isolated from two of the samples [104]. A highly pathogenic strain of avian influenza virus H5N1 was reported from symptomatic chickens after an outbreak in the Accra region. A high mortality rate of 17.6% was reported in chickens. Sequencing and phylogenetic analyses of the different genes identified that the strain responsible for the avian influenza outbreak in chickens in Ghana belongs to the same clade responsible for the Nigerian avian influenza outbreak of 2015 [14].

Lassa fever is a viral hemorrhagic fever caused by Lassa virus, which has an incubation period of 1–3 weeks. The initial symptoms include fever and weakness in the early stage followed by headache, muscle pain, sore throat, diarrhea, and abdominal pain in the following days. The advanced stage symptoms may include seizures, disorientation, shock, and even coma [105]. The first case of Lassa fever in Ghana was a 19-year-old male who was a farmer and an active hunter. He felt febrile and had chills along with joint pain for about three days. He went on hunting with another household member and had a rat meal while he was symptomatic. His condition worsened over the next three weeks and he was hospitalized, where his condition became complicated and he died. The RT-PCR investigation of the patient’s blood confirmed an infection with Lassa virus [105]. Interestingly, the second case was a 24-year-old male who had no contact with the first patient. He developed fever and had yellowish sputum with chest pain. Soon, his condition deteriorated, and he started vomiting blood and bloody sputum, following which he died in the hospital within hours. His blood sample was analyzed and found to be positive for Lassa virus infection [105]. These two cases indicate that Lassa fever is a fatal hemorrhagic zoonotic virus disease and suggested that contact with or eating rats may trigger zoonosis.

The straw-colored fruit bat (*Eidolon helvum*) has been reported to be a reservoir for several zoonotic virus infections, including those of henipaviruses. This bat species has been documented to exist in proximity of human populations in Accra [60]. An investigation reported the detection of 369 straw-colored fruit bat samples that had Nipah virus antibodies [60]. The tendency of this bat species to feed nearby human populations put humans at risk of potential exposure to its excreta, body fluids, and tissues, which raises the concern of zoonosis and public health. In another study, urine samples of from straw-colored fruit bats were collected from Accra during September to November 2010, which were used for virus isolation. Sera samples were also collected at two different times in 2007 and 2010. Sera were also collected from humans living nearby bat roost in Accra or from those who were involved in the hunting or butchering of bats. Novel paramyxoviruses named AchPV1 and AchPV2 were isolated from the bats [67]. To assess the zoonotic potential of the Achimota virus, 216 healthy human subjects were sampled. Sera of two febrile individuals reacted against the AchPV2 antibodies, which indicated the zoonotic potential of this novel bat-borne paramyxovirus (Achimota virus) and its ability of cross-species transmission to humans [67].

Human cases of rabies were recorded in Ghana. A total of 13 individuals comprising medical doctors, a nurse, a pharmacist, a physician assistant, a veterinary doctor, and technical officers had a history of either being bitten or scratched by rabid dogs [106]. There was one individual who reported feeding on a dog that displayed rabies symptoms before its death. All the reported human cases died of the disease [106].

The archived human sera samples from a 2014–2016 Ebola virus surveillance were tested for dengue virus using anti-DENV IgG/IgM ELISA assays. A total of 150 sera samples were first tested with ELISA. Then, 32 anti-IgM ELISA-positive samples were further tested with real-time RT-PCR using total RNA extracted from these samples, which confirmed four dengue virus-positive samples out of the 32 tested. These four patients, comprising two males and two females in the range of 30–56 years of age, had unexplained bleeding of the gums, muscle and/or joint pain, with fever and headache. Three of the patients belonged to southern Ghana while the fourth belonged to Burkina Faso, who sought medical help in the northern part of the country. The genome sequences of the RT-PCR-positive dengue virus cases showed similarity to the sequences reported from Senegal and India. This study reported two different dengue virus serotypes in circulation in Ghana among Ebola virus-suspected cases during 2014 to 2016 [107]. Further, 160 archived sera samples from patients who reported fever with arthritis or conjunctivitis or arthralgia between December 2016 and November 2017 in a hospital in Accra were tested for Zika virus anti-IgM and anti-IgG ELISA tests. Interestingly, 30 sera samples were found to be positive for IgM antibodies while three samples were positive for IgG antibodies, resulting in an overall seroprevalence of 20.6% (33/160 patients) [108]. This proved that the patients had Zika virus infection at a certain time in the past.

#### 3.2.7. Guinea

Until 20 April 2014, 208 cases of Ebola virus disease were reported from Guinea, out of which 136 died, resulting in a very high rate (65.38%) of mortality. It was reported that the disease spread through human-to-human contact and siblings within households were found to be positive for the Ebola virus [109]. The disease affected people from different age groups and across different occupations. The 2014 Ebola epidemic was reported to be the fourth largest epidemic in the African continent [109]. Until 4 May 2015, 3529 cases of Ebola were reported from Guinea. It was reported that a large number (892 or 25.3%) of the cases had a history of contact with those who were infected with the disease [110]. This contact happened either at funerals or within the household. Forty people (4.5%) from Guinea reported having contact at the funeral with the corpse, which was the most likely event to transmit the disease, while 571 (64%) people reported an exposure during a non-funeral context, which was basically either in the household or at work. Interestingly, 281 (31.5%) cases reported both types of exposure, funeral and non-funeral [110]. This investigation made it very clear that the Ebola virus disease is highly zoonotic and has great potential to spread among the population exposed to it by any means.

In a rare case, an Ebola virus transmission from mother to child was reported in Dubreka. In August 2015, a nine-month-old female infant developed fever, vomiting, cough, and diarrhea. After preliminary symptomatic treatment, her condition was stable for the next five days, which later deteriorated with severe diarrhea and vomiting. The infant quickly developed respiratory distress while being shifted to the University Hospital but died on the way. Her buccal swab test was positive for Ebola virus by RT-PCR. The genome of this virus matched with the Sierra-Leone 3 lineage, which was endemic in the region of her residence during May–July 2015 [111]. To trace the source of infection, the deceased infant’s parents were tested for Ebola virus infection using body fluid samples. Surprisingly, the mother’s breast milk was found to be positive for Ebola virus RNA using real-time PCR with a threshold cycle (Ct) value of 23.3. Following sequencing and phylogenetic analysis, it was found that the virus from the breast milk was closely related to the virus from the infant’s buccal swab. More interestingly, both isolates shared two unique single-nucleotide polymorphisms [111]. Hence, it became evident that the transmission happened to the infant through breast feeding from the mother. RT-PCR-based diagnostics confirmed that the virus was also present in the seminal fluid of the father of the infant. Sequencing revealed that this virus was part of the Sierra-Leone 3 lineage but was not close to the virus isolates from the infant and the mother [111]. This was a rare incident reporting the Ebola virus transmission to an infant from the mother via breast feeding.

In October 2015, another case of Ebola virus disease appeared from Conakry, where a case patient had a loss of appetite with fever and started bleeding from the nose four days after being symptomatic. He shared a small household with a relative who was an Ebola virus disease survivor. An RT-PCR-based investigation revealed that he was positive for the Ebola virus. Since it was a known fact that Ebola virus can be shed in survivors’ semen for a long period of time [112], therefore, in this case, sexual transmission of the virus within the marriage was suspected. Upon investigation, the woman’s serum was found to be positive for the Ebola virus antibodies with the ELISA test. The genome sequence of this virus revealed that the virus did not belong to the circulating clade of Sierra-Leone 3 in that area; rather, it belonged to another lineage GN1 [113]. Hence, the findings of this study inferred that in this case, the survivor most likely transmitted the Ebola virus to the woman sexually. Since these people within the same household shared facilities under unhygienic conditions, this would be the most probable reason of disease transmission to the case [113].

In another study, 76 days after the WHO announced the end of the Ebola epidemic in Guinea, three deceased probable Ebola virus cases came to the notice of the authorities during 27 February to 15 March 2016 [114]. The investigation learned about new cases of the disease, which were transmitted among the family members of the three patients who recently died of the disease. By the end of April 2016, there were seven confirmed and three likely cases of the Ebola virus disease in the region, eight of which died during treatment. Since these people lived in the forest region, a spillover event from a wild animal reservoir was suspected. To rule out any speculations, the blood samples from two confirmed cases were collected and sequenced. The analysis confirmed that neither sequences belonged to the Sierra-Leone 3 nor GN1 clusters but were associated with the sequences reported during the 2014 outbreak [114]. Hence, these results ruled out the possibility of transmission from an animal reservoir. Further investigation revealed that one of the survivors had sexual contact with the deceased first case as well as other sexual partners. It was noted that this survivor’s seminal fluid sample was positive for Ebola virus, with a Ct value of 23.4 on 21 March 2016, which was again positive with a Ct value of 32.4 on 9 April 2016. The survivor’s seminal fluid was collected on 21 March 2016 and viral RNA was sequenced; the results showed identical sequences from both survivors. Sequences from the other cases confirmed that the disease cluster was linked to the survivor, who sexually transmitted the disease to other individuals. Intriguingly, the molecular assays and sequencing provided evidence that the Ebola virus was persistently present in the survivor’s seminal fluid between 26 October 2014 and 9 April 2016. This was new information indicating that Ebola virus was persistent in the seminal fluid of the patient who was cleared of infection following the standard protocol even after 531 days of the first diagnosis, the longest period reported so far [114].

A surveillance study reported 22 confirmed cases of Lassa virus infection in Guinea out of 311 suspected cases. An 18% fatality rate was reported for the Lassa fever cases [115]. The most common disease symptoms included a sore throat, edema, and sudden hearing loss, among others. Interestingly, 43 cases had seroprevalence for the Lassa virus IgG antibodies, which was an evidence for past exposure of these cases to the disease [115]. Another surveillance study was conducted to find out the animal reservoir of the Lassa virus in Guinea [116]. Across seven bush sites, 1616 small mammals, mainly including rodent species of *Mastomys* and *R. rattus* among others, were captured from households across the study area. In total, 96 rodents were found to be positive for Lassa virus antibodies in their sera samples while 46 were detected with Lassa virus antigen in the sera [116]. This study suggested that Lassa virus may transmit to human populations from these infected rodent species found in households.

After the outbreak of an influenza-like illness, a surveillance that included the collection of nasopharyngeal and oropharyngeal swabs was conducted during 2009–2010. Clinical samples from 166 symptomatic patients were collected. This study identified five IBV-positive patients while 15 patients were found to be infected with IAV. More precisely, nine patients were found to be infected with A(H1N1)pdm09 virus while six patients were positive for influenza virus H3N2 subtype. The IAV and IBV isolates retrieved in this study could not be sequenced given the poor quality of the clinical specimen and virus isolation [95].

#### 3.2.8. Guinea-Bissau

A new subtype of human T-lymphotropic virus type 1 (HTLV-1) subtype 1g was reported from rural Guinea-Bissau [117]. It is believed that HTLV-1 originated in Africa from monkeys in the wild and was transmitted to humans as a result of animal–human contact due to the social and cultural practices in rural communities. Rural Guinea-Bissau has been reported to have a 5% HTLV-1 prevalence in West Africa, which is the highest known prevalence in the region. Unfortunately, due to a lack of genomic data, information on the circulating subtypes of the virus was unknown until recently. The pattern of transmission within communities was also unknown. Hence, to find out the disease epidemiology and transmission patterns of HTLV-1, adults and children from the Caio community in the country were sampled. The status of HTLV-1 and HTLV-2 was determined through the ELISA test. The outcome was further confirmed with PCR using tax/rex gene-specific primers. Additionally, the long terminal repeat (LTR) and p24 regions of the virus genome were sequenced for 72 individual samples to analyze the disease [117]. Based on the LTR sequences, phylogenetic investigation confirmed the presence of the HTLV-1 subtype 1a in majority of the Caio sequences. However, interestingly, one virus sequence was divergent and was found to be closely related to the HTLV-1 subtype 1g, which was basically isolated from a hunter in Cameroon. This finding was further supported by analyzing the p24 sequences obtained from 36 individuals [117]. This was the first report of a novel HTLV-1 subtype 1g from Guinea-Bissau and it was speculated that transmission probably happened either through human-to-human contact or from the wild.

#### 3.2.9. Liberia

Until 4 May 2015, a total of 5343 Ebola cases had been reported from Liberia. Interestingly, 2078 cases (38.9%) had a history of contact with those who were infected with the disease [110]. These contacts happened either at funeral events or within the household. Forty-nine people (2.4%) reported having contact at funeral events with the corpse, which was the most likely reason to transmit the disease, while 1717 (82.6%) people reported an exposure during a non-funeral context, which was basically either in the household or at work. Interestingly, 312 (15%) cases reported both types of exposure, funeral and non-funeral [110]. The findings of this study showed that Ebola virus disease is highly zoonotic and has great potential to spread among the population exposed to it.

In March 2015, a case of sexual transmission of Ebola virus from a male partner to a female was reported from Montserrado County. There was a male survivor of the Ebola virus disease whose brother and estranged wife were also positive for Ebola virus in 2014. The survivor male had undergone several tests, including blood and semen, at different intervals to assess the status of the disease. The survivor’s seminal fluid and blood were negative for Ebola virus 231 days after the estimated onset of the Ebola virus disease. Surprisingly, the TruSeq RNA Access kit (Illumina, CA, USA) using custom capture probes achieved 85.1% of the genome coverage of Ebola virus from the semen of the male survivor. The nucleotide sequence alignment of the genomes retrieved from the survivor’s semen and his female sexual partner was done and it was found that there was only one base-pair mismatch across the 15,808 nucleotides, which was a strong evidence of sexual transmission of Ebola virus from this male survivor to his female partner [112].

In June 2015, the case of a 17-year-old deceased boy from Margibi County triggered the alarm of a second Ebola flare-up in Liberia 101 days after the last detected case of Ebola virus disease. Following this, at least seven other cases having Ebola-like symptoms were reported from the region, six of whom were found positive for the disease using real time RT-PCR analysis [118]. Further investigation explored that two of the Ebola patients from this outbreak had relatives who were survivors of the disease and had spent time residing on a nearby farm. Interestingly, it was noted that nine of the 22 occupants of this farm were infected with Ebola, and eight of whom had died, leaving behind a minor survivor. This study suggested that human-to-human transmission through a subclinical but persistent infection was the most likely source of this Ebola flare-up in Liberia [118].

Apart from Ebola virus disease, at least two MPXV disease events have been reported from Liberia so far. The first event of disease confirmed four cases during the period of 1970–1990 [96]. Almost four decades after the last reported cases of MPXV disease, 16 suspected cases of the disease were reported during November-December 2017, out of which two cases were confirmed [97,119].

#### 3.2.10. Mali

Lassa fever is an important zoonotic disease found in Western African countries. The rodent species of *Mastomys natalensis* has been reported to transmit the disease to humans. In an investigation, out of 793 individual rodent samples collected in sub-Saharan Mali, *Mastomys natalensis* was amongst the most frequently captured rodent species. In total, 35 of the 511 tested *M. natalensis* sera samples were positive, with a seroprevalence rate of 6.8% [120]. Interestingly, the study findings revealed that a 14.2% seroprevalence rate was found in the *M. natalensis* population captured from southern Mali, especially the surrounding villages nearby Soromba. Real-time and conventional RT-PCR analyses revealed a prevalence rate of 7.7% (19/246). Intriguingly, ˃99% of the rodents captured from southern Mali were *M. natalensis* species [120]. This observation suggested the probable endemic nature of the Lassa virus pathogen. A significantly low biodiversity may facilitate more efficient transmission of the virus to the human population [121]. Despite a large population of infected rodents in the region, only one human case of Lassa virus disease was reported. Since the rodents in this study were mostly captured from households, including kitchens and bedrooms, the humans were reported to be at great risk of an outbreak [120].

A cohort of 202 rural inhabitants of Mali were investigated for the seroprevalence of A(H1N1)pdm09 virus using the HI assay. Sera samples were collected from 202 rural individuals living in eight different villages during the year 2006 and later in April 2010. The HI assay identified 30 individuals that were positive for the A(H1N1)pdm09 virus in this investigation. This study reported the seroprevalence of A(H1N1)pdm09 virus in the rural population of Mali [122].

#### 3.2.11. Mauritania

During September to November 2015, a hemorrhagic fever outbreak occurred in Mauritania. The first symptomatic case was tested for Ebola virus through RT-PCR, which was found to be negative. Hence, further diagnostics for RVFV, CCHFV, dengue virus, chikungunya virus, WNV, and YFV was conducted using either ELISA or RT-PCR. After this, 10 more cases were investigated for the above viruses. As a result, an RVFV outbreak was declared, as all the suspected cases were positive for the RVFV infection. Following this declaration, a surveillance was conducted and the patients reporting fever, back pain, nausea, vomiting, diarrhea, and cutaneous bleeding were sampled and tested for the differential diagnostics for RVFV, CCHFV, and YFV using anti-IgM ELISA and RT-PCR. This investigation included all 11 samples before the outbreak was announced and 173 samples that were collected after the outbreak was declared. Therefore, a total of 184 samples were investigated for several arboviruses. As a result, 57 samples were found to be positive for the RVFV, 5 samples were positive for CCHFV, 27 samples were positive for dengue fever virus, and one sample was positive for WNV. There were some samples that were co-infected with two viruses. The RVFV-positive samples were partially sequenced and a phylogenetic analysis was conducted, which revealed a possible association between the observed cases in Mauritania and the RVFV isolates reported from Senegal in 2013. The RVFV disease outbreak in Mauritania reported 57 confirmed cases as well as 12 deaths [123].

After the outbreak of an influenza-like illness during 2009–2010, a large-scale surveillance that included the collection of nasopharyngeal and oropharyngeal swabs was conducted. Clinical samples from 227 patients were collected, and RNA was extracted for the diagnostics of influenza viruses. RNA samples were subjected to the molecular detection of IAV and IBV using specific oligonucleotides with one-step real-time RT-PCR assays. Only IAV-positive samples were further subjected to a second real-time RT-PCR assay to distinguish among the H1, H3, and A(H1N1)pdm09 viruses. Virus isolation was attempted in Madin-Darby Canine Kidney (MDCK) cells followed by virus subtyping using the HI assay with specific antisera. The HA and NA gene sequences of the selected viruses were obtained through cycle sequencing followed by sequence alignment and phylogenetic analysis for virus characterization. The investigation revealed that a total of 39 patients were positive for IAV while only three were positive for IBV. Further, 26 patients were found to be infected with A(H1N1)pdm09 virus while seven samples were positive for IAV H3N2 subtype. Six samples were also found to be positive for IAV H1N1 subtype while only three samples were positive for IBV. Interestingly, based on the HA gene sequences that were retrieved in this study, five of the IAV isolates clustered together with the sequences reported from Uganda and southern Russia [95].

#### 3.2.12. Niger

Rift Valley fever outbreak was reported from Niger during July–September 2016. As a result, many ruminants, including cows, goats, and sheep, were reported to have fever, increased salivation, abortions, and death during the outbreak. An investigation was soon launched after the disease symptoms in ruminants were reported in the country. In the early phase of the investigation, sera samples were collected from 13 symptomatic humans as well as 6 animals, including one camel, one cow, 3 goats, and one sheep. The collected sera samples were tested for a panel of arboviruses, including chikungunya virus, dengue virus, YFV, and WNV, as well as hemorrhagic fever viruses, including CCHFV and RVFV. Three animals, including one cow, one goat, and a sheep, were found to be positive for RVFV through the RT-PCR assay. Further, during August–December 2016, a total of 399 human sera samples were collected. RT-PCR and ELISA tests identified 17 RVFV cases. More precisely, nine sera samples were found to be positive with RT-PCR while eight samples were positive for ELISA using specific antibodies. Strikingly, 33 human subjects under investigation were reported dead during this period. This was the first report of RVFV from Niger. The findings of this investigation showed the high probability of virus zoonosis between ruminants and the exposed human population in the region. Interestingly, the study also suggested that large human gatherings during festivities where ruminants are traditionally sacrificed along with environmental factors like wet climatic conditions may potentially aggravate the spread of the disease [124].

#### 3.2.13. Nigeria

The prevalence of H5N1 in poultry in Nigeria has been known since 2006, but the incidence of human infections was unknown. Hence, an investigation was carried out to determine if the agricultural workers exposed to poultry had evidence of disease transmission. The study included 316 farm and open market workers from four towns in the south-western part of Nigeria who were exposed to domesticated poultry, including chickens, geese, ducks, pigeons, and turkeys [125]. The MN assay showed eight poultry-exposed workers and two non-exposed individuals had elevated antibody titers against three subtypes of avian influenza viruses viz., H5N1, H5N2, and H11N1, and one avian-like influenza virus H9N2 subtype [125]. Interestingly, one non-exposed individual was seropositive for IAV H5N1 subtype, which indicated the possibility of subclinical avian influenza H5N1 infection in the past. One poultry-exposed worker was seropositive for two viruses: H11N1 and H9N2, which indicated that this person was infected with two influenza viruses at some point of the occupational exposure. The study had three categories of exposed individuals: poultry industry workers, meat processors, and poultry market workers. These individuals had been in their professions from three to 20 years. This investigation reported the zoonotic transmission of avian influenza virus from domestic poultry to humans, but more interestingly, the findings also suggested that subclinical infection with avian influenza virus may also trigger zoonotic transmission of the disease [125].

Over a two-year period during July 2010–June 2012, a total of 227 clinical cases of swine influenza were observed among the swine population in Lagos. Nasal swabs along with blood samples were collected from pigs and pig handlers who participated in this study. Viral RNA was extracted from nasal swab samples and sera was separated from the blood samples for downstream processing. The human workers and the animals included in this study were observed for any clinical signs of respiratory distress as well as fever and coughing. Viral RNA was subjected to one-step matrix gene real-time RT-PCR for the detection of IAV. No human samples were found to be positive for IAV infection. Interestingly, 31 swine samples were found to be positive. The virus was successfully isolated from 29 swine samples. These 29 samples were further investigated for subtyping, which confirmed the presence of A(H1N1)pdm09 virus in 18 swine samples. This was the first report of the prevalence of A(H1N1)pdm09 virus in Nigerian pigs [23].

Soon after, another investigation from December 2015 to February 2016, during the period of circulation of highly pathogenic avian influenza H5N1 virus subtype in poultry in Nigeria, 129 apparently healthy pigs were sampled for an investigation of the incidence of the disease. Surprisingly, real-time PCR analysis confirmed that 43 swine samples were infected with the IAV [30]. Intriguingly, clade-specific real-time PCR determined that 22 swine samples represented the H5N1 clade 2.3.2.1c; the phylogeny confirmed the transmission of the virus from avian species to the swine. This was the first report of the H5N1 prevalence in a swine population from Nigeria [30]. The mechanism of transmission of avian-like influenza H5N1 virus subtype to the swine population may be explained by the introduction of the virus into the swine population through wild birds. The lack of proofreading as well as post-replication repair mechanisms due to error-prone RNA polymerase, which are known to make IAV a highly versatile virus in terms of genetic reassortment and evolution, may facilitate the adoption of avian-like influenza viruses into swine, where it would evolve through reassortment [30,126].

Seventy-five nasal swabs from pigs at a municipal slaughterhouse in Bodija and at the University of Ibadan’s Teaching and Research Farm were collected and investigated for IAV. The assay revealed that three pig swab samples were positive for IAV H3N2 subtype [102]. Further, six additional pigs tested positive for the A(H1N1)pdm09 subtype [31]. This study suspected a possibility of zoonotic transmission of A(H1N1)pdm09 virus to the exposed swine workers [31]. Interestingly, one investigation identified 14 samples to be IAV H1N1 subtype and 23 samples to be IAV H3N2 subtype from animal workers in a swine farm [127]. In another study, 218 swine nasal swab samples from a public abattoir in Ibadan and from nine nearby piggeries were collected. The selected pigs had no symptoms of any respiratory disease [6]. Twenty-four samples were detected as IAV positive for matrix gene-based RT-PCR. The HA subtyping RT-PCR confirmed that 19 of these samples were A(H1N1)pdm09 viruses while five samples were IAV H3N2 subtype. The matrix gene of the IAV-positive samples were amplified and sequenced. The phylogeny revealed that the M gene sequences of A(H1N1)pdm09 virus recovered in this study clustered with A(H1N1)pdm09 viruses that were in circulation among humans during 2011–2013 [6]. These findings suggested a reverse zoonotic transmission of A(H1N1)pdm09 viruses from humans to pigs.

In a more recent investigation during 2014–2016, 68 pig handlers working either at the public abattoir or in piggeries were enrolled for an IAV surveillance study. In addition to the pig handlers, swine samples were also collected during this investigation [6]. As a result, one abattoir worker in Ibadan was found to be infected with A(H1N1)pdm09 virus. Nineteen pigs were also found to be infected with A(H1N1)pdm09 virus while five pigs were infected with IAV H3N2 subtype. Hence, this investigation provided an evidence of zoonotic transmission of the A(H1N1)pdm09 virus from the pigs to the abattoir worker [103].

Another study was undertaken to assess the prevalence of IAV in pigs and piggery workers in two communities in Lagos. Nasal swab and sera samples from 197 piggery workers as well as 281 pigs were collected from both communities in this study. One-step RT-PCR revealed that all the human and swine nasal swab samples were negative for IAV. Interestingly, 171 human sera and 188 swine sera samples were found to be positive with influenza A IgG antibodies using an ELISA assay. This study presented the evidence of past exposure of the swine and piggery workers to IAV infection [128].

In another investigation, sera samples were collected from swine herds at swine farms and at slaughterhouses. A total of 264 sera samples from 31 swine herds were collected during 2009 and 627 sera from 75 herds were collected during 2012. The majority of the pigs appeared healthy at the time of sampling, with the exception of five pigs that exhibited sneezing, nasal discharge, and weakness. All sera samples were tested by the virus microneutralization assay using MDCK cells, given its higher sensitivity over HI test where HI assay has too much cross-reactivity from human viruses and is worthless for swine viruses. The results found that during 2009, only one swine herd was positive for the A(H1N1)pdm09 virus while the samples taken during 2012 resulted in 66 A(H1N1)pdm09 virus-positive swine herds, which suggested that most of the swine herds were free from influenza in 2009 but later became infected. On the contrary, eight herds were positive for IAV H1N1 subtype while four swine herds were positive for human-like H3N2 virus during 2009. Intriguingly, during 2012, a study found that 53 swine herds became infected with H1N1 virus while 66 herds were found to be positive for A(H1N1)pdm09 virus, which was a sharp increase compared to 2009 [129].

A sero-surveillance was conducted to assess the seroprevalence of canine influenza virus H3N8 and H3N2 in pet dogs and village hunting dogs in Oyo, Lagos, and Ogun provinces of southwestern Nigeria. Approximately 3 mL of blood were collected from 96 pet dogs as well as from 89 village dogs living in hunting communities. The HI assay using specific antibodies detected that 75 dogs were seropositive for canine influenza virus H3N8 antibodies while none of the dogs were positive for canine influenza virus H3N2 subtype. However, most of the pet dogs sampled at Ibadan and Lagos veterinary clinics were vaccinated for rabies virus and canine distemper virus, but the seroprevalence of canine influenza virus H3N8 subtype in pet and village dogs puts the companion human population at risk for disease transmission [130].

Several cases of MPXV disease have been reported from Nigeria. The first known case appeared in an eleven-year-old boy in the year 1971 who developed MPXV disease symptoms, including a rash, sore throat, fever, and headache, among other symptoms. Subsequently, other family members contracted similar disease symptoms, but the epidemiology of the disease could not be ascertained. Later monkeypox outbreaks in the country reported at least 262 suspected cases, out of which 7 patients died while another 115 cases were confirmed for the MPXV disease [119,131]. The investigations reported that these outbreaks were not imported but most probably resulted due to a spillover from a possible reservoir host within the country. According to a recent report, during 1971 to 2018, a total of 118 confirmed cases and seven deaths due to MPXV disease were reported [132]. Another study reported that a third outbreak of the MPXV disease occurred in September 2017, during which 61 cases were confirmed out of 172 suspected cases across 14 states [4]. WHO reported 244 suspected cases of MPXV disease; 101 cases were confirmed during 2010 to 2018 [133]. It has been suggested that the cultural and traditional practice of bushmeat hunting and trade puts individuals at risk of disease transmission from wild animals to humans. Apart from this, exposure to the body fluid of infected individuals is an important factor for human-to-human transmission of the disease [4].

From 1964 to 1970, a total of 12,613 specimens were examined for the screening of virus diseases at the University College Hospital in Ibadan [134]. Serologically, 171 samples were found to be positive for different viruses, including the most common arboviruses. A total of 16 specimens were positive for YFV, three were positive for Zika virus, 56 were positive for chikungunya virus, and 57 specimens were positive for dengue virus subtype 1 and 2 [134]. A seroprevalence study confirmed 16 positive cases for dengue virus, 17 for WNV, and another 16 were positive for YFV disease. These reported arboviruses are usually vectored by mosquitoes and the abundance of mosquitoes in the areas where the study was conducted suggested the transmission of these diseases to humans through mosquito bites [35].

A high seroprevalence of MERS-CoV was reported in dromedary camels during 2010–2011. In total, 358 sera samples were collected from dromedary camels, which were raised for the purpose of meat production, from abattoirs located in four provinces viz., Adamawa, Borno, Kano, and Sokoto. Serology based on IgG antibodies detected MERS-CoV in 94% of the sera samples (336/358). The study illustrated that the MERS-CoV reported in humans is genetically highly similar to the MERS-CoV reported in dromedary camels, which suggested that dromedary camels may be a potential reservoir for MERS-CoV and may facilitate the disease transmission to humans [47].

A sero-surveillance for the detection of HEV among pigs, cattle, and goats was conducted during January-March 2016. Indirect ELISA detected anti-HEV antibodies in 69 pigs while cattle and goat samples were found to be sero-negative [135].

During August 2015–February 2017, 142 sera samples were collected from 11 species of non-human primates. Sera samples from some of the domestic pet monkeys were also included in this study. Indirect ELISA using Ebola virus-specific antibody identified three different non-human primates having Ebola virus antibodies. The findings of this study suggested that contact or confrontation of the human population living in the region with non-human primates may put the human population at risk of disease contraction [136].

In a long-term study, carcasses from dead wild and domestic birds as well as cloacal and tracheal swabs from sick or suspected birds were collected during 2002–2015. A total of 101 NDV-confirmed samples were subjected to next-generation sequencing and Sanger sequencing, which generated 73 complete or near complete genomes of NDV and 38 partial sequences comprising the fusion gene. This study also identified genotypes and sub-genotypes among the NDV isolates, and interestingly, reported genotype IV and sub-genotype VIh of NDV for the first time from Africa [137]. Intriguingly, two isolates of a new genotype IV reported in this study from Oyo and Plateau provinces were originally reported from a chicken in 1973 and a duck in 1980 in Nigeria. The study suspected that this may be an extinct genotype of NDV. Another interesting finding was the first report of sub-genotype VIh from three bird samples. These three virus sequences clustered with the NDV sequences reported from a pigeon and quail between 2007 and 2013 along with viruses reported from Kenya and Argentina. The study also reported the spillover of NDV disease to new bird species [137].

The first strain of genotype XVII of NDV was reported from a free-roaming apparently healthy domestic duck in Kuru, Nigeria in 1992. The complete genome of 15,192 bp was obtained from next-generation sequencing using an MiSeq Illumina platform [138].

**Figure 10:**
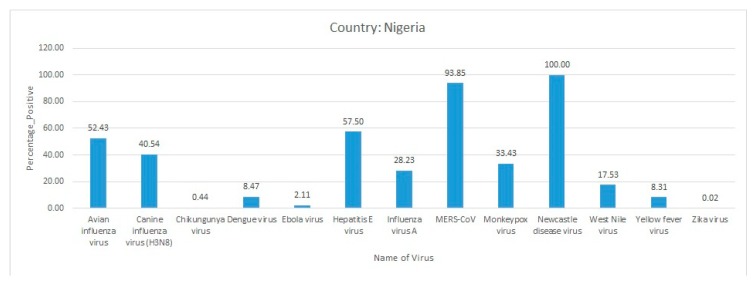
Frequency distribution of viral diseases in Nigeria in selected publications.

Newcastle disease, MERS, hepatitis E, avian influenza and monkeypox were among the most frequently reported disease outbreaks in Nigeria. IAV, canine influenza virus H3N8, WNV, YFV, Zika virus, Ebola virus, dengue virus, and chikungunya virus were among the less frequently reported virus diseases in Nigeria (Figure 10).

#### 3.2.14. Senegal

In total, 120 horses were sampled, and the sera was harvested for WNV detection among pastoral communities of Barkedji village in the Ferlo area of Senegal following a previous study, which reported the presence of *Culex* and *Aedes* mosquitoes in the Ferlo region [139]. Anti-IgM ELISA for the detection of WNV revealed that three horses had antibodies for the virus, thus a seroprevalence of the WNV in Senegalese horses was reported [140]. Interestingly, another study reported the prevalence of WNV in wild birds sampled at two different sites, one being at Barkedji village in the Ferlo region and the other at the Djoud’j National Park. Wild birds were trapped and sampled between 28 September and 7 October 2003 during certain hours of sunrise and sunset. The sera samples were harvested from 422 birds and tested with anti-WNV monoclonal antibody. Seven factors were considered while sampling the birds for data analysis, including the sampling site, migratory status, feeding behavior, resting site, type of nest, herd instinct level, and the affinity with urban areas. The ELISA test reported 23 WNV-positive birds out of the 422 tested, resulting in an overall seroprevalence of 5.5%. More precisely, all 23 WNV-positive birds belonged to seven bird families, out of which five bird families represented migratory birds [141]. The results obtained in this study suggested a probability of the role of migratory wild birds in introducing WNV disease in the country.

Following an influenza-like illness in the country during 2009–2010, a large-scale surveillance was conducted for the detection of influenza viruses. Nasopharyngeal and oropharyngeal swabs were collected from 2264 symptomatic patients; the extracted RNA samples were subjected to molecular detection of IAV and IBV. Only IAV-positive samples were further subjected to a second real-time RT-PCR assay to distinguish among the H1, H3, and A(H1N1)pdm09 viruses. Out of 2264 samples, 641 samples were found to be positive for IAV while 76 samples were positive for IBV. More precisely, 293 patients were found to be positive for H3N2 virus while 345 samples were positive for A(H1N1)pdm09 virus. Only three samples were positive for the IAV subtype H1N1. Interestingly, the phylogenetic analysis based on the HA1 sequences of five A(H1N1)pdm09 viruses retrieved in this study reflected a close association with the sequences reported from Cameroon, Ghana, and Cote d’Ivoire [95].

In total, 1434 individuals from nomadic pastoral communities from five north-eastern Senegalese districts were investigated for the seroprevalence of chikungunya virus during September–October 2014. Dried blood samples were obtained from the enrolled individuals and were subjected to serological examination using anti-CHIKV IgG antibodies using a method for bead-based IgG detection. The results showed that 39 nomadic pastoralist individuals were seropositive for chikungunya virus antibodies. This study suggested that there was no recent exposure to chikungunya virus in the nomadic pastoralist communities and therefore a low seroprevalence was reported in these nomadic pastoralists [142].

A Rift Valley fever case who tested positive for the disease made the authorities curious about a possible outbreak in the region; hence, neighbors, friends, family members, high school students, and other contacts of the index case were sampled for diagnostics of the RVFV. A total of 334 blood samples were tested for RVFV IgM and IgG as well as RNA. All the samples were negative for the disease except a 20-year-old housewife and a 32-year-old tradesman besides the case patient. The RVFV that was isolated from the patient appeared to be closely related to the virus that was reported from Mauritania in 2012 [143].

#### 3.2.15. Sierra Leone

The recent Ebola virus disease outbreak in the Western African country of Sierra Leone reported 10,746 total cases; almost one-third of the cases reported either a funeral or non-funeral (either household or work-related) exposure of Ebola virus [110]. In total, 158 patients reported that they might have contracted the disease due to exposure of the virus at the funeral they had attended in the recent past. A total of 1895 Ebola virus-positive cases in the country reported that they somehow became infected, but the infection did not occur due to a funeral of the deceased [110]. This study represented the high potential of zoonotic transmission of the Ebola virus.

A new sequence-based study revealed the transmission chains of Ebola virus in Sierra Leone. This study included 855 samples collected from patients admitted to the Ebola care centers in different districts in the country during December 2014 and September 2015. The total nucleic acid extracted from these samples was screened for Ebola virus RNA using real-time PCR. The samples were divided into two pools: The first pool was screened using 73 Ebola virus-specific primer-pairs in the presence of five human housekeeping genes as a control. Samples in a second pool were screened using 72 Ebola virus-specific primer-pairs and the housekeeping genes as controls. Ion Torrent Hi-Q sequencing generated 554 Ebola virus genome sequences from the positive samples [144]. Blood, buccal swab, breast milk, and semen samples were successfully used to recover the Ebola virus genome sequences greater than 10 kb in this study. Based on the genome sequences, the study reported that at least nine different lineages of Ebola virus circulated in this outbreak. Interestingly, eight virus lineages appeared to be variants of the Sierra Leone 3 lineage, which was first reported in June 2014, while one cluster appeared to have derived from the GUI-1 lineage, which was originally reported in Guinea. This study reported human-to-human transmission of Ebola virus through birth, contact, and breast feeding [144].

From August 2015 to January 2016, eight human Ebola virus cases were reported from Sierra Leone [144,145]. In August-September 2015, a deceased woman and her surviving daughter both were found to be positive for Ebola virus and it was considered a human-to-human transmission either through close contact or through body fluids [146]. The Ebola virus genome recovered from the deceased woman was quite similar to that of a male survivor, who was believed to be involved in sexual intercourse with the deceased woman [146]. In January 2016, another deceased woman was found to be positive for the Ebola virus disease; however, the disease transmission to this woman could not be linked to a known survivor [146].

Another surveillance study reported 232 Ebola virus cases in Sierra Leone during the 2013–2015 outbreak based on genome sequences. This report also suggested human-to-human transmission of the virus [147].

Human cases of MPXV disease have also been reported from Sierra Leone. The first human case of MPXV disease was reported in the year 1972 when six suspected cases were investigated but only one was found to be positive for the disease [96]. During 2010–2018, at least two confirmed cases of MPXV disease appeared [97]. The possibilities behind the MPXV disease were thought to be either higher human–wildlife conflict for bushmeat in the country or human-to-human transmission through body fluids [97].

#### 3.2.16. Togo

A serological survey reported the incidence of influenza D virus (IDV) in small ruminants in Togo [49]. A total of 541 samples from cattle (*n* = 201), sheep (*n* = 135), and goats (*n* = 205) were collected from Togo during 2009 to 2015. The HI and microneutralization (MN) assays determined that 10.4% of cattle, 2.2% of sheep, and 1.4% of goat samples were serologically positive for IDV-specific antibodies [49]. There was no ruminant-related import or export reported from Togo. The findings of this study suggested that IDV circulated among ruminant species in Togo [49].

During the years 2009–2010, 2454 oropharyngeal and cloacal swab samples were collected from birds. All the samples were subjected to RT-PCR-based IAV diagnostics, which revealed that none of the samples were positive for IAV infection. Additionally, 436 sera samples were also collected from birds and subjected to ELISA as well as HI assays using IAV-specific antibodies, but all were negative for IAV [91].

**Figure 11:**
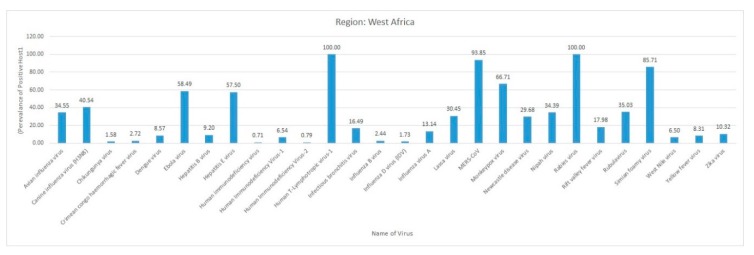
Distribution of virus diseases in West Africa in selected publications.

In another investigation, 325 swine nasal swab samples were collected from an abattoir in Lome during October 2012–January 2014. Samples were pooled for five samples per tube to reduce the cost of diagnostics, resulting in 65 pools. The pigs appeared asymptomatic at the time of sampling. The extracted viral RNA was subjected to specific one-step real-time RT-PCR for the detection of IAV in the samples: Eight pools were found to be positive. Further, a subtyping assay based on real-time RT-PCR for the HA gene identified that all the positive pools had A(H1N1)pdm09 virus sequences. Virus isolation in MDCK cells was attempted, and therefore, one complete genome was retrieved. The phylogenetic analysis revealed that the A(H1N1)pdm09 virus retrieved in this study was closely related to human A(H1N1)pdm09 viruses that had circulated in the region since 2010. Therefore, this study suggested a possible transmission of the virus from humans to swine [33]. Overall, Ebola virus, HEV, HTLV-1, MERS-CoV, MPXV, rabies virus, and SFV have been widely reported from West African countries, with a large number of positive cases. However, cases of YFV, IAV, avian influenza virus, Zika virus, dengue virus, and chikungunya virus were also reported from West Africa in different host species (Figure 11).

### 3.3. Central Africa

Countries in Central Africa include Angola, Burundi, Cameroon, Central African Republic, Chad, Democratic Republic of Congo, Equatorial Guinea, Gabon, Republic of Congo, Rwanda, Sao Tome, and Principe, as well as South Sudan.

#### 3.3.1. Angola

In a study, residual sera samples from 6839 patients who visited clinics in Central Luanda with typical dengue fever symptoms between 1 January 2016 and 15 May 2018 were collected for this study to investigate the seroprevalence of dengue virus. Screening for dengue virus was done for 153 randomly selected sera samples, out of which 80 samples were found to be positive for DENV-NS1 protein. The first confirmed case of dengue virus was detected in May 2017. All 153 randomly selected sera samples were further tested for dengue virus RNA using real-time quantitative RT-PCR, which successfully detected one positive sample for dengue virus serotype-2 (DENV-2). This case reported traveling to a nearby island during 24 December 2017 through to 2 January 2018. Attempts were made to amplify the entire coding region of this DENV-2 isolate for sequencing. The phylogenetic analysis clearly determined that the dengue virus isolate retrieved from the man in Luanda was the DENV-2 serotype and formed a monophyletic clade with dengue virus genomes reported earlier from east African countries [148].

#### 3.3.2. Burundi

A study investigated the genotypes and subtypes of hepatitis C virus (HCV) in the central African country of Burundi between January and May 2013. In this investigation, 179 serologically HCV positive patients were enrolled to further study the circulating genotypes and subtypes of HCV. TaqMan real-time RT-PCR was used to detect the HCV RNA in the samples; HCV genotyping and subtyping was also done. The results found that the highest number of patients (166 patients or 92.7%) were infected with the HCV-4 genotype. Ten patients were identified to carry genotype-1 (HCV-1) while three patients were infected with genotype-3 (HCV-3). Subtyping was successfully done for 51 HCV-4 cases, which identified that the 4h subtype represented the highest frequency (49.1%) of infection followed by the 4e (21.6%) and 4k (3.9%) subtypes. Therefore, this study reported that HCV-4 was the most common genotype of HCV in Burundi along with the 4h and 4e subtypes, which were most commonly present [149].

#### 3.3.3. Cameroon

Stool samples from chimpanzees, gorillas, and orangutans, were collected from different locations in Cameroon to investigate the shedding of adenoviruses. Samples were collected from 43 non-human primates living in their natural habitat. A conserved sequence of the DNA polymerase gene of the adenovirus genome was amplified from 18 stool samples with nested PCR. However, attempts at virus isolation were unsuccessful, but this study reported the prevalence of adenovirus in non-human primates living in the natural habitat [150].

The A(H1N1)pdm09 virus was first reported in domestic Cameroonian swine during 2009–2010. Following a mild respiratory disease among Cameroonian swine herds, 104 nasal and 98 blood samples were taken from 11 domestic swine herds in several villages. The extracted RNA from swine nasal swabs was subjected to real-time PCR for the IAV matrix gene. Matrix gene-positive swine samples were further subjected to A(H1N1)pdm09 virus-specific real-time RT-PCR followed by virus isolation. Additionally, HI assays were conducted on the sera samples to detect IAV-specific antibodies. The real-time PCR detected two positive swine nasal swabs, which were successfully subtyped as A(H1N1)pdm09 virus. Interestingly, the youngest infected swine was found to be four months of age, which indicated that the A(H1N1)pdm09 virus was in circulation in this swine herd within the last four months of the sampling [32].

Another investigation was conducted to investigate the spillover of A(H1N1)pdm09 virus. The study reported active infection of A(H1N1)pdm09 virus in two swine samples. Further, 325 swine nasal swab and sera samples were collected from 12 different sites during December 2009–August 2012. Cloacal swab samples from 582 domestic poultry birds, including chickens, ducks, geese, and turkeys, as well as 1479 wild birds were collected. Samples were diagnosed using real-time RT-PCR and ELISA assays. The positive sera samples were further tested with the HI assay for confirmation. Real-time RT-PCR detected active A(H1N1)pdm09 virus infection in one swine nasal swab sample from each of the two study sites. Interestingly, sera samples collected from all domestic poultry were found to be negative for IAV. This study suggested that the free-roaming nature of pigs may put them at risk of acquiring the disease because in this manner, they may come in frequent contact with other animals, including ducks and wild birds, as well as humans [151]. The findings of the study suggested the role of domestic ducks and wild Columbiformes as the probable intermediaries for IAV transmission and spillover [151].

Apart from this, a highly pathogenic IAV H5N8 subtype has recently been reported from Cameroon. The IAV H5N8 subtype was detected in duck, chicken, guinea fowl, and pigeon populations [152]. The findings of this study suspected the role of wild birds in the transmission of IAV among different locations given that the IAV H5N8 subtype was simultaneously detected in poultry and wild birds in Uganda and Tunisia [152].

A 2005–2012 seroprevalence study consisting of 137 individuals of Pygmies, the community of hunter-gatherers that has lived in the dense rain forests of Cameroon for centuries, resulted in 17 RVFV-infected and 6 CCHFV-infected individuals while two individuals were found to be positive for both viruses [153]. This study suggested that Pygmies people, being in frequent and close contact with non-human primates, were prone to zoonotic virus diseases [153].

In another study, fecal samples from 27 chimpanzees and 27 gorillas, collected from forests not disturbed by human activities, were screened for the presence of enteroviruses. Enteroviruses are members of the family *Picornaviridae* and are known to cause infections in humans and Old-World monkey species. Four of the chimpanzee samples were found to be positive for enteroviruses based on highly conserved 5’ UTR nucleotide sequences [154]. This study provided information on cross-species transmission of enteroviruses [154].

Another study provided evidence of zoonotic transmission of Nipah virus. A seroprevalence study showed that people involved in the butchering of bats for bushmeat were most likely to contract the infection [155].

Hepatitis E virus (HEV) is also widespread among pigs. A serological study found that 70 out of 162 pigs sampled were positive for HEV [156]. The study suggested the pigs were the animal reservoir of HEV in Cameroon [156].

The human population in Cameroon is at high risk of zoonotic virus infections. In a study, Western blot assay revealed that a blood donor was positive for dual infections with HIV-1 and SFV [157]. This study suggested the zoonotic transmission of SFV from non-human primates to humans.

In another interesting study, it was observed that HIV-1 infection in Pygmy hunter-gatherers in Cameroon was due to contact with the Bantu community [1]. Bantu and Pygmy are forest-based tribal communities, which usually remain in close contact with non-human primates and other wild animals. In this study, three Pygmies and seven Bantu individuals were positive for HIV-1 in PCR-based detection, which indicated the zoonotic potential of HIV-1 among tribal communities living in forests [1].

In a large-scale seroprevalence study, 3955 plasma samples were collected from individuals in Cameroon to screen for HIV-1 infection. As a result, 191 individuals were found to be seropositive for HIV-1 infection. Interestingly, these people were in frequent contact with non-human primates either through hunting activities or butchering for bushmeat [158], hence their behavior puts them at high risk of zoonosis for HIV-1.

The emergence of a new retrovirus named as human T lymphotropic virus (HTLV) has recently been reported from communities in Cameroon [159]. In a sera-based surveillance, 29 individuals from different ethnic groups out of 408 sampled were found to be positive for HTLV infections. Two variants of HTLV were reported from this population: HTLV-1 and HTLV-3 [159]. A large fraction of the study participants reported exposure to non-human primates either through hunting or butchering or having them as pets. Hence, exposure to non-human primates evidently puts the human population at high risk for zoonotic transfer of virus diseases, as well as facilitating the emergence of a new species of viruses.

An investigation among bushmeat hunters reported new strains of HTLV in their community: HTLV-3 and HTLV-4 [160]. It was also suggested that contact with the blood and body fluids of non-human primates, mainly gorillas, chimpanzees, and monkeys, increased the risk of zoonotic transmission of these viruses to the hunter gatherers. This study also suggested that STLV-3 and STLV-4, through cross-species transmission, became endemic in the human population [160].

Another study documented HTLV-1, HTLV-2, and SFV infections in pygmies. In this study, 5 pygmies out of 35 individuals were found to be infected with HTLV-1, nine were found to be positive for HTLV-2, while one individual had dual infections with HTLV-1 and HTLV-2. Five people were positive for SFV infection while one person was positive for primate T lymphotropic virus-3 (PTLV-3) infection [161]. This study again suggested the contact of these communities with non-human primates was the source of infections. These findings were backed by an earlier study, which suggested that contact with non-human primates through hunting or butchering triggers the zoonosis [162]. This was more evidence of zoonotic transmission of SFV from non-human primates to the human population [162].

Another surveillance study included two groups of the population in Cameroon; the first group included 1321 individuals from the general population while the second group consisted of 198 individuals who were either bitten or scratched by non-human primates in the past [163]. While only two people were infected with SFV in the general population, a larger fraction of individuals (37/198) who were either bitten or scratched by non-human primates were found to be positive for SFV [163]. In another seroprevalence study, 179 individuals who were exposed to non-human primates through hunting, butchering, or other activities were found to be positive for SFV from a population of 1099 exposed participants, indicating the zoonotic potential of SFV and transmission from non-human primates to humans [164].

Simian T-cell leukemia virus (STLV) strains, STLV-1 and STLV-3, have also been reported in gorillas and monkeys from Cameroon [165]. The prevalence of these viruses among non-human primates is a potential threat to the local population for zoonosis.

Monkeypox virus (MPXV) is another emerging zoonotic virus in Africa, including Cameroon. MPXV is a member of the *Poxviridae* family, which represents human pathogens. In a recent report, until 2018, four confirmed cases of monkeypox have been reported from Cameroon [97]. The zoonotic potential and severity of monkeypox puts the human population at risk in the country.

Liver tissue samples that were collected from pigs during February–March 2012 for HEV surveillance were used for the detection of the prevalence of porcine hokovirus. A total of 282 liver tissue samples, which were divided into 94 pools, were subjected to molecular diagnostics using quantitative real-time PCR. In total, 65 of the 94 pools were found to be positive for porcine hokovirus infection. All the positive pools were then tested individually. As a result, 128 individual samples were found to be positive for porcine hokovirus infection in pigs [166].

In another surveillance for influenza virus infections among the human population during 1999–2014, 259 human infections of the H3N2 subtype of IAV and 102 cases of IBV infections were reported from Cameroon [52].

An avian influenza H5N1 outbreak hit poultry during May- June 2016. To investigate the status of poultry flocks, 147 trachea and cloacal swab samples were collected from 7 regions encompassing 19 different sites. Additionally, biopsies of the internal organs were collected from the dead post-mortem poultry birds. RNA was extracted from the poultry samples and were subjected to real-time RT-PCR for the detection of the M- gene. Further, IAV-positive samples were tested for the H5 and H7 subtypes. The H5-positive samples were also tested for the N1 gene for confirmation of the subtype. The results revealed that out of 147 poultry samples, 58 were positive for IAV. Subtyping identified 34 poultry samples that were positive for H5N1 virus. Simultaneously, blood samples along with oropharyngeal and nasopharyngeal swabs were collected from poultry workers or poultry-exposed individuals who worked either at the poultry farm or in a live poultry market for investigation. Only 15 human samples were found to be positive for IAV, which were successfully subtyped into 11 isolates of H3N2 virus, while four isolates could not be subtyped. Seroconversion was detected only in two poultry workers [167].

Sera samples were collected from 29 swine herds during May–June 2011. A total of 197 sera samples from 29 swine herds were collected from the majority of healthy or clinically normal pigs while only a small percentage of pigs had diarrhea. Sera samples were tested by a virus microneutralization assay using MDCK cells given its higher sensitivity over the HI test. The results found that nine swine herds were positive for A(H1N1)pdm09 virus while the nine other herds were positive for H1N1 virus. This study suggested that IAV was in circulation during 2011 [129].

**Figure 12:**
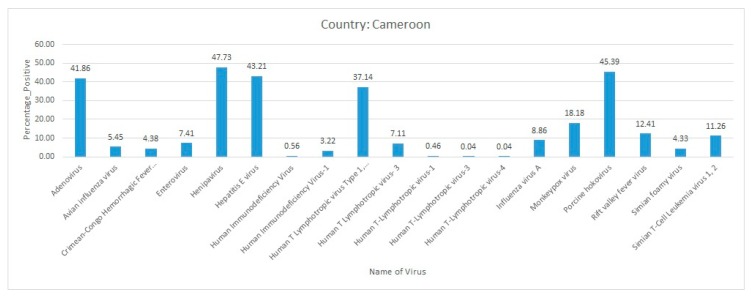
Distribution of virus diseases in Cameroon in selected publications.

Overall, the adenovirus, HEV, henipavirus, HTLV, and porcine hokovirus were the most frequently reported virus diseases in Cameroon. The MPXV, IAV, RVFV, STLV, HIV-1, CCHFV, enterovirus, and avian influenza virus diseases were comparatively less frequently reported in Cameroon (Figure 12).

#### 3.3.4. Central African Republic

In September 2018, three members of a Pygmy family in the Central African Republic, including a mother and her two daughters, were reported to have contracted MPXV infection, with the index case female exhibiting symptoms of a maculopapular rash and lesions on the palms and soles of the hands and feet, respectively. In the first week of October 2018, an investigation was launched and learned that the index case female recently butchered three small mammals a few days before the onset of the rash and lesions. A few days later, two more family members of the index case female became symptomatic for the MPXV disease, which was confirmed by testing their samples with PCR. In the last week of October, another relative of this index case female was reported to have contracted the disease. In this investigation, it was observed that the disease spread among the index case female’s family members and relatives through three waves of infection and hence was considered as an intrafamilial transmission of MPXV disease [168].

#### 3.3.5. Chad

An ELISA-based seroprevalence study of RVFV disease was conducted among ruminants in Chad. The study area included nine villages and camps located on the south-eastern shore of Lake Chad. Sera samples from 715 cattle, 144 goats, and 65 sheep were collected and processed for RVFV diagnostics using ELISA. A total of 270 cattle, 27 goat, and 7 sheep samples were positive for the RVFV antibodies. The study speculated two possible factors behind the higher seroprevalence of cattle than goats and sheep in this investigation: The longer life span of cattle, which makes them vulnerable to the acquisition of disease compared to small ruminants, and the exposure of cattle to Lake Chad while goat and sheep are mostly restricted to a limited area [169].

#### 3.3.6. Democratic Republic of Congo

An HPAIV H5N8 subtype was reported from ducks and a chicken in 2017. A total of 11 out of 21 sampled ducks, were found to be positive for the H5 subtype of avian influenza virus using reverse-transcription PCR [170]. This study assumed possible contraction of the disease from wild birds.

The first outbreak of Ebola virus disease reported 319 cases in 1976–1977. Later, 315 cases were reported in 1995, and lately, 296 cases were reported in 2007–2008 [109]. In another study, an Ebola virus outbreak was reported among the human population due to direct exposure to fruit bats [71]. Additionally, there was a spread of the virus within a household where another family member of a 55-year-old lady became infected and developed similar symptoms of viral hemorrhagic fever. This study reported bat-to-human and then human-to-human transmission of the Ebola virus [71].

In a surveillance study, one commercial sex worker and one blood donor were found to be positive for HIV-1 and SFV infections [157]. It was suspected that the butchering and hunting of non-human primates was a potential risk factor for the transmission of the disease to these cases.

Twenty-four stool samples from non-human primates living in forest areas were collected. DNA was extracted from all the samples and subjected to nested PCR for amplification of the DNA polymerase gene of adenovirus. The goal of this investigation was to undertake a study of the assessment of adenovirus shedding in the stool of non-human primates. A total of nine stool samples from chimpanzees and gorillas were found to shed adenovirus in their stool. Due to certain technical limitations, the virus isolation attempts were not successful. [150].

In another study, a nine-month-old child was the first reported case of MPXV disease in Bokenda village in 1970 who was believed to be infected with smallpox, but the virus isolation confirmed it to be MPXV infection. His family had been eating monkeys occasionally as a delicacy [97]. Later, a widespread outbreak of human MPXV was reported during 1996–1997 in 12 villages. The virus was successfully isolated from seven patients along with the amplification of DNA sequences from specimens obtained from the skin lesions of human subjects participating in the study [171]. Interestingly, various species of squirrels, elephant shrew, and Gambian rat captured in this area along with domestic pig were found to be positive for the MPXV infections [171]. The participants in this study confirmed eating the wild animals. The study concluded that the lifestyle and exposure to wild animals put the human population in the area at risk as far as transmission of the disease from animals to humans was concerned.

In a hospital-based transmission of MPXV disease, 11 confirmed or probable cases were reported [172]. Interestingly, the case 1 was a child who owned a pet monkey at home, but there was no report of any sort of illness in the recent past in this monkey. Other cases were either residents in the hospital or were associated with the hospital staff. Some common symptoms included fever, rash, and eye disorders. The death of one patient was also reported, who most probably died of infection with MPXV [172].

In the period of February to August 2001, seven outbreaks of suspected MPXV disease were reported [173]. Several patients had similar symptoms, which suggested a common source of infection, which was thought to be a dead monkey from the forest that ended up as a meal in a family. It was also thought that these people might have spread the disease among others in their contacts. The most common symptoms in the deceased were conjunctivitis, generalized eruptions, and pulmonary failure while others with less severe disease experienced pustular lesions on different body parts along with enlarged lymph nodes [173]. The laboratory investigation revealed that the first two outbreaks, which resulted in 16 cases with four deaths, were primarily due to the MPXV infection. Two outbreaks were due to cocirculation of MPXV and varicella-zoster virus (VZV), which claimed one death out of seven cases, while two other outbreaks were found to originate from chicken pox infection among six people. The seventh outbreak was mainly due to MPXV infection while two cases reported orthopox virus infection as well; the outbreak primarily affected young children [173].

The first case of SFV infection was reported in the year 1985. Human contact with non-human primates was suspected to be the source of infection [157]. The second case of SFV was reported to originate from mandrills (*Mandrillus sphinx*). In this case, hunting and butchering of mandrills was suspected as the primary source of zoonosis [157]. Three SFV infections were reported from people involved in the butchering or preparation of either Red Tail or Angolan Colobus monkey meat for eating [174]. Interestingly, a very high concentration of SFV virion particles (104 to 109 RNA copies/104 cells equivalent) were reported to be present in the saliva of non-human primates that were carrying infection. Since the oral mucosa tissues of Macaques and African green monkeys are sites of virus replication, biting, licking, or saliva-based transmission of SFV was considered to be the primary source of zoonosis [175,176,177].

A widespread infection of SFV was reported from rural areas of the country in the year 2007. Sixteen patients were confirmed for SFV infection with Western blot analysis, out of which 14 patients were further confirmed with SFV sequences [178]. Phylogenetic analysis using the sequences obtained from non-human primates and humans confirmed the origin of the disease was from the non-human primates [178]. The dependence of the rural population of the country on bushmeat for food and income tends to be the driving force behind the emerging trend of zoonosis in rural areas of the country.

In a sero-surveillance study assessing the seroprevalence of RVFV in cattle, blood was collected from the coccygeal vein of 677 cattle across 17 villages in Kwilu province during April 2017. Sera was tested against anti-RVFV antibodies using a nucleocapsid protein-based indirect immunofluorescence assay. The positive and intermediate cases were re-tested with ELISA for confirmation, which identified 38 cattle having anti-RVFV antibodies. The positive results were found among the samples collected across the territories, which indicated that the RVFV disease was prevalent among cattle across the Kwilu province [179].

#### 3.3.7. Equatorial Guinea

Blood donors from the urban areas of Malabo city and adjacent rural areas as well as parts of Bioko island were screened for the infection of HBV, HCV, and HIV at Malabo Regional Hospital blood bank between January 2011 and April 2013. A total of 2937 blood samples were first screened with colloidal gold immunochromatographic assay test strips. The positive samples were further confirmed for positivity with specific ELISA tests for these viruses. The results found that 230 (7.83%) blood donors were positive for HIV while 294 (10.01%) blood donors were positive for HBV infection. The HCV seroprevalence was determined to be 3.71%, with 109 positive cases. Interestingly, 29 blood donors were found to be co-infected with HIV and HBV while 8 donors were co-infected with HIV and HCV, as well as HBV and HCV each. This study, for the first time, reported the seroprevalence of HIV, HBV, and HCV in blood donors from Bioko island [180].

#### 3.3.8. Gabon

The tiger mosquito *Aedes albopictus* was identified as the vector of chikungunya virus during the 2007 outbreak of the disease in Gabon. People with symptoms of fever, arthralgia, and/or rash were subjected to the investigation. A total of 24 symptomatic individuals were bled for the detection of a panel of viruses, including chikungunya virus, Zika virus, WNV, dengue virus, YFV, and RVFV, using a specific TaqMan probe in real-time RT-PCR reactions. In total, 12 samples were found to be positive for the chikungunya virus infection [181]. To identify the potential vector responsible for the outbreak, 640 mosquitoes were collected, which were further distinguished according to their species. Three pools of individual mosquito species were made: The first species had 627 mosquitoes that were grouped into 45 pools of *Aedes albopictus*. The second had 12 *Aedes aegypti* mosquitoes grouped into 6 pools, and the third pool had only one mosquito of the species *Aedes simpsoni*. Intriguingly, 32 pools (71%) of *Aedes albopictus* mosquitoes were found to be positive for chikungunya virus using the same TaqMan probe in real-time RT-PCR assay [181]. Only one pool of *Aedes aegypti* was positive for the chikungunya virus while the *Aedes simpsoni* mosquito was negative for the virus infection [181]. This study established the *Aedes albopictus* mosquito as the potential vector for the chikungunya virus’ dissemination.

An Ebola virus outbreak was reported from non-human primates in Gabon. A total of 210 confirmed infections were reported during the period of 1994–2002 [71]. The investigation reported that human-to-human contact was the most likely cause of the outbreak; however, a link between bats and humans was also suspected [71]. In another investigation, 14 human subjects were found to be positive for Ebola virus infection in the country [2]. On the other hand, populations of gorillas, chimpanzees, and duiker antelope were also found to be positive for Ebola virus infection [2,3]. It was believed that fighting among gorillas and migration would be the main causes behind the spread of disease in animals. Animal–human conflict and exposure to these wild animals would be responsible for the introduction of Ebola virus into the human population. Human-to-human contact would be the secondary factor of disease dissemination.

At least 10 confirmed cases of MPXV disease were reported in 1987 [182,183]. Two driving forces behind the outbreak were recognized: Increased exposure to the reservoir species of MPXV and frequent human-to-human transmission of the disease [97].

In an attempt to characterize the cross-species transmission of SFV to humans, 497 samples from non-human primates comprising blood and tissues and 78 blood samples of humans, especially those who recalled an encounter with non-human primates in the recent past, were collected. These people either received a bite or scratch from non-human primates. A total of 31 out of 286 blood samples from non-human primates had antibodies against SFV [184]. A total of 19 humans who were mostly hunters were seropositive for the SFV while 15 were found to be positive with PCR [184]. This study provided evidence of cross-species transmission of SFV from non-human primates to humans who were exposed to the wild.

In another study, 84 mandrills were investigated for the infection of SFV. In total, 70 captive and nine wild-caught mandrills were found to be seropositive for SFV [185]. A group of 20 human individuals who worked closely with the mandrills were also subjected to an investigation to assess the inter-species transmission of the disease. In total, two out of 20 human subjects were positive for SFV infection [185]. This was another study that proved cross-species transmission of SFV to humans.

Another investigation studied the status of SFV prevalence in non-human primates and its potential for cross-species transmission to an exposed human population. In total, 273 blood samples from captive species of non-human primates and 211 tissue samples from animals sold as bushmeat were collected. A total of 9.8% of the captive animals and 4.7% of the bushmeat samples were found to be positive for SFV with PCR analysis [186]. Another group consisted of 78 human individuals who were involved in hunting or playing with non-human primates. These people were either bitten or scratched by the animals at various occasions. The study identified that 19 human individuals were seropositive for the SFV infection while the PCR could only amplify the SFV integrase fragment in 15 DNA samples [186]. This study also provided evidence of the cross-species transmission of SFV from non-human primates to humans.

A total of 48 samples from different species of non-human primates, including grey-cheeked mangabeys, mustached monkeys, De Brazza’s monkeys, greater spot-nosed monkeys, crested mona monkeys, mandrill, and red-capped mangabeys were collected. Seven samples were found to be positive for HTLV-1 while one sample was positive for both HTLV-1 and HTLV-2. Thirteen samples were positive for STLV-1 and three samples were positive for STLV-3, while one sample was positive for both STLV-1 and STLV-3 [187]. Since there were reports of frequent encounters of the local human population with these non-human primates, the local population is always at high risk of cross-species transmission of the virus.

#### 3.3.9. Republic of Congo

Ebola virus disease outbreaks have been reported in the Republic of Congo during 2001 to 2005. During this period, a total of 249 cases of Ebola virus disease were reported, with an alarmingly high mortality rate: 211 patients (84.74%) died [71]. An investigation revealed that the disease outbreaks originated from wild reservoirs, basically from non-human primates, and crossed the species barrier to infect exposed human populations [71].

Monkeypox was also reported in the country during 2000–2009. Three confirmed cases were detected out of 12 suspected cases. Later, the frequency of the disease increased, and 98 suspected cases were reported during 2010–2018, with 9 confirmed patients [97]. This indicated an increased rate of transmission of the disease among humans in recent times. As the reports have suggested, there may be two possibilities behind increased rate of disease incidence: Higher human–wildlife conflict for bushmeat in the region and/or a higher human-to-human transmission rate through body fluids [97].

In a sero-surveillance during March–July 2011, sera samples were collected from 386 healthy urban and rural blood donors. A serum neutralization test for Zika virus detection identified a low seroprevalence in the country, with only seven healthy individuals (1.8%) having Zika virus antibodies. This investigation suggested a limited circulation of Zika virus in the Republic of Congo [188].

#### 3.3.10. Rwanda

After an influx of abortion cases among cattle in Rwanda during December 2012–March 2013, an epidemiological surveillance was conducted across six districts. Sera samples were collected from 595 cattle and subjected to ELISA using RVFV-specific antibodies. In total, 100 sera were found to be positive for the RVFV antibodies. The study suggested that the predominance of mosquitoes, which might serve as a vector for the disease, may be an important factor behind the prevalence of RVFV in the Nile basin [189].

The first case of IAV disease was reported in October 2009 from a Rwandan woman of 42 years of age who came back to Kigali after completing a short US visit. She had an influenza-like illness; later, the same symptoms were reported in two of her children on 6 October 2009. On 8 October 2009, samples from the index case woman were found to be positive for A(H1N1)pdm09 virus through real-time RT-PCR. Immediately after this report, the authorities started tracing the contacts of the index case woman. Her friends, neighbors, the office where she worked, the hospital where she got treated, as well as the school where her children went for education were among the most probable spots for infection, hence these were investigated. All the suspected cases were sampled for nasopharyngeal or oropharyngeal swabs for diagnostics of the A(H1N1)pdm09 virus. Interestingly, a foreigner living in Gisenyi city, which is located away from Kigali, developed influenza-like symptoms after his arrival from the USA on 27 October 2009. His samples came up positive for A(H1N1)pdm09 virus on 30 October 2009. After some time, in November 2011, an A(H1N1)pdm09 virus infection was detected in two foreign nationals who arrived in Rwanda from Europe. During this influenza pandemic period in Rwanda, a large number of community cases were reported and tested positive, which confirmed the widespread presence of the disease in the country.

Between October 2009 and May 2010, a total of 2045 nasopharyngeal and oropharyngeal human samples were screened for influenza viruses. Out of these, 510 IAV- and 22 IBV-positive samples were found. Among the IAV-positive samples, 494 were positive for H1N1 subtype, while 15 cases were A(H1N1)pdm09 and only one case was positive for H3N2 subtype. Most of the cases exhibited influenza-like illness while only a few had severe acute respiratory illness [190].

#### 3.3.11. Sao Tome and Principe

During 2003–2004, a total of 78 pregnant women were sampled and tested for the seroprevalence of dengue virus. Sera samples were first screened through the immunofluorescence assay, which determined 31 sera (39.74%) as being positive for dengue virus antibodies. Further, sera were tested with indirect ELISA using dengue E IgG antibodies and NS1 IgG antibodies, which revealed that 53 sera (67.95%) were positive for dengue E IgG while 38 sera (48.72%) samples were positive for dengue NS1 IgG. Only those sera samples that were found to be positive with all three tests were considered positive for dengue virus IgG while the others were considered negative. This resulted in, overall, 28 sera samples (35.90%) that were positive for dengue virus IgG. Interestingly, out of these 28 IgG-positive samples, only one serum sample was positive for IgM-capture-ELISA, which suggested that there was only one recently acquired dengue virus infection in the study population while the indirect IgG ELISA results indicated that most of the dengue virus infections observed in this study occurred in the past [191].

#### 3.3.12. South Sudan

A case of an eight-month-old child was the first reported case of MPXV disease from South Sudan in 2005. The child developed mild symptoms, initially including fever and cough among other symptoms, which suddenly became severe with the onset of a pustular rash and lesions in different body parts within the next few hours. Investigations of the specimens from this child were conducted at the Institut Pasteur in France, where the child was confirmed as positive for MPXV disease. Interestingly, the mother of this child developed similar symptoms during this time and was also confirmed as positive for the disease [192]. Following these findings, 49 additional humans in the region with the disease symptoms were sampled in three different categories: 30 were considered suspected cases, nine were considered probable cases, and ten were considered confirmed cases based on the symptoms and the severity of the disease. The ELISA and PCR-based laboratory tests confirmed that ten samples were positive for MPXV disease and the 942-bp sequence retrieved from the HA gene indicated close similarity with the Congo Basin strain of MPXV [192]. It was concluded that either the flooding situation in the region brought mammals present in the area into contact with the local human population or it might have originated from the wild reservoir and might have spread through the chains of human-to-human transmission [192].

**Figure 13:**
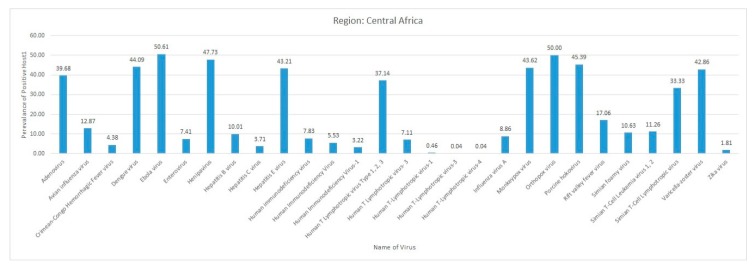
Distribution of virus diseases in central Africa in selected publications.

Three human cases, which developed a pustular rash, were found to be positive for the Varicella-zoster virus infection in January 2006, but the source of infection was unknown because the investigation was seized due to a lack of resources [192]. To summarize, various virus diseases have been reported in central Africa, including some of the most important diseases like Ebola virus, CCHFV, avian influenza virus, IAV, dengue virus, HIV, MPXV, and Zika virus, among others (Figure 13).

### 3.4. North Africa

North African countries are Algeria, Egypt, Morocco, Sudan, and Tunisia.

#### 3.4.1. Algeria

In late 2017, a high mortality was observed in poultry flocks in Algeria. During October–November 2017, three poultry flocks were taken under investigation in the Fouka region. Congestion was observed in the visceral organs, including the trachea, lungs, pancreas, liver, and kidneys, of dead birds. Fifteen HA gene sequences of avian influenza virus H9N2 subtype were retrieved from the tissues of dead poultry. Phylogenetic analysis showed that these sequences were closely related to the H9N2 virus sequences reported recently in Morocco and Burkina Faso [193].

In another study, 56 ticks (*Hyalomma aegyptium)* were collected to determine their biological and epidemiological role in Laghouat province during 2009–2010. Ticks were investigated for CCHFV infection using nested RT-PCR, which detected that 16 ticks were positive for CCHFV infection. A total of 15 amplicons were sequenced, which identified the AP92 strain of CCHFV in *H. aegyptium* ticks collected from tortoises [194].

A respiratory disease outbreak hit the horse population in Tiaret during May 2011. The non-vaccinated horses exhibited respiratory disease symptoms, including fever, nasal discharge, dry cough, and mild weakness. Out of 325 horses under epidemiological investigation, only 12 horses were sampled for nasal swabs, having severe respiratory disease. RNA was subjected to real-time RT-PCR for the detection of equine influenza virus. The RNA of the positive samples was then transcribed to cDNA and PCR was conducted to amplify the HA and NA genes. The amplicons were sequenced, and the assembled sequences were compared to the equine influenza virus sequences reported on GenBank, which identified that the sickness of the horses was due to infection with IAV H3N8 subtype. In total, 11 out of the 12 horses sampled were positive for the H3N8 infection. The phylogenetic analysis indicated that the IAV H3N8 subtype retrieved from these horses was in circulation in European horses during 2010–2012 [195].

#### 3.4.2. Egypt

In a three-year period starting from March 2006 to March 2009, a total of 6355 of suspected avian influenza H5N1 cases were reported in Egypt while 63 people were confirmed to have infections, out of which 24 died because of complications [196]. It was believed that the disease first appeared in domestic poultry, which quickly affected backyard chickens and commercial poultry, and hence became zoonotic. However, there was no human-to-human transmission reported, but interestingly, three family clusters were reported. The first family cluster, which comprised of two siblings, was reported in March 2006. The second cluster, comprised of three family members, was reported in December 2006. The third cluster, also comprised of two siblings, was reported in March 2007. However, in the first two clusters, the individuals shared a common exposure, of course, to the infected poultry and as a result became sick, while the siblings in the third cluster had two separate exposure incidences to the birds, which were most likely infected [196]. The common symptoms reported upon illness were fever, cough, sore throat, shortness of breath, muscle or joint pain, and headache. There were 19 cases with acute respiratory distress syndrome, out of which 18 died. Six other patients died, increasing the toll to 24 (38%). The virus was successfully isolated from 34 case patients [196]. Sequencing of the HA and NA genes confirmed that the virus belonged to clade 2.2. The isolates of the virus were found to be related to the ones reported from Europe and the Middle East [197]. Domestic poultry infected with H5N1 were the likely source of infection among humans as many of the cases were either in direct contact with backyard poultry or were involved in the production and distribution of flocks [196].

Another study reported the zoonotic transmission of H5N1 subtype among 39 individuals in 2011, out of which 15 died. The sequences of the HA gene of these viruses belonged to the monophyletic clade, 2.2.1 which included viruses from humans and poultry [198]. In 2011, Egypt, with 158 confirmed H5N1 cases and 55 deaths, became the second country after Indonesia with the highest incidence of HPAIV disease. One more study reported an H5N1 outbreak in chickens. The infected chickens had symptoms of depression, facial edema, swollen cyanotic comb, and diffused hemorrhage. The outbreak reported 1170 chicken deaths per day. A total of 25 symptomatic chickens were sampled for virus isolation and characterization. The H5N1 subtype of influenza virus was confirmed using virus isolation and sequencing [199,200].

Another investigation identified that ferrets were naturally infected with H5N1 and it was suspected that the infection most probably spread via respiratory droplets to other exposed animals [201].

Human cases of rabies were reported in Egypt and it was observed that the virus was transmitted to humans through dog bites. Interestingly, the virus was successfully isolated from dogs, cats, farm animals, gerbils, and jackals [10,202]. Apart from rabies, at least five outbreaks of RVF were reported in Egypt in recent decades. These outbreaks have been reported to be linked to imported animals, mainly from other countries within the African continent. The virus has been isolated from humans and other animals, including camels, cows, sheep, buffaloes, goats, horses, and rats [202,203,204]. The largest reported RVF outbreak erupted in Egypt in 1977, infecting around 200,000 humans with 600 deaths [205].

The Middle East respiratory syndrome (MERS) is a zoonotic disease caused by lineage C of *Betacoronavirus*. MERS-CoV first emerged in Saudi Arabia in 2012, and was further transmitted to other neighboring countries in the region and other African countries. Direct contact with camels or the consumption of infected camel products are thought to be the factors responsible for the spread of the disease [206]. In different studies, dromedary camels have been found to be positive for MERS-CoV infection [207,208].

The CCHFV is the causative agent of hemorrhagic fever outbreaks reported in African, Middle Eastern, and Asian countries. CCHF is a tick-borne disease, which spreads through either biting or contact with infected animal tissues or blood. The disease was first reported in Egypt in 1976 in camels and sheep [209]. In a more recent sero-surveillance in 2009, one cow was found to be positive for the antibodies of CCHFV out of the 161 cattle that were sampled, while 2 cattle and 5 buffaloes were found to be positive for the RVFV antibodies [210]. In this investigation, blood samples were collected from imported as well as domestic animals that were getting slaughtered at an abattoir. In a rare unfortunate incident, an Egyptian virologist who mouth pipetted the culture of CCHFV isolate was reported dead [211], which suggested the potentially zoonotic nature of CCHFV.

The WNV is another deadly zoonotic virus, which is thought to be transmitted by migratory birds into new regions [8]. Mosquitoes of the genus *Culex* are reported as the main reservoir of the virus [36]. It has been reported that at a given time, there are approximately 350 resident and 150 migratory bird species in Egypt. The WNV was successfully isolated from white storks, which are migratory birds [37].

In a seroprevalence study conducted over a period of two years, 5965 people were recruited to participate in the study from different regions of the country. The detection of WNV using IgG antibodies revealed an approximately 24% (1431 human positive cases) seroprevalence among the subjects [212]. Interestingly, either *Culex* spp. or *Aedes* spp. were the most abundant mosquito species found in the study regions. Apart from humans, sentinel chickens also showed seroconversion, which was detected using ELISA [212].

Egypt has witnessed four major RVFV epidemics since 1977. The disease has affected cattle and sheep in the context of the abortion of pregnant animals. The disease has a high mortality rate of approximately 50% in young lambs, 25% to 35% in adult sheep, and around 25% in calves whereas an approximately 10% mortality in an adult cattle population has been recorded [213].

In 1978, 114 human cases were reported with 12 deaths. It was suggested that the import of infected camels was the source of the outbreak. Another hypothesis suggested that the import of sheep caused the introduction of the disease in the country [38]. Another outbreak appeared in 1993 among human and domestic animals [214]. In this outbreak, 128 confirmed human cases were reported, with 41 individuals having ocular disease [38]. It was also suggested that occupational exposure to infected tissues and blood, especially those working in slaughterhouses, put the population at risk of infection. The RVFV was isolated from the serum of sheep, cows, camels, goats, horses, and rats [203]. The highest number of virus isolates were obtained from sheep, indicating that sheep were more susceptible to infection compared to other animals. Interestingly, a very high number of human cases were also reported during this outbreak, and the virus was isolated from 53 out of 56 sera samples collected. Besides this, RVFV was also isolated from the throat washings of two and feces of four patients [215].

Respiratory disease symptoms, including facial edema, appeared in a six-week-old chicken flock in Alexandria Governorate during May 2011. Twenty swab samples, including tracheal and cloacal swabs, identified IAV H9N2 subtype in the infected chicken flock. This IAV H9N2 subtype appeared to be closely related to H9N2 subtype reported from Israel [216].

In March 2017, either sera samples or organ tissues from 16 ducks, including 9 live and 7 dead ducks, from a commercial flock as well as another 3 dead ducks from a backyard flock were either collected or submitted for investigation following a disease outbreak in ducks, reporting a high mortality of about 90%. In April 2017, three tracheal swab samples from dead backyard goslings were also submitted for investigation, raising the total samples to 22. The trachea, brain, and spleen samples from ducks and gooselings were found to be positive for H5 through RT-PCR analysis. The serology identified IAV H5N8 subtype in the nine sera samples collected from the live ducks. The duck infected with IAV H5N8 subtype exhibited lethargy with tremors and nervous symptoms like torticollis. Based on the phylogenetic analysis, this study suggested that migratory wild birds may have transmitted the highly pathogenic strain of H5N8 virus to the ducks, given their close relatedness with the European and Asian HA gene sequences deposited in GenBank [217].

Two naturally infected pigeons exhibiting respiratory disease and nervous symptoms were sampled for trachea and lung samples in two different geographical locations viz., Alexandria governorate and Behera, during 2009. To investigate the genomes of both birds, RNA was extracted and amplified for all eight gene segments of influenza virus using specific primers. Amplicons were run over agarose gel and then gel-purified DNA was ligated into the pGEM vector for cloning. Multiple clones were sequenced for each gene segment and aligned for analysis. The sequences were identified as the HPAIV H5N1 subtype. This was the first report of highly pathogenic H5N1 virus from naturally infected pigeons in Egypt [218].

In another investigation, five cloacal and five tracheal swabs were collected from asymptomatic bobwhite quails on a commercial quail farm in May 2011. Viral RNA was extracted and subjected to one-step real-time RT-PCR for the detection of the matrix gene of IAV as well as H5, H7, and H9 genes. The N2 gene was identified using a conventional RT-PCR. One H9N2-positive sample was passaged in embryonated chicken egg for virus isolation. Viral RNA was extracted and reverse transcribed followed by the amplification of 225 bp of the HA gene and 334 bp of the NA gene. Amplicons were sequenced and phylogenetic analysis revealed that the H9N2 virus retrieved from a bobwhite quail was similar to the H9N2 genome sequences reported from the Middle East and Israel [219]. This was the first report of IAV H9N2 subtype from a bobwhite quail in Egypt.

A seroprevalence of the IAV H7N7 subtype was conducted in poultry-exposed and non-exposed individuals over a period of three years. In the first year, 565 human sera samples from the poultry-exposed group and 150 sera samples from the unexposed group were collected. At the first follow up, which occurred a year after the first study, a total of 682 human sera samples from the exposed and 139 from the non-exposed individuals were taken. Subsequently, at the second follow up, 649 human sera from the poultry-exposed group and 104 sera from the non-exposed group were collected. A virus microneutralization test was conducted to assess the titer of antibodies against IAV H7N7 subtype. The positive sera samples were then tested with Western blot analysis followed by an immunofluorescence assay. The findings revealed that in the first year of the baseline study, all sera samples were negative for IAV H7N7 subtype. However, later, at the first follow-up investigation, 13 individuals (1.9%) from the poultry-exposed group were found to have antibodies against IAV H7N7 subtype. At the second follow-up investigation, 14 individuals (2.2%) from the poultry-exposed group were found to be positive for IAV H7N7 subtype. All controls (samples in the non-exposed group) were negative. The findings of this investigation suggested the transmission of IAV H7N7 subtype from poultry to poultry-exposed individuals [220].

Thirty-six nasal swabs and sera samples were collected from pig herds in Cairo in October 2013. The following years during December 2014–January 2015, 157 nasal swab samples were collected from the pigs at an abattoir located in Cairo. Additionally, 216 sera samples were also collected from pigs at the abattoir. RT-PCR revealed that all 36 swine nasal swab samples collected in October 2013 were negative for IAV. However, 122 out of 157 nasal swabs collected during 2014–2015 were found to be positive for IAV. The subtyping RT-PCR based on HA gene amplification was conducted to distinguish among the H5, H9, and A(H1N1)pdm09 viruses. It was found that 46 swine nasal swabs were found to be positive for the H5N1 subtype, seven were positive for the H9N2 subtype, and 69 samples were positive for the A(H1N1)pdm09 subtype. All samples were negative for human influenza virus H3N2 subtype. Interestingly, 5.6% of the samples were co-infected with avian influenza viruses H5N1 and H9N2 subtype while 1.9% samples were co-infected with avian influenza virus H5N1 and pandemic strain A(H1N1)pdm09 subtypes. More precisely, phylogeny based on two complete HA gene sequences of the A(H1N1)pdm09 virus revealed that these sequences appeared closely related to the 2009 pandemic virus. The partial HA gene sequence of the H5N1 subtype retrieved from swine in this study was found to be closely related to the avian influenza virus H5N1 subtype reported in 2015. The H9 sequences retrieved from swine were similar to the earlier reported Egyptian avian influenza virus H9N2 sequences [34].

In a targeted surveillance investigating the incidence of avian influenza virus in migratory birds in Lake Manzala, 19 cloacal and oropharyngeal samples were collected from either diseased or deceased birds being sold for food in a nearby live bird and fish market in Damietta Governorate on 24 November 2016. Interestingly, this was the same geographic location where the HPAIV H5N1 subtype was first identified and reported in the year 2006. In the current investigation, the sick migratory birds were reported as showing symptoms of mild depression. The sampled birds encompassed three species of migratory birds viz., common coot, pintail duck, and Garganey duck. Molecular investigation based on real-time RT-PCR identified two samples originating from common coot infected with IAV, which were subtyped by specific real-time RT-PCR and determined to be the HPAIV H5N8 subtype. The reported H5N8 subtype was successfully isolated and characterized by HA and NA gene sequencing followed by phylogenetic analysis. Immediately after this finding, the diagnostics were extended to the adjoining areas of Lake Manzala, where wild birds are usually noticed and poultry farming is rampant, but no other positive samples were retrieved in this extended investigation [221].

**Figure 14:**
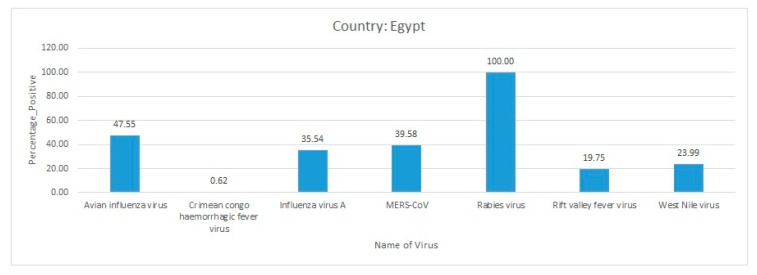
Distribution of virus diseases in Egypt in selected publications.

Rabies virus, avian influenza virus, MERS-CoV, IAV, RVFV, and WNV were the most widely distributed virus diseases reported from Egypt. A few cases of highly infectious CCHFV disease were also reported (Figure 14).

#### 3.4.3. Libya

After an outbreak of severe respiratory disease and high mortality among poultry in Libya in March 2013, tracheal and cloacal swab samples were collected from chickens for an investigation of the cause of the disease. Seventeen swabs were collected and processed for avian influenza virus and avian paramyxovirus-1 (APMV-1) detection using the real-time RT-PCR assay. Virus isolation was also attempted using specific pathogen free (SPF) embryonated chicken eggs. The samples that were positive for avian influenza viruses were further tested for subtyping using real-time RT-PCR for H5N1, H7, and H9 virus identification. After subtyping, positive samples were considered for sequencing. The molecular investigation reported nine APMV-1-positive samples while four chicken samples were positive for the LPAIV H9N2 subtype. Interestingly, one chicken sample was found to be infected with the HPAIV H5N1 subtype. This was the first study to report the HPAI H5N1 subtype and APMV-1 in Libya [222].

#### 3.4.4. Morocco

The A(H1N1)pdm09 virus was detected in the human population in Morocco during a study conducted between June and December 2009 at a Military Teaching Hospital in Rabat. Nasopharyngeal swabs from 594 outpatients exhibiting influenza-like disease symptoms, including cough, fever, muscle pain, vomiting, and diarrhea, were included in the study. Real-time RT-PCR was conducted to detect the A(H1N1)pdm09 virus, which found 240 positive samples [223].

A serological survey reported the incidence of IDV in cattle populations in Morocco [49]. In total, 200 samples were collected from cattle during 1991 to 2015. The HI and microneutralization assays determined that 35% of the cattle were positive for IDV [49]. The study also stated that 21,000 cattle had been reported to be imported from European countries to Morocco, but no export of cattle from Morocco to other countries was reported. This is interesting because the import of cattle from Europe was the most likely factor of IDV transmission given that IDV had already been reported from at least three continents [49].

Equine influenza virus H3N8 subtype was isolated using SPF chicken eggs from a mule showing respiratory disease symptoms in 1997. Further, the same virus was isolated from a diseased horse and a donkey in the year 2004. The NS gene of the three isolates was partially amplified and sequenced. The BLAST alignment followed by phylogenetic analysis of the partial NS gene sequences showed that the isolates recovered from 2004 and 1997 were closely related and were in circulation before 1990. Interestingly, the three isolates shared the same ancestry without any genetic reassortment [224].

Following a severe respiratory disease and high mortality among poultry in Morocco during January 2016, tissue samples were collected from the affected flocks showing severe respiratory disease and high mortality. A real-time RT-PCR-based investigation found that 10 samples were positive for IAV; however, all the samples were negative for NDV. The HA gene sequences of the retrieved IAV samples identified AIV H9N2 subtype in the affected poultry flocks in the country. This was the first report of H9N2 subtype from the country, which inflicted severe respiratory disease and high mortality among poultry flocks [225].

#### 3.4.5. Sudan

In 2005, an outbreak hit Sudan and human cases with hemorrhagic illness started appearing. Initially, these cases were suspected as a dengue fever illness and hence reported accordingly, until a yellow fever outbreak in the region was declared in November 2005. Following the declaration of the yellow fever outbreak, 605 symptomatic cases were considered for investigation from 42 different sites in the outbreak-hit areas in country. The symptoms included febrile illness, jaundice, hemorrhage, and eventually death in severe cases [226]. A total of 45% of the cases belonged to nomads or pastoral communities who were sampled and interviewed to find out the epidemiology of the disease. The investigation interestingly reported that a small rural area where the infected pastoral population stayed for some time while migrating to other locations notified the maximum number of symptomatic cases. The outbreak was so severe that 163 patients died out of 605 reported cases in a short period of time. A higher number of cases were found in males (61%) than females (39%). The collected blood samples were tested and several of them were found to be positive for YFV, chikungunya virus, dengue virus, and WNV [226]. Thirteen adult mosquitoes of the *Aedes aegypti* species were also collected from the outbreak regions, but this study could not correlate the outbreak with the mosquito vectors sampled in the region. According to the interviews taken from the reported cases, it was evident that the disease most likely spread from human to human and most of the cases were reported from rural areas [226].

Around the same time, another study reported a dengue hemorrhagic fever outbreak in children in Port Sudan. A total of 312 patients, where 229 were in the range of 5 to 15 years of age while the remaining were under five years of age, were suspected for dengue fever disease based on the symptoms [227]. In total, 12 patients (3.8%) died due to the severity of the disease. Due to the limitation of resources, only 40 patients were tested for dengue virus IgM, out of which 90% were positive. PCR analysis revealed that nine samples were positive for dengue virus serotype-3 (DEN-3); the alignment analysis of the sequences retrieved from PCR products confirmed a 99% identity with the DEN-3 isolates reported from Yemen during 2004–2005 and India in 2003 [227].

Another study reported the seroprevalence of dengue virus in 489 participants in Kassala in the year 2011. A total of 46/489 (9.4%) samples were positive for the dengue virus IgG and IgM using the ELISA assay [228]. In October 2012, the suspected cases of viral hemorrhagic fever were screened for YFV disease. Seven suspected cases were reported, which upon serological diagnostics with yellow fever IgM antibody followed by quantitative real-time PCR analysis, yielded four yellow fever-positive cases [229]. These reports revealed that dengue virus and YFV were in circulation and indeed, were among the fatal zoonotic diseases reported in the country.

Ebola virus disease was first reported in Sudan in the year 1974 from Nzara, where 284 cases were reported, out of which 151 people died of the disease. Later, in 1979, 34 more cases were reported from the same region, with 22 fatalities. Then, 17 cases were reported from Yambio in the year 2004, with seven fatal outcomes [71]. However, the animal source of infections for these cases was not determined, but since animal reservoirs of Ebola virus had been reported from central and west African countries, the possibility of transmission of virus from the wild to humans could not be ruled out [71].

#### 3.4.6. Tunisia

During the 2009 H1N1 pandemic, 7350 human samples, including nasopharyngeal and rhino-pharyngeal aspirations among other upper respiratory tract samples, were collected from several communities throughout Tunisia. The real-time RT-PCR-based diagnostics using the CDC protocol identified 3865 samples that were positive for influenza viruses. The following year, during 2010–2011, 181 samples were found to be positive for the influenza viruses out of 894 samples that were tested. Intriguingly, in this study, 19 human samples were found to be positive for IBV. The HA gene sequences of these 19 isolates revealed that two different IBV strains were in circulation. These IBV isolates were retrieved from patients exhibiting either mild (7 patients) or severe (12 patients) influenza-like illness. During 2009–2010, the B-Victoria lineage of IBV was in circulation while during 2010–2011, two lineages viz., B-Victoria and B-Yamagata, were in circulation. Phylogenetic analysis revealed that the IBV lineages that were in circulation were closely related to the IBV sequences reported from the neighboring countries of Algeria and Morocco [230].

A relatively lower seroprevalence of MERS-CoV was reported in dromedary camels during 2009–2013 compared to other African countries, including Ethiopia and Nigeria. in total, 155 dromedary camels were sampled in Sidi Bouzid province, targeting the 27 herds that were primarily raised for meat production. Additionally, sera samples from 39 camels were collected from 16 different herds in Kebili province from animals that were raised for tourist rides. Serological investigation based on IgG antibodies detected MERS-CoV in 30% of the dromedary camels. A total of 61 sera samples were found to be positive for MERS-CoV [47].

In summer 2014, blood samples were collected from 181 febrile patients attending Farhat Hached Hospital. Additionally, 38 blood samples were collected from apparently healthy abattoir workers with no febrile illness. The aim of the study was to investigate the cause of febrile illness. Patients and other subjects included in study were also asked for their travel history as well as any incident of tick bites. Simultaneously, ticks were also collected from the same region along with two national parks located in southern Tunisia during May–June 2014. Ticks were grouped into 46 pools. First of all, human sera samples were tested for WNV, but all of the samples were found to be negative. Then, samples were tested for CCHFV using IgM- and IgG-specific ELISA. Further, human and tick samples were also tested for CCHFV through real-time RT-PCR with oligonucleotide primers specific for the S segment of CCHFV. Only five patients from the hospital had IgM-specific antibodies in their sera representing a recent exposure to CCHFV, but all the sera samples were negative for IgG-specific antibodies. A total of 310 tick samples were subjected to screening for CCHFV; all were negative for CCHFV RNA using real-time RT-PCR. Only two (5.2%) of the abattoir workers were found to be seropositive for the CCHFV. No tick was found to be positive for CCHFV in this study. This investigation provided an evidence of CCHFV circulation in Tunisia [231].

**Figure 15:**
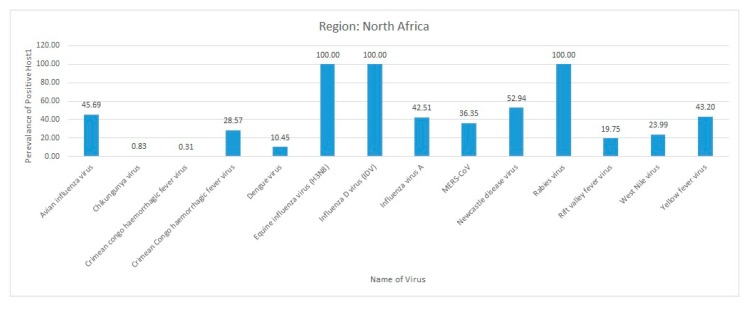
Distribution of virus diseases in North Africa in selected publications.

Briefly, YFV, MERS-CoV, RVFV, WNV, NDV, rabies virus, IAV, IDV, equine influenza virus H3N8 subtype, dengue virus, CCHFV, and chikungunya virus were reported to be in circulation in North African countries (Figure 15).

### 3.5. Southern Africa

Countries located in the southern part of Africa are Botswana, Lesotho, Namibia, South Africa, and the Kingdom of Eswatini (previously known as Swaziland).

#### 3.5.1. Botswana

A sero-surveillance was conducted to monitor the status of RVFV disease in African buffalo and cattle living at the interface of the Okavango Delta, Chobe National Park, and adjacent livestock areas located in Botswana during October 2010 and October 2011. In this investigation, sera samples from 863 cattle and 150 African buffaloes were collected and subjected to a virus neutralization test using anti-RVFV antibodies. Nineteen buffaloes and 49 cattle samples were found to be positive for RVFV antibodies. This was the first large-scale investigation of RVFV seroprevalence in the country. The findings of this study revealed that RVFV was in circulation at the cattle–wildlife interface in northern Botswana during 2010–2011 [232].

#### 3.5.2. Lesotho

During January 2012 through to March 2016, a total of 96 samples from one cat, 39 cattle, 41 dogs, 3 goats, 3 horses, 8 sheep, and one pig were collected and subjected to 2 different tests, with the aim of assessing the prevalence of rabies virus in the country along with the sensitivity of both tests. Samples were subjected to a direct rapid immunohistochemical test (DRIT) and direct florescent antibody (DFA) test. Additionally, 21 brain tissue samples were used for total RNA extraction followed by RT-PCR for the sequencing of the genome. The diagnostics confirmed that 72 of the 96 tested samples were positive for rabies virus infection. A sample obtained from a single cat was negative for the infection. In total, 30 of the 39 cattle samples were positive for the rabies virus using both diagnostic tests. Additionally, 36 of the 41 dogs were found to be positive for the rabies virus infection. Further, three goats, one horse, and six sheep were also found to be positive for the rabies virus. The phylogenetic analysis of the selected samples resulted in three clusters, where one cluster represented the rabies virus sequences retrieved from dogs while the sequences in the second cluster represented the relatedness with the rabies virus sequences reported from the KwaZulu-Natal province of South Africa. Interestingly, the sequences in the third cluster represented the samples originating within Lesotho and a few of the samples reported from the Free State Province of South Africa. The findings of this investigation reported the circulation of rabies virus among domestic animals in Lesotho [233]. 

#### 3.5.3. Namibia

During January 2009–July 2010, sera samples were collected from jackals in Etosha National Park to assess the seroprevalence of CDV and rabies virus. Sera samples were collected from 80 live- trapped jackals, 5 rabid jackals that were killed, and one jackal that was euthanized after an accident. A rabies fluorescent antibody virus neutralization test was conducted to assess the seroprevalence of rabies virus in the samples. Only seven sera samples were found to be positive for the rabies antibodies. On the other hand, a serum neutralization test was conducted to investigate the seroprevalence of CDV, which observed 62 positive samples. Four rabies virus isolates were successfully recovered from the brain tissues of the killed jackals. Sequencing and phylogenetic analysis revealed that these sequences were identical to the rabies virus isolates reported from the jackals, greater kudu, and dogs in the same geographic area. Therefore, these findings suggested a zoonotic transmission of rabies virus among dogs, greater kudu, and jackals in Etosha National Park [234].

Following a disease outbreak in African penguins (*Sphenisus demersus*) in Halifax island of Namibia, where in January- February 2019, more than 350 penguins were reported dead, and an investigation was launched. Penguins were reported to die after having severe disease symptoms, including torticollis, incoordination, corneal opacity, lethargy, and a state of comatose. The liver tissue sample from one symptomatic penguin and three cloacal samples from other sick penguins were collected and processed for RNA extraction. Quantitative RT-PCR detected the matrix gene sequence of the H5 strain of IAV. For further characterization of the virus, partial segments of the HA and NA gene sequences were amplified from the liver tissue and cloacal samples. Sequence analysis identified the HPAIV strain H5N8 in the penguin samples and revealed that the H5N8 virus in African penguins in Namibia is identical to the H5N8 virus reported in 2017 from South Africa [235], which suggested the circulation of the H5N8 virus in the southern African region.

After sheep and goats exhibited typical RVFV symptoms, especially in the border areas of South Africa, during May–July 2010, blood samples from live animals and tissue samples from various internal organs of dead animals were collected for an investigation of the disease. RNA was extracted from the blood samples and subjected to one-step RT-PCR for the amplification of the M segment of the RVFV genome for diagnostics. Virus isolation was carried out on RT-PCR-positive samples. Additionally, RT-PCR-positive amplicons were purified for direct sequencing. The investigation generated seven sequences originating from seven RVF outbreaks. The phylogenetic analysis revealed that the RVFV sequences from Namibia retrieved in 2010 were identical to the RVFV sequences reported from South Africa in the years 2009 and 2010, which suggested that the RVFV in both countries most probably originated from a single virus population [236].

Sixty-two free-ranging cheetahs were sampled during June 2002 through October 2004 in east-central Namibian farmlands. Additionally, three adult caracals (*Felis caracals*), four adult leopards, and one adult black-backed jackal (*Canis mesomelas*) were also sampled. Further, 24 captive cheetahs were also included in the study. Blood samples were collected from all the animals included in the study. Immunofluorescence assays were conducted using specific antibodies to investigate the prevalence of certain virus pathogens in the blood samples. As a result, three animals were found to be infected with feline herpesvirus (FHV), three were positive for feline calicivirus (FCV), five were positive for feline parvovirus (FPV), three were infected with feline coronavirus, eight were positive for CDV, and seven were found to be positive for rabies virus infections [237].

#### 3.5.4. South Africa

In 1961, following an epizootic event in Cape Province of South Africa that affected common terns (*Sterna hirundo*) with sickness and mortality, an avian influenza virus-like strain based on the morphology was isolated and characterized from either sick or dead birds. The etiological agent was termed as tern virus and was classified as influenza A virus/tern/South Africa/1961 [238]. Within a short period of time, 1300 dead terns were found within four small areas in the province. This was probably the first report of influenza virus disease from South Africa.

A recent study was conducted at an ostrich farm in the Western Cape province of South Africa, which was declared positive for the HPAIV strain H5N1. In this study, serum samples were collected from farm workers and veterinarians who had been in close contact with the ostriches on this farm. Intriguingly, two humans were found to be positive for H5N1 and four cases were positive for the LPAIV strain H7N1 [16]. This study provided evidence of the zoonotic transmission of avian influenza in South Africa.

In an earlier study reported in 1993, H7N1 virus was isolated from ostrich in the Western Cape province. The young ostriches at an ostrich farm had high mortality with a poor appetite, discoloration of urine, and ruffled feathers, among other symptoms [13]. The sub-type H7N1 of avian influenza was isolated from an infected and symptomatic ostrich. This study suggested the transmission of influenza virus most probably from wild birds visiting the farm [13]. In another study conducted in 2005, a large number of ostrich farms situated in the Western Cape province were found to be seropositive with the H5N2 strain of the influenza virus [239].

After five of the ostrich farms located within Oudtshoorn valley of the Western Cape province tested positive for the H5 virus, a thorough surveillance was initiated to screen other farms in the Klein Karoo area. Meanwhile, influenza-positive farms were quarantined from the exporting of ostrich meat. In a detailed investigation, until November 2011, a total of 42 ostrich farms were suspected as being affected by the disease. RNA-based diagnostics and sequencing confirmed that 14 of these suspected farms were positive for avian influenza outbreaks. This finding led to the slaughtering of 37,000 ostriches and the destruction of 3000 eggs. This outbreak caused huge economic losses for the ostrich industry in the country [240]. The molecular investigation and phylogenetic analysis revealed that the avian influenza H5N2 outbreak originated from a single source, a wild duck [5].

The IAV H5N8 subtype was reported from a broiler breeder farm located near Villiers in the Mpumalanga province on 19 June 2017. The intensity of the outbreak was the highest during August–September 2017. During the outbreak, a total of 40 isolates were successfully retrieved from commercial poultry and ostrich farms, as well as captive birds. This study suggested the role of wild birds in the introduction of the H5N8 virus strain from other African countries into South Africa and suspected that western African countries may be serving as the epicenter of the H5N8 virus. This was the first report of the HPAIV strain H5N8 from commercial poultry and captive birds in the country [241].

In a routine surveillance of the avian influenza virus prevalence in ostrich populations in the Western Cape province, approximately 13,000 sera, cloacal, and tracheal swab samples were collected during April 2011 to February 2012 in 8 rounds of sampling as well as samples deposited by state personnel. The ostriches exhibited symptoms like high fever, green coloration of urine, depression, and loss of appetite. There were significant mortalities among ostriches on the index farm identified in this investigation. RNA extraction for real-time RT-PCR followed by conventional RT-PCR and DNA sequencing identified the highly pathogenic H5N2 along with the H1N2 and H6N2 subtypes of avian influenza viruses across the 20 farms under investigation in this study [5].

After a pool of cloacal swab samples came up as RT-PCR positive for the IAV H5N2 subtype in April 2011, follow-up testing was done after a month in May 2011 as per the regulations. However, the 60 sera samples collected in April 2011 were negative for the H5 and H7 viruses, but since a pool of five cloacal swab samples were positive, the authorities had to follow the procedure to declare the index farm positive for H5N2 subtype, and as a result, the entire flock was supposed to undergo culling at an abattoir. Interestingly, since all 60 sera samples were found H5 and H7 negative, the farm owner expressed disagreement and felt that his birds were culled unfairly. As the set procedure, the entire ostrich flock was bled before culling at an abattoir and further tested for H5N2 subtype based on NP-specific ELISA. Out of 929 birds, 830 were found to be seropositive for H5N2 based on the AIV antibody ELISA test. To confirm the specificity of the above test, a subset of sera samples (366 samples) containing mostly positive samples identified by the AIV antibody ELISA test as well as some negative samples were further tested with two H5-specific ELISA and HI assays. In total, 292 of these 366 samples were positive for H5 subtype through all three ELISA assays, hence a consensus was established among the testing [242].

In another study, over a three-year surveillance during April 2012 to December 2014, a total of 17,762 sera, cloacal, and tracheal swab samples from wild birds, chickens, and ostriches across the country were submitted to testing for avian influenza virus diagnostics. The AIV antibody ELISA test identified several strains of avian influenza virus among the submitted samples. More precisely, ostrich samples were found to be positive for IAV H1N2, H5N2, H9N2, H6N1, H7N1, H7N7, H6N8, and H10N1 subtypes. Samples from Egyptian geese were found to be positive for the H5N2, H4N2, and H1N8 subtypes. The wild bird Cape Shoveller was found to be infected with H3N8 subtype while red-billed teals were found to be infected with the H4N8 and H11N2 subtypes. IAV H5N1 subtype was found to infect yellow-billed ducks while chicken samples were infected with H6N2 subtype. Pekin duck was infected with H10N7 subtype and shell duck was infected with the H7N8 subtype [5,243,244]. More recently, low-pathogenic avian influenza A virus was detected using molecular methods in wild birds for the first time in the KwaZulu-Natal province [245]. These findings suggested a widespread presence of avian influenza viruses in the country which requires programmed active surveillance in wild birds.

During an investigation of avian influenza viruses in 2014, a tracheal swab was found to be positive through the HI assay. The sample was further investigated to determine the virus through molecular techniques, hence RNA was extracted and subjected to total RNA sequencing. The genome sequence analysis found a complete genome of AOaV-1, which is known to cause Newcastle disease in avian species. This was the first complete genome of the AOaV-1 reported from the country. The genome sequences showed close identity with the AOaV-1 genome reported to GenBank from China, which was retrieved from a mallard. This finding indicated that wild waterfowls seem to harbor the AOaV-1 virus and appeared to be responsible for the long-distance dissemination of the virus to ostriches in a farm located within the Oudtshoorn district [246].

The first detection of influenza virus A(H1N1)pdm09 in South Africa was reported in a traveler who arrived in the country from the United States on 14 June 2009 having influenza-like illness for one day after the onset of the symptoms. Interestingly, the first locally acquired infection was reported from a person on 24 June 2009, following which seven other travel-associated A(H1N1)pdm09 virus infections were observed. By mid-July 2009, a total of 762 human samples had been tested, which resulted in 108 laboratory-confirmed cases of A(H1N1)pdm09 virus infections [247].

During February 2009–December 2012, a total of 16,005 patients were enrolled in a surveillance program known as severe acute respiratory illness (SARI) for the investigation of influenza-associated disease among hospitalized patients. Multiplex real-time RT-PCR detected 1239 influenza-positive samples. Influenza virus subtyping using the CDC-recommended molecular protocol identified 463 patients infected with the IAV subtype H3N2, while 338 patients were positive for the A(H1N1)pdm09 virus, and 418 patients had IBV infections. Twenty other influenza virus-positive samples could not be subtyped, most probably because of the low virus yield in those samples [248].

In a study conducted in Cape Peninsula, baboons were found to be positive for hepatitis A virus (HAV), cytomegalovirus, and Epstein–Barr virus using automated enzyme-linked fluorescent assays [249]. These baboons were reported to come into close contact with resident humans while raiding for dustbins in search of food, hence they pose a potential threat of transmitting virus zoonoses to humans.

In another study, 49 adult and 20 juvenile chacma baboons were caught from the wild and their sera samples were collected in the Limpopo, Western, and Eastern Cape provinces. DNA was extracted from the sera samples and subjected to nested PCR for the detection of HBV. The study resulted in 15 HBV-positive samples; 11 adults and four juvenile chacma baboons were infected. Southern hybridization resulted in 5 positive samples out of 15 given the low sensitivity compared to PCR. Additionally, liver tissue samples were also collected from one wild caught chacma baboon, which was ethically euthanized [250]. These liver tissue samples were used for the experimental infection of other baboons in this study.

As nonhuman primates are considered a reservoir of zoonotic virus infections, a recent report of a novel simian arterivirus in vervet monkeys is of high concern [81]. This virus is named Drakensberg Mountain vervet virus (DMVV-1), which poses a great threat to humans who may encounter these monkeys [81].

Flaviviruses are transmitted by mosquitoes and are of high importance in countries with tropical climates like South Africa. In a study conducted in Free State, cases of the transmission of flaviviruses among human, cattle, and sheep were identified. Kunjin virus antibodies detected Langat virus and suggested that other flaviviruses may also be in circulation in the area [251]. Apart from this, WNV and Wesselsbron virus were also identified among samples, which clearly suggested mosquito-borne transmission of these flaviviruses in Free State [251].

WNV is a flavivirus, and the first isolation of WNV was reported from the West Nile district of Uganda in 1937 [75]. WNV is transmitted by species of *Culex* mosquitoes while birds are reported to be the primary host [252]. Interestingly, vertebrate animals serve as the natural reservoir for WNV [253], with the exception of humans and horses, which are suggested to be the incidental hosts of WNV [254]. The first case of zoonotic transmission of WNV reported in the country was from a horse to an attending veterinarian while the second case was laboratory-originated transmission to the researcher due to a needle stick injury. Both cases developed neurological disease symptoms [254]. This study included specimens from horses for virus disease diagnostics: Six horses with neurological symptoms were diagnosed with WNV; interestingly, two horses were co-infected with African horse sickness virus (AHSV), which resulted in a fatal disease [255].

In another study conducted during 2008–2015 that documented 79 horses infected with WNV, interestingly, 14 WNV-infected horses were co-infected with other viruses: Three co-infections with AHSV, three with Sindbis virus, three with Shuni virus, and four with Middelburg virus [256]. There was one horse infected with equine encephalitis virus [256]. Typical WNV neurologic disease symptoms were observed in the horses. These findings suggested that horses may serve as the reservoir of WNV.

Horses are reported to be highly sensitive to some mosquito-borne flaviviruses, alphaviruses, and orthobunyaviruses, causing neurologic symptoms [255,257]. In a University of Pretoria-based investigation, seven horses were found to be infected with Shuni virus, five of which exhibited neurologic disease symptoms [258].

Sindbis fever is a mosquito-borne zoonotic infection caused by Sindbis virus, which was reported in South Africa for the first time in 1963 [259]. In a Sindbis virus disease surveillance in the country during 2006 to 2010, a total of 3631 samples from arbovirus-suspected patients were submitted for investigation, out of which 229 patients were found to be positive for Sindbis virus infection [260]. It was suggested that the higher disease prevalence among men than women is because of the greater participation in outdoor activities by men related to farm work compared to women, which make men more prone to mosquito bites [260].

During the Rift Valley fever outbreak in the country, two human cases of Wesselsbron virus disease were reported [261]. Wesselsbron virus is a mosquito-borne flavivirus, which primarily affects newly born goats and lambs with a high mortality rate. Wesselsbron virus disease may lead to abortions in pregnant ruminants [261]. The first cases of Wesselsbron disease were reported from the Free State. The Wesselsbron disease outbreak, in which five veterinary laboratory personnel were found symptomatic for Wesselsbron virus disease with four seroconversions and a single isolation, was reported in 1955 [262].

In another surveillance study among horses in the country, AHSV, Shuni virus, equine encephalitis virus, Sindbis virus, Middelburg virus, and WNV infections were reported during January 2008 to December 2013 [263]. Blood, tissue, and cerebrospinal fluid samples were tested to find the cause of the underlying febrile illness and neurologic symptoms among the horses. Under this surveillance, a total of 623 horses were investigated, out of which eight were diagnosed with Sindbis virus while 44 were infected with Middelburg virus [263]. There was one horse that was infected with only Sindbis virus, which had mild colic symptoms in addition to tongue paralysis and pale mucous membranes. Interestingly, the co-infection of WNV with Sindbis virus resulted in the death of two horses. The horses that were infected with Middelburg virus had febrile illness, and sometimes neurological symptoms, but death occurred in the severe cases of co-infections [263]. These viruses were reported to be vector-borne Old World alphaviruses, having zoonotic potential [264].

In an interesting case, a 13-year-old boy who spent a few days in a camp in Western Transvaal developed hemorrhagic fever with other symptoms after returning home. A tick of the *Hyalomma* species was found attached to his scalp. His condition deteriorated, with severe symptoms of hemorrhagic fever and gastro-intestinal bleeding, and he finally died on the sixth day after the onset of illness. The etiological agent was isolated and determined to be CCHFV in the laboratory in mice [39].

In a recent sero-epidemiologic survey that included abattoir workers, large animal veterinarians, farmers, horse handlers, hunters, and informal slaughterers, two cases were found to be positive for CCHFV IgG [265]. These two individuals were at an increased risk of tick exposure, suggesting that tick-borne CCHFV is of profound concern in the country in farming areas among animal handlers [265].

In another tragic incident, a travel agent from Zambia who lived on a small holding farm travelled to South Africa for a wedding. At the time of her travel, she was unaware of the infection and had preliminary mild symptoms of a cold and was on medication for suspected influenza. Her condition deteriorated after her return from the wedding and hence she was evacuated to a private hospital in Johannesburg. Due to complications, she died in the next few days while under treatment. Intriguingly, the attending paramedic and nurse developed similar symptoms and died due to severe complications, with symptoms of viral hemorrhagic fever [266]. The diagnosis confirmed an infection with Old World arenavirus. The reverse-transcription PCR procedure yielded approximately one 300-bp glycoprotein gene and another approximately 1000-bp nucleoprotein gene. The phylogenetic analysis confirmed that the etiological agent of the viral hemorrhagic fever was a novel arenavirus, hence it was named Lujo virus [266].

In another investigation, liver tissue samples from two deceased people were collected along with one sera sample. RNA was extracted and submitted for high-throughput pyrosequencing. The serum sample generated most of the arenavirus sequences compared to liver tissue biopsies. The gaps in the aligned sequences were filled with specific PCR reactions. The assembled genome was determined to be a classical bi-segmented arenavirus genome with two open reading frames. The phylogenetic analysis using the L or S segments of the genome identified that the retrieved genome belonged to Lujo virus. This was the first report illustrating genetic analysis and genome sequence-based characterization of Lujo virus from the country [267].

In a recent bio-surveillance study, Egyptian rousette bats (*Rousettus aegyptiacus*) based in Matlapitsi cave in the Gauteng province were diagnosed with Marburg virus infection. A total of 759 bats were tested as positive using ELISA. Genomic analysis performed on real time RT-PCR-positive samples confirmed the Ozolin strain of Marburg virus in bats [268]. The infestation of Egyptian rousette bats with Marburg virus puts the local population at an increased risk of spillover of zoonotic viruses in the event of an encounter.

In another investigation, coronaviruses were reported from South African bats. In this study, 113 archival gastrointestinal samples from 14 bat genera were subjected to investigation. For molecular diagnostics, 277 base-pair fragments of the RdRp gene were amplified and positive amplicons were sequenced. Only three bat specimens amplified the desired product. Phylogenetic analysis based on partial gene sequences identified that the South African bat coronaviruses clustered together with the *Alphacoronavirus* genus. This was the first report of coronaviruses from the South African bat population [269].

Five pet cats residing in Pinetown near Durban, East London, and Pietermaritzburg were investigated for lyssavirus infection, all of these pets showed aggressive behavior, disorientation, dehydration, and sometimes paralysis of the lower jaw. The cats were seen capturing rodents and occasionally were observed in contact with bats. They were suspected to be infected with rabies, hence they were tested against rabies antigen. The results were positive for rabies, but the fluorescence was more intense than what is usually seen in rabies cases; hence, another lyssavirus infection was suspected. A panel of antibodies was used that differentiated amongst lyssaviruses and confirmed that the cats were infected with Mokola virus [270]. The transmission of Mokola virus to cats was suspected to be either from bats as they have been reported to serve as the reservoir of several lyssaviruses or rodents and shrews, which also harbor and transmit lyssaviruses [271,272].

In another study, dog brain tissue samples were collected from the Mpumalanga and KwaZulu-Natal provinces and investigated for rabies infection using RNA extraction followed by reverse-transcription PCR and sequencing. The phylogenetic analysis confirmed that the virus sequences obtained from the Mpumalanga and KwaZulu-Natal provinces shared a common origin [273]. Interestingly, rabies from dogs has been transmitted to wild canids, including the species of jackal and foxes [274].

In another surveillance of rabies virus, 141 samples from dogs and three cattle samples collected from a nature reserve near Kruger National Park were found to be positive for rabies. On the other hand, two wildlife samples, one from spotted genet and another from baboon, were found to be positive for rabies virus infection [275]. The study suggested that the dogs acted as sentinels for rabies virus but not wildlife, hence it indicated the spread of the disease from dogs to wildlife [275].

A University of Pretoria-based study revealed that veterinarians working at the university had contracted virus zoonotic diseases, including rabies virus, RVFV, and WNV diseases, until the year 2001 [276].

The first cases of Rift Valley fever in the country were reported in Johannesburg in 1951 [277]. Another report of the disease appeared from the Western Cape and Free State provinces, where an outbreak occurred on sheep farms, causing heavy losses to farmers [278]. Interestingly, the veterinarians who performed autopsies or post-mortem on the diseased animals developed similar symptoms. Several farm workers who also reported developing symptoms of RVFV were individuals who cut open the infected sheep or animals [278]. These preliminary observations and studies revealed the zoonotic nature of the RVFV disease.

In a recent 2018 Rift Valley fever outbreak on a sheep farm in Free State, 250 fatalities or abortions in a flock of 600 sheep were reported. In this outbreak, out of 22 farm workers, 6 experienced symptoms comparable to the RVFV disease. Interestingly, four of these six symptomatic individuals came up positive for RVFV infection through the ELISA test [279]. This investigation suggested the zoonotic transmission of RVFV disease from sheep to humans through close contact.

In a country-wide RFV outbreak during 2008 to 2011, a total of 301 confirmed cases of RVFV infection were identified, out of which 25 died of the disease [280]. The common symptoms of RVFV reported from this outbreak included influenza-like illness with headache and fever, unexplained hemorrhagic illness or hepatitis, symptoms of encephalitis, etc. [280]. The high-risk category individuals were those in close contact with livestock and game animals, or those working in the slaughterhouses [280].

The Limpopo province represents a large swine industry, which has been hit in the past with African swine fever outbreaks. A study reported 1309 pigs that were infected with ASFV in the Limpopo province originated mainly because of the illegal trade and movement of pigs within the province [281].

In a recent study, cattle and goats were found to be seropositive for RVFV in Maputaland coastal plain, which is located far north in the KwaZulu-Natal province, with no earlier reported outbreak of the disease. In this study, 432 at least six-month-old cattle from small-scale farms, which were communally grazed, were bled from the coccygeal vein during November 2016 to June 2018 and tested for RVFV using IgG sandwich ELISA using IgG antibodies and the serum neutralization test (SNT). Seroprevalence of RVFV was found in 144 samples from cattle out of the 432 samples tested. These 144 seropositive samples were further tested with IgM ELISA and 5 samples were found to be positive. This study reported an overall 34% seroprevalence of RVFV among cattle in the designated study area in the northern KwaZulu-Natal province [282]. Additionally, 104 goats were bled from the jugular vein during February to April 2017 and tested for RVFV using the serum neutralization test. Interestingly, 33 goats (31.7%) were found to be seropositive for RVFV in this study. Intriguingly, a higher seroprevalence along the wetland in this study area was observed. This was the first report of RVFV among livestock in northern KwaZulu-Natal without an outbreak [282].

The first report of porcine circovirus type 2 (PCV2) infection in South African pigs was reported from a large-scale well-managed breeding farm in the Gauteng province along with postweaning multisystemic wasting syndrome during 2001 [283]. Pigs were seen to have clinical symptoms of porcine dermatitis, with a mortality rate below 10%. Brain, liver, spleen, and heart tissue samples from deceased pigs were taken for histopathology and immunohistochemistry. PCV2 infection in certain tissue types was seen through immunohistochemistry. Additionally, PCR amplification was used to detect PCV2 in different tissue samples obtained from dead pigs. Intriguingly, PCR successfully amplified PCV2 sequences in tissue samples obtained from four deceased pigs, confirming the infection. This was the first report of PCV2 infection in South African pigs [283].

In a recent study, 375 sera samples along with fecal and nasal swabs from healthy and diseased pigs were collected during 2015–2016 from three municipalities in the Eastern Cape province. A total of 339 samples were processed for genomic DNA extraction and the specific primer pairs were used for PCV2 detection among the samples. The initial screening was done through a PCR reaction followed by the subsequent testing of positive samples with other specific oligonucleotides. High-quality PCR products were purified on gel and sequenced for characterization of the virus. The investigation resulted in 54 PCV2-positive samples out of the 339 tested. This was the first report of PCV2 from the Eastern Cape province [284].

Later, 110 samples collected and archived during the 2015–2016 PCV2 study in the Eastern Cape province were randomly selected to assess the prevalence of porcine parvoviruses in the country. Conventional PCR amplification was used for the detection of porcine parvovirus DNA in the swine samples [285]. Positive samples were sequenced, and BLAST analysis followed by phylogeny was conducted for the identification and characterization of the porcine parvoviruses obtained in this study. Interestingly, 32 porcine parvovirus 1 (PPV1), 24 porcine parvovirus 2 (PPV2), 6 porcine parvovirus 3 (PPV3), 48 porcine parvovirus 4 (PPV4), 24 porcine bocavirus-like virus (PBo-likeV), and 49 porcine bocavirus 1 (PBoV1) and porcine bocavirus 2 (PBoV2) viruses were successfully detected in this investigation [285]. This report showed the prevalence of porcine parvoviruses in areas where communal swine farming is a common practice and demonstrated the presence of heterogeneous viruses in South African swine herds [285]. The PPV1 was successfully isolated for the first time in the country from a commercial piggery in 1975 [286].

In an analysis of the global surveillance of influenza virus infections among humans in 29 countries, 432 human cases of A(H1N1)pdm09 virus, 421 human infections of the H3N2 subtype, and 527 cases of IBV were reported during 1999–2014 in South Africa. Intriguingly, no human cases were reported for H1N1 virus infection during this period [52].

Human infection of HEV was detected in Cape Town; the infection was believed to have emerged from the consumption of food derived from pork meat. A total of 164 archived sera samples, which were earlier submitted to the diagnostic laboratory located at National Health Laboratory Service at Groote Schuur Hospital in Cape Town, which were negative for HAV, HBV, and HCV infections along with no other identifiable cause of disease, were investigated for HEV prevalence. The ELISA test using anti-HEV IgG antibodies detected 39 HEV-positive sera in this investigation while only two sera samples were positive for anti-HEV IgM antibodies. Interestingly, RT-PCR could not detect any HEV-positive samples. The findings of this investigation reported the seroprevalence and circulation of HEV in the Cape Town population. More interestingly, the investigation suggested that the probable cause of disease was the consumption of products prepared from pork meat [287].

The first complete genome sequence of a rotavirus was reported from a 10-week-old diarrheic piglet sampled at a Free State pig farm in the country in 2015. This was the first whole-genome sequence of a rotavirus reported from the African continent. For whole-genome sequencing, RNA was extracted from the fecal sample and was subjected to a sequence-independent amplification method. Sequencing was done by the ultra-deep sequencing method. The assembled sequences were analyzed using BLAST for the identification of the virus. Phylogenetic analysis revealed that these rotavirus sequences from South Africa clustered with the rotavirus sequences reported from Brazil, the USA, and Japan [288]. In another report, diarrhea was reported by a farmer in six-week-old piglets on a Warmbaths farm in northern Transvaal. Internal organ samples from two autopsied pigs were submitted for electron microscopic examination. Fecal samples from the pigs were negatively stained and examined under an electron microscope, which identified the particles of two distinct viruses. Based on the morphology (well-defined circular particles) and particle size, it was determined that the swine fecal material had rotavirus particles [289].

To investigate the prevalence of avian influenza virus and NDV in aquatic wild birds visiting ostrich farms located in the Oudtshoorn area in the Western Cape province, a study was conducted during May to July 1998. A total of 262 aquatic wild birds of different species were sampled from 19 different ostrich farms, which were sighted visiting the feeding bowls of ostriches on these farms. Additionally, 50 samples were included from non-aquatic birds, including pigeons, doves, and sparrows, in this study. Sera samples were screened for avian influenza virus using ELISA followed by the HI assay with specific antibodies to detect avian influenza viruses. Only eight aquatic wild bird samples were HI positive for avian influenza virus. These positive samples were characterized as H10N9 avian influenza virus. None of the non-aquatic birds were positive for the avian influenza virus in this investigation. This study reported the first H10N9 avian influenza virus isolate from the African continent [289]. Additionally, 46 aquatic wild birds were screened for NDV. Sera from these birds were tested through ELISA using NDV-specific antibodies. Surprisingly, a high seroprevalence of NDV was identified in this investigation, with 34 samples yielding positive results [290].

Orf virus is reported to trigger a zoonotic disease in ruminants and humans in the United Kingdom, the USA, and India, among other countries. An extensive investigation was launched during September 2009–March 2010 to find out the understanding and awareness of Orf virus disease in South Africa. Veterinarians and farmers were contacted, and relevant questionnaires were obtained to understand their response about the predominance of Orf virus disease within the country. Simultaneously, ruminants and cattle exhibiting Orf virus disease symptoms were included in the study. During the investigation, suspected cases of cows, goats, Boer goats, and sheep showing any lesions or scabs were sampled. DNA was extracted from the samples and a 594 base-pair sequence of the B2L gene, which represents a conserved region in the *Parapoxvirus* genus, was amplified. Amplicons were sequenced and sequence analysis of the nucleotides and amino acid alignments was carried out using the ClustalW method. This study successfully retrieved Orf virus sequences from six Boer goats, two goats, two sheep, and two cows. Interestingly, Orf virus infection was reported in one human individual who developed a blistering lesion on the thumb of one hand during the study period. This was the first study in the country that investigated the economic impact of the Orf virus disease and the transmission risk to humans [291].

Upper and lower respiratory tract specimens were collected from children up to five-year-old of age with respiratory disease who were admitted to hospitals during 1 January 2011 through 31 July 2015 across KwaZulu-Natal province, which is an endemic area for HIV prevalence. Multiplex PCR was conducted for the detection of respiratory syncytial virus (RSV), adenovirus, IAV, human rhinovirus, cytomegalovirus, and HIV. The results identified that 316 children were positive for RSV, 215 were positive for adenovirus, 152 were infected with human rhinovirus, and 50 children were positive for the IAV infections. Additionally, the study observed that 348 children were positive for HIV while 416 were found to be positive for cytomegalovirus [292].

Another study reported the implementation of severe acute respiratory illness surveillance among patients admitted to hospitals in KwaZulu-Natal, the Northwest province, Mpumalanga, and Gauteng during February 2009 to December 2013. Nasopharyngeal and oropharyngeal swabs were obtained from 7872 enrollees, who reported symptoms like fever, sore throat, cough, and/or difficulty in breathing. Multiplex real-time RT-PCR revealed that a large proportion of the admitted patients 5321/7039; 76%) were positive for HIV infection. RSV was detected in only 4% (329) of the patients. Interestingly, the frequency of RSV infection was similar in HIV-positive and HIV-negative patients [293].

In a small study, clippers from 50 barbers from three townships in Cape Town were collected. Clippers were immediately collected after a clean-shave haircut and other styled haircuts. Blood was detected on 42% of the clippers collected in this study. The total nucleic acid was extracted from each clipper and tested for HIV using nested qualitative RT-PCR. For the HIV screening, a 160- bp region of the gag gene was amplified. For the HBV testing, samples were subjected to the COBAS TaqMan HBV test. The positive samples for HBV DNA were further tested by qualitative nested PCR for confirmation. However, all the samples were negative for HIV, and four clippers were found to be positive for HBV infection. These four positive HBV clippers had enough DNA copies to trigger the transmission of the disease. This study revealed the risk of HBV transmission through clippers used in haircuts at barber shops. The sequences of HBV DNA retrieved from two of the clippers were closely related to the HBV sequences reported from the Gauteng province [294].

A general practitioner was called on 20 February 1970 to examine a 31-year-old white male South African farmer on a farm located approximately 100 km north-east of Pretoria. The patient was described to be in a high state of agitation with profuse sweating and muscle spasms of the back and neck. The patient was recently bitten on the lip by a bat, which was thought to be the cause of the disease. The initial disease symptoms were headache, muscle ache, sleeplessness, and difficulty in swallowing, which later progressed into profuse sweating and agitation. Muscular spasm and dyspnea of the mouth and throat muscles occurred at each attempt at drinking water. The patient was immediately admitted to a hospital in Pretoria with an initial diagnosis of rabies. At the hospital, the patient underwent intense pain and spasms of the pharyngeal muscles along with the arms and upper body parts whenever he was coaxed to drink water. The following night, the patient became violent and by the morning was seen frothing from the mouth, smashed the Vacoliter bottle to the wall, and kicked the attendants. Within 24 h after admission to the hospital, the patient died of respiratory arrest and seizure. The necropsy examination revealed that the brain was slightly congested while the lungs were severely congested along with other nervous system-related conditions. The brain tissues of the deceased were taken for a fluorescent antibody test of rabies but was found to be negative. Several attempts for rabies diagnostics were made but all were in vain. Experimental infection of mice introduced the ascending paralysis, culminating in death. It was considered a new virus strain and hence was given a new name ‘Duvenhage virus (DUVV)’. This was the first original report suggesting the zoonotic transmission of DUVV isolated from a South African male in 1970 [295].

**Figure 16:**
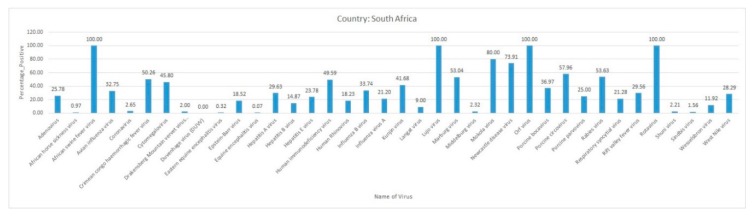
Distribution of virus diseases in South Africa in selected publications.

To summarize, a wide range of virus diseases have been reported in South Africa, including avian influenza virus, HIV, hepatitis A virus, hepatitis B virus, hepatitis E virus, IAV, IBV, Marburg virus, rabies virus, Mokola virus, WNV, RVFV, CCHFV, cytomegalovirus, African swine fever virus, rotavirus, respiratory syncytial virus, and Orf virus, among others (Figure 16). However, South Africa has large swine production systems, but there was no information available on the status of influenza virus disease in South African swine herds.

#### 3.5.5. Kingdom of Eswatini

The Kingdom of Eswatini (previously known as Swaziland) HIV Incidence Measurement Survey was conducted to assess the seroprevalence of HIV in the country between 10 December 2010 and 25 June 2011 across four administrative regions. This study identified 24,630 eligible individuals for participation, but only 18,177 individuals agreed to enroll for the study and HIV testing. HIV diagnostic results were available for 18,172 enrollees, out of which 5803 (32%) were determined to be HIV seropositive. This was a large fraction of the households that were positive for the disease. In a follow-up investigation, 11,897 HIV-seronegative individuals as identified in the last surveillance were investigated for the seroconversion rate among HIV-seronegative individuals. From this cohort, 11,232 individuals were accessible for the sampling and analysis. Therefore, a follow-up investigation between 23 August 2011 and 4 February 2012 included 11,232 individuals who were seronegative for HIV in the last testing and hence were sampled and re-tested for HIV seroprevalence. The outcome observed 145 HIV seroconversions among this cohort, resulting in a 2.4% incidence. The data analysis based on interviews identified that the highest seroprevalence of HIV was among men aged 30–34 years while higher seroconversion was among females who were either unmarried or those whose partners were living elsewhere. This was a nationwide cohort study to understand the current status of HIV disease in the country [296].

**Figure 17:**
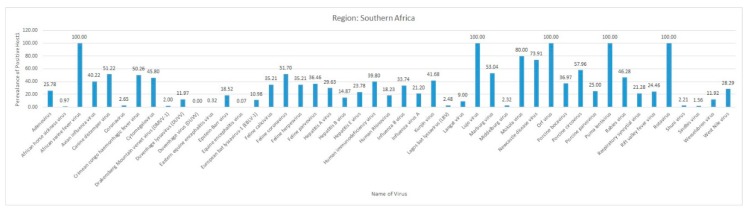
Distribution of virus diseases in Southern African countries in selected publications.

Overall, a wide range of virus diseases were reported from the countries located in the southern part of Africa (Figure 17).

### 3.6. Summary

The highest number of virus diseases were observed in South Africa, which might be explained by the improved disease surveillance in the country as compared to other African countries. However, despite large swine farming practices in South Africa, there is no report on the current status of influenza virus infections in pigs in South Africa. IAV and IBV infections have been reported from patients hospitalized in different healthcare facilities across the country, but there was no information on the animal reservoir host except reports of avian influenza viruses in wild birds and ostriches.

**Figure 18:**
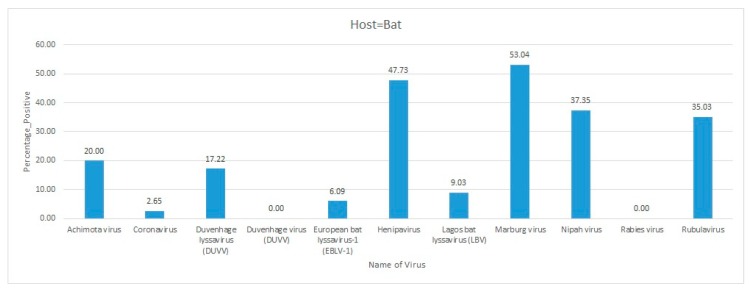
Distribution of virus diseases in African countries in bat species in selected publications.

Briefly, in this review, important virus diseases reported from countries in Africa were highlighted. The findings of this review are crucial in the context of the prevalence of virus diseases circulating across Africa and therefore, the data would be useful for designing studies focusing on virus diseases in Africa in the future. Interestingly, this review highlighted the circulation of Achimota virus, Duvenhage lyssavirus, Lagos bat lyssavirus, European bat lyssavirus, Achimota virus, Marburg virus, Nipah virus, and coronavirus through the bat species *E. helvum* (Figure 18). This information established bats as a potential natural reservoir for these zoonotic viruses that impact animal and human health.

**Figure 19:**
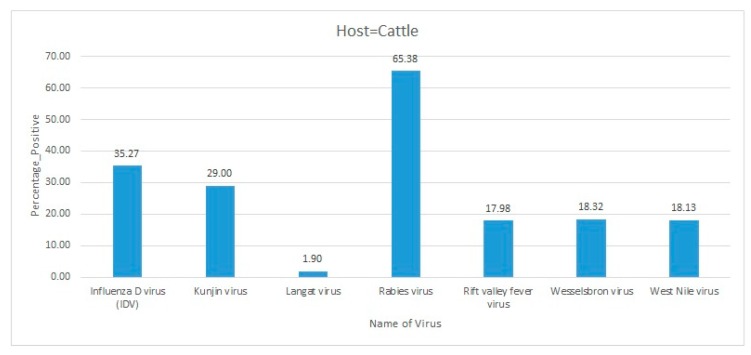
Distribution of virus diseases in African countries in cattle in selected publications.

Cattle were also found to be infected with rabies virus, IDV, Kunjin virus, RVFV, Langat virus, WNV, and Wesselsbron virus. The reports of IDV infections in cattle were relatively interesting in the context of the host range of IDV (Figure 19).

**Figure 20:**
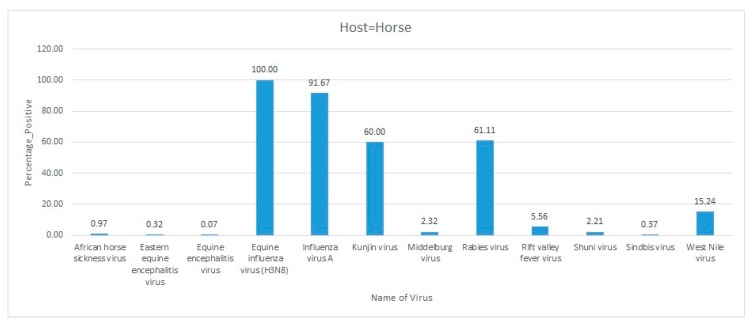
Distribution of virus diseases in African countries in equine in selected publications.

Additionally, horses were found to be infected mainly with equine influenza virus H3N8 subtype, IAV, rabies virus, RVFV, WNV, and Kunjin virus (Figure 20).

**Figure 21:**
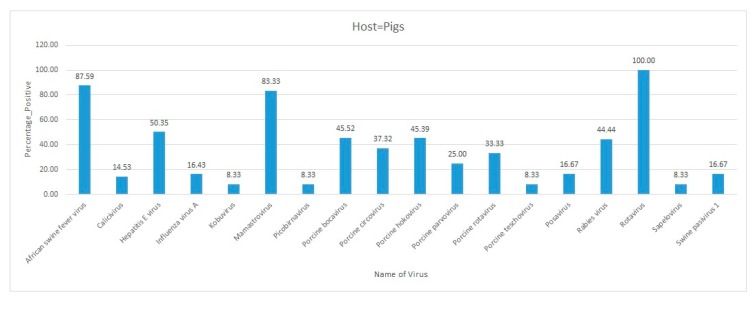
Distribution of virus diseases in African countries in pigs in selected publications.

**Figure 22:**
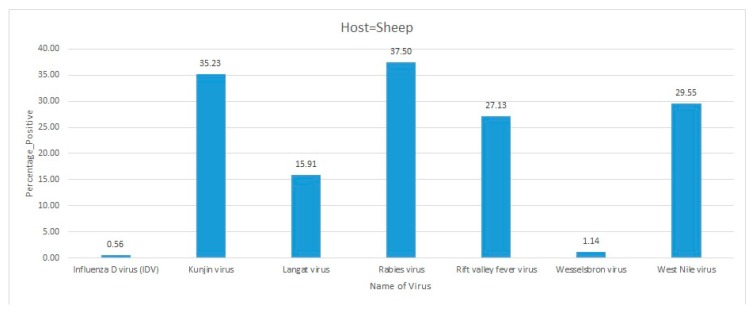
Distribution of virus diseases in African countries in sheep in selected publications.

This reflects that IAV, WNV, and RVFV have a wide host range. An IAV prevalence has been reported in pigs in Kenya [29]. Interestingly, pigs have recently been reported to be infected with the HPAI H5N1 subtype in Nigeria [30] and with A(H1N1)pdm09 virus in Nigeria, Ghana [31], Cameroon [32], and Togo [33]. Pigs were found to be positive for the HPAI H5N1 subtype and LPAI H9N2 subtype in Egypt during 2014–2015 [34]. Apart from this, hepatitis E virus, calicivirus, rabies virus, African swine fever virus, rotavirus, and several other viruses have been reported to infect pigs in African countries (Figure 21). Sheep were the other host that was reported to harbor RVFV, WNV, rabies virus, Langat virus, Kunjin virus, Wesselsbron virus, and IDV (Figure 22). Abortions and mortalities were reported among pregnant sheep that were infected with RVFV during gestation [213].

**Figure 23:**
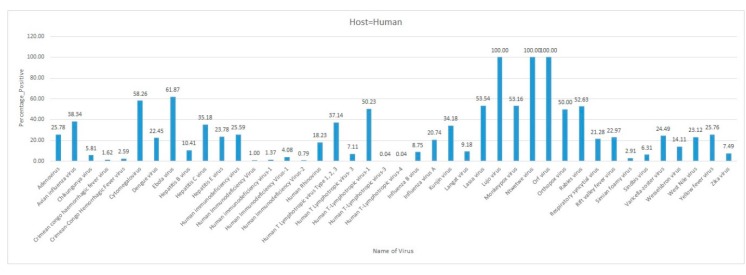
Distribution of reported virus diseases in humans in African countries in selected publications.

Some of the most serious virus infections reported in human populations across the continent included Ebola virus, HIV, IAV, Zika virus, WNV, RABV, CCHFV, and HCV, among many others (Figure 23).

**Figure 24:**
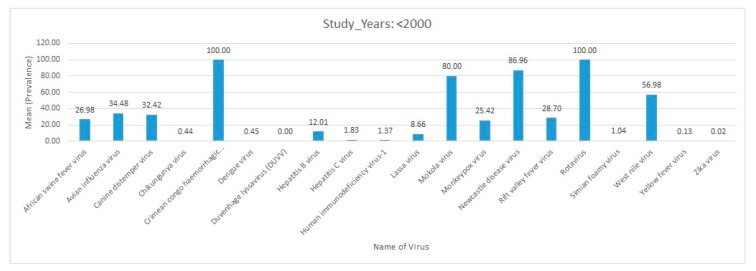
Distribution of the reported virus diseases in African countries before the year 2000 in selected publications.

**Figure 25:**
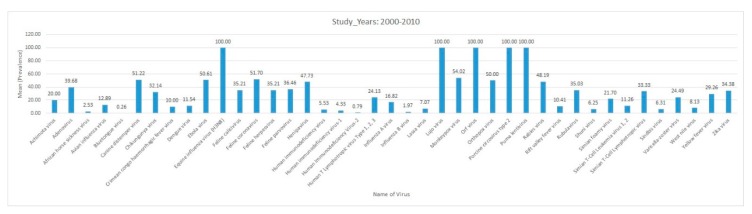
Distribution of the reported virus diseases in African countries between 2000 and 2010 in selected publications.

**Figure 26:**
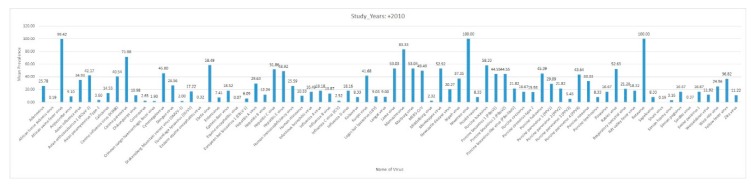
Distribution of the reported virus diseases in African countries after the year 2010 in selected publications.

This systematic review illustrates comprehensive information on important virus diseases reported from the African continent in light of the ‘One Health’ approach. Interestingly, the highest reported viruses in Africa before the year of 2000 were CCHFV, rotavirus, NDV, Mokola virus, and WNV (Figure 24). Between 2000 and 2010 (Figure 25), the highest reported viruses were equine influenza virus H3N8 subtype, Lujo virus, Orf virus, Puma virus, and porcine circovirus type 2, and after 2010 till the present date (Figure 26), the highest reported viruses were Ntwetwe virus, rotavirus, African swine fever virus, mamastrovirus, canine parvovirus, porcine bocavirus, and Ebola virus.

Virus disease pandemics have always been challenging for mankind, regardless of whether it was the 1918 Spanish influenza pandemic [297], the 2009 H1N1 pandemic, or the more recent SARS-CoV-2 pandemic in late December 2019; the latter is ongoing as of the date of this publication. One of the most crucial factors reported behind the transmission of influenza virus was its dependence on the relative humidity and temperature, which have proved to be important environmental conditions behind the emergence of influenza virus disease [298]. A high population density, rapidly changing environmental conditions, migration, and international travel coupled with rapid aging may facilitate virus dissemination and evolution, which may be crucial for the progression of emerging viral infectious diseases [299]. Recently, an Ebola virus disease outbreak was reported from the countries in West Africa. The zoonotic transmission of Ebola virus was reported from Cote d’ Ivoire in 1994 from chimpanzees to humans [71]. The Ebola virus disease was reported to be spread further through human-to-human contact and siblings within a household were found to be positive for the Ebola virus disease [109]. The disease affected people from different age groups and across occupations. The 2014 Ebola virus epidemic was reported to be the fourth largest epidemic in the African continent [109]. The management of such disease outbreaks requires huge efforts and funding [300,301]. Similarly, HIV-1 infection in Bantu and Pygmy hunter-gatherers in Cameroon was found due to their frequent contacts with non-human primates [1]. Bantu and Pygmies are forest-dwelling tribal communities in Cameroon, which usually remain in close contact with non-human primates and other wild animals. The prevalence of HIV-1 in Pygmies and Bantu individuals indicated the zoonotic potential of HIV-1 and its prevalence in tribal communities living in Cameroonian forests [1].

Likewise, the high seroprevalence of HIV-1 in people of Cameroon who have been reported to be in frequent contact with non-human primates either through hunting activities for bushmeat or butchering reflected the zoonotic and pandemic potential of HIV-1 [158]. WNV is another deadly zoonotic virus disease, which has been reported to be transmitted by migratory birds into new regions [8]. The significance of the environment in the emergence and re-emergence of infectious zoonotic virus diseases can be understood from the RVF outbreaks within Africa. The seroprevalence of anti-RVFV antibodies in ruminants, including cattle, goats, and sheep, living around wetland areas in Africa suggested the role of wet environmental conditions in disease progression [92]. Exceptional rainfalls led to flood-like situations in African grasslands, which have been reported to serve as favorable sites for the RVFV vector, the *Culex* mosquitoes; as a result, RVF outbreaks have occurred following exceptional rainfalls within Africa [302,303]. Rabies virus and MPXV, among several other potentially infectious virus diseases, have been reported from humans, animals, and birds within Africa.

It is noteworthy to mention that there are several yet undetected viruses circulating in wild and domestic animals. The close proximity of human populations to wild and domestic animals may facilitate virus adaptation and the emergence of virus strains with the potential to infect humans and may result in epidemics. It was previously reported that Canine morbillivirus, which are known to distribute among carnivores, requires continued treatment and monitoring, considering that CDV infections in non-human primates have already been demonstrated [304]. To date, there has been no evidence of a human infection by CDV; however, it was reported that CDV was isolated from a human cancer cell line [305]. In addition, further investigation is required to determine whether CDV can initiate a cross-species event in humans because of virus adaption [306].

With such a background, it is concluded that human health is interdependent on animal health as well as ecosystem health. To address such challenges, where a rapidly changing environment and animal health may impact human health, the framework of a ‘One Health’ approach comes into consideration [307]. The One Health approach is basically an integration of transdisciplinary sectors addressing animal, human, and ecosystem health for the well-being of the entire ecosystem [308,309]. The One Health approach may facilitate effective intervention in emerging infectious virus diseases through a multidisciplinary dimension from animal, human, and ecosystem perspectives, which simply would not be possible by adopting just a one-dimensional approach. The primary objective of the One Health approach is to mitigate the risk of disease outbreaks across all three constituent domains, including animals, humans, and the environment [307,309].

The recent emergence of zoonotic-origin SARS-CoV-2 [309,310] in December 2019 in Wuhan, China and its spread to more than 200 countries all over the world, as of the date of this publication, causing more than two million human infections worldwide and more than 130,000 deaths worldwide [311], is of great global public health concern. The WHO declared COVID-19 a pandemic on 11 March 2020 [312]. The COVID-19 pandemic is the first pandemic in history to be caused by a coronavirus (i.e., SARS-CoV-2), which reflects the importance of ‘One Health’ in detecting emerging zoonotic viruses of pandemic potential to improve public health [309]. In this context, programmed active surveillance for zoonotic viruses is deemed essential to fill the knowledge gaps, share information, bring awareness, and make informed decisions and strategies to be well-prepared early to combat or prevent the emergence of zoonotic viruses, which may lead to disease outbreaks in resource-limited countries in the African continent [313].

## 4. Conclusions

The current investigation reported several potential vector-borne and zoonotic disease-causing viruses that have been in circulation in Africa, including avian influenza virus, ASFV, swine influenza virus, HIV, simian immunodeficiency virus, WNV, RVFV, MPXV, Ebola virus, rabies virus, MERS-CoV, dengue virus, and Nipah virus, among several others. Multiple reports have observed an increasing trend of zoonotic virus diseases, especially Ebola virus and MPXV diseases, in western African countries; and influenza virus disease in countries, including Egypt, Kenya, Nigeria, and South Africa. In the past few years, Ebola has emerged as a challenging zoonotic disease with high mortality in Africa. Human-to-human transmission of Ebola virus through exposure to body fluids has been reported, which makes the human population vulnerable to the disease. The introduction of MPXV from wild reservoirs to human populations represents zoonotic transmission and it has been challenging to contain the disease to date. Interestingly, the hunting, butchering, and trading of bushmeat, especially in western and central Africa, including, Nigeria, Sierra Leone, and the Democratic Republic of Congo, among others, puts the human population at risk in terms of a zoonotic virus disease outbreak. Reports of an avian influenza virus from Egypt in domestic as well as commercial poultry and wild birds further increased the burden of disease in the continent. A report of swine influenza A virus from Kenya along with the finding of the HPAIV H5N1 subtype in a swine population in Nigeria and Egypt indicated the possibility of the transmission of influenza from wild birds to commercial pigs. Avian influenza was first reported in terns in South Africa in 1961. More recently, the highly pathogenic subtype of avian influenza was reported from an ostrich farm in the Western Cape province in South Africa. Interestingly, ASFV, which was reported to trigger hemorrhagic fever among the swine population, was reported in a commercial piggery in South Africa in 2013.

Overall, cultural practices, poor resources, illiteracy, lack of information and awareness, and limited or lack of active surveillance of vector-borne and zoonotic viral diseases would be considered the most probable factors behind the high burden of viral diseases in the African continent. Therefore, the findings of this study recommend that appropriate action plans must be urgently taken to increase awareness through extensive concerted and interdisciplinary efforts, and adequate education must be imparted in the context of possible virus disease outbreaks to stakeholders within such communities that may be prone to the acquisition of virus infections by different means. The launch of initiatives, such as ‘One Health’, for active virus disease surveillance at the interface of animals, humans, and the ecosystem is critical to devise and implement strategies to prevent or control the transmission and circulation of vector-borne and zoonotic virus diseases in Africa.

## Supplementary Materials

The following are available online at https://www.mdpi.com/2076-0817/9/4/301/s1. Table S1, zoonotic viruses reported from Africa in selected publications until September 2019.

## References

[1] Ndembi N., Habakkuk Y., Takehisa J., Takemura T., Kobayashi E., Ngansop C., Songok E., Miura T., Ido E., Hayami M. (2003). HIV type 1 infection in Pygmy hunter gatherers is from contact with Bantu rather than from nonhuman primates. AIDS Res. Hum. Retrovir..

[2] Lahm S.A., Kombila M., Swanepoel R., Barnes R.F. (2007). Morbidity and mortality of wild animals in relation to outbreaks of Ebola haemorrhagic fever in Gabon, 1994–2003. Trans. R. Soc. Trop Med. Hyg..

[3] World Health Organization (2020). Ebola Virus Disease- Democratic Republic of Congo, External Situation Report 82.

[4] Okareh O.T., Morakinyo O.M. (2018). Monkeypox in Nigeria: A case report of re-emerged disease outbreak. J. Microbiol. Exp..

[5] Abolnik C., Olivier A.J., Grewar J., Gers S., Romito M. (2012). Molecular analysis of the 2011 HPAI H5N2 outbreak in ostriches, South Africa. Avian Dis..

[6] Adeola O.A., Olugasa B.O., Emikpe B.O. (2017). Molecular detection of influenza A(H1N1)pdm09 viruses with M genes from human pandemic strains among Nigerian pigs, 2013–2015: Implications and associated risk factors. Epidemiol. Infect..

[7] Andriamandimby S.F., Marianneau P., Rafisandratantsoa J.T., Rollin P.E., Heraud J.M., Tordo N., Reynes J.M. (2011). Crimean-Congo hemorrhagic fever serosurvey in at-risk professionals, Madagascar, 2008 and 2009. J. Clin. Virol..

[8] Benjelloun A., El Harrak M., Belkadi B. (2016). West Nile Disease Epidemiology in North-West Africa: Bibliographical Review. Transbound Emerg. Dis..

[9] Budasha N.H., Gonzalez J.P., Sebhatu T.T., Arnold E. (2018). Rift Valley fever seroprevalence and abortion frequency among livestock of Kisoro district, South Western Uganda (2016): A prerequisite for zoonotic infection. BMC Vet. Res..

[10] David D., Hughes G.J., Yakobson B.A., Davidson I., Un H., Aylan O., Kuzmin I.V., Rupprecht C.E. (2007). Identification of novel canine rabies virus clades in the Middle East and North Africa. J. Gen. Virol..

[11] Krauss S., Webster R.G. (2010). Avian Influenza Virus Surveillance and Wild Birds: Past and Present. Avian Dis..

[12] Clark L., Hall J. (2006). Avian influenza in wild birds: Status as reservoirs, and risks to humans and agriculture. Ornithol. Monogr..

[13] Allwright D.M., Burger W.P., Geyer A., Terblanche A.W. (1993). Isolation of an influenza A virus from ostriches (Struthio camelus). Avian Pathol..

[14] Asante I.A., Bertram S., Awuni J., Commey A.N., Aniwa B., Ampofo W.K., Gabriel G. (2016). Highly Pathogenic Avian Influenza A(H5N1) Virus among Poultry, Ghana, 2015. Emerg. Infect. Dis..

[15] Ducatez M.F., Olinger C.M., Owoade A.A., Tarnagda Z., Tahita M.C., Sow A., De Landtsheer S., Ammerlaan W., Ouedraogo J.B., Osterhaus A.D. (2007). Molecular and antigenic evolution and geographical spread of H5N1 highly pathogenic avian influenza viruses in western Africa. J. Gen. Virol..

[16] Venter M., Treurnicht F.K., Buys A., Tempia S., Samudzi R., McAnerney J., Jacobs C.A., Thomas J., Blumberg L. (2017). Risk of Human Infections with Highly Pathogenic H5N2 and Low Pathogenic H7N1 Avian Influenza Strains During Outbreaks in Ostriches in South Africa. J. Infect. Dis..

[17] Si Y., Skidmore A.K., Wang T., de Boer W.F., Debba P., Toxopeus A.G., Li L., Prins H.H. (2009). Spatio-temporal dynamics of global H5N1 outbreaks match bird migration patterns. Geospat Health.

[18] Gschweng M., Kalko E.K., Querner U., Fiedler W., Berthold P. (2008). All across Africa: Highly individual migration routes of Eleonora’s falcon. Proc. Biol. Sci..

[19] Ofula V.O., Franklin A.B., Root J.J., Sullivan H.J., Gichuki P., Makio A., Bulimo W., Abong’o B.O., Muchai M., Schnabel D. (2013). Detection of Avian Influenza Viruses in Wild Waterbirds in the Rift Valley of Kenya Using Fecal Sampling. Vector-Borne Zoonot.

[20] Chen Y., Zhang J., Qiao C., Yang H., Zhang Y., Xin X., Chen H. (2013). Co-circulation of pandemic 2009 H1N1, classical swine H1N1 and avian-like swine H1N1 influenza viruses in pigs in China. Infect. Genet. Evol..

[21] Brown I.H. (2000). The epidemiology and evolution of influenza viruses in pigs. Vet. Microbiol..

[22] Hause B.M., Ducatez M., Collin E.A., Ran Z., Liu R., Sheng Z., Armien A., Kaplan B., Chakravarty S., Hoppe A.D. (2013). Isolation of a novel swine influenza virus from Oklahoma in 2011 which is distantly related to human influenza C viruses. PLoS Pathog.

[23] Meseko C.A., Odaibo G.N., Olaleye D.O. (2014). Detection and isolation of 2009 pandemic influenza A/H1N1 virus in commercial piggery, Lagos Nigeria. Vet. Microbiol..

[24] Nelson M.I., Gramer M.R., Vincent A.L., Holmes E.C. (2012). Global transmission of influenza viruses from humans to swine. J. Gen. Virol..

[25] Vincent A., Awada L., Brown I., Chen H., Claes F., Dauphin G., Donis R., Culhane M., Hamilton K., Lewis N. (2014). Review of influenza A virus in swine worldwide: A call for increased surveillance and research. Zoonoses Public Health.

[26] Webster R.G., Shortridge K.F., Kawaoka Y. (1997). Influenza: Interspecies transmission and emergence of new pandemics. FEMS Immunol. Med. Microbiol..

[27] Webster R.G. (1997). Influenza virus: Transmission between species and relevance to emergence of the next human pandemic. Arch. Virol. Suppl..

[28] Wentworth D.E., McGregor M.W., Macklin M.D., Neumann V., Hinshaw V.S. (1997). Transmission of swine influenza virus to humans after exposure to experimentally infected pigs. J. Infect. Dis..

[29] Munyua P., Onyango C., Mwasi L., Waiboci L.W., Arunga G., Fields B., Mott J.A., Cardona C.J., Kitala P., Nyaga P.N. (2018). Identification and characterization of influenza A viruses in selected domestic animals in Kenya, 2010–2012. PLoS ONE.

[30] Meseko C.A., Globig A., Ijomanta J., Joannis T., Nwosuh C., Shamaki D., Harder T., Hoffman D., Pohlmann A., Beer M. (2018). Evidence of exposure of domestic pigs to Highly Pathogenic Avian Influenza H5N1 in Nigeria. Sci. Rep..

[31] Adeola O.A., Olugasa B.O., Emikpe B.O. (2015). Detection of pandemic strain of influenza virus (A/H1N1/pdm09) in pigs, West Africa: Implications and considerations for prevention of future influenza pandemics at the source. Infect. Ecol. Epidemiol..

[32] Njabo K.Y., Fuller T.L., Chasar A., Pollinger J.P., Cattoli G., Terregino C., Monne I., Reynes J.M., Njouom R., Smith T.B. (2012). Pandemic A/H1N1/2009 influenza virus in swine, Cameroon, 2010. Vet. Microbiol..

[33] Ducatez M.F., Awoume F., Webby R.J. (2015). Influenza A(H1N1)pdm09 virus in pigs, Togo, 2013. Vet. Microbiol..

[34] Gomaa M.R., Kandeil A., El-Shesheny R., Shehata M.M., McKenzie P.P., Webby R.J., Ali M.A., Kayali G. (2018). Evidence of infection with avian, human, and swine influenza viruses in pigs in Cairo, Egypt. Arch. Virol..

[35] Oladipo E.K., Awoyelu E.H., Oloke J.K. (2018). Yellow fever, Dengue fever and West Nile viruses co-circulation in Ogbomoso. BioRxiv.

[36] McVey D.S., Wilson W.C., Gay C.G. (2015). West Nile virus. Rev. Sci Tech..

[37] Malkinson M., Banet C., Weisman Y., Pokamunski S., King R., Drouet M.T., Deubel V. (2002). Introduction of West Nile virus in the Middle East by migrating white storks. Emerg Infect. Dis.

[38] Kenawy M.A., Abdel-Hamid Y.M., Beier J.C. (2018). Rift Valley Fever in Egypt and other African countries: Historical review, recent outbreaks and possibility of disease occurrence in Egypt. Acta Trop.

[39] Gear J.H., Thomson P.D., Hopp M., Andronikou S., Cohn R.J., Ledger J., Berkowitz F.E. (1982). Congo-Crimean haemorrhagic fever in South Africa. Report of a fatal case in the Transvaal. S. Afr. Med. J..

[40] Liberati A., Altman D.G., Tetzlaff J., Mulrow C., Gotzsche P.C., Ioannidis J.P.A., Clarke M., Devereaux P.J., Kleijnen J., Moher D. (2009). The PRISMA Statement for Reporting Systematic Reviews and Meta-Analyses of Studies That Evaluate Health Care Interventions: Explanation and Elaboration. PLoS Med..

[41] Melade J., McCulloch S., Ramasindrazana B., Lagadec E., Turpin M., Pascalis H., Goodman S.M., Markotter W., Dellagi K. (2016). Serological Evidence of Lyssaviruses among Bats on Southwestern Indian Ocean Islands. PLoS ONE.

[42] Cosby M.T., Pimentel G., Nevin R.L., Fouad Ahmed S., Klena J.D., Amir E., Younan M., Browning R., Sebeny P.J. (2013). Outbreak of H3N2 influenza at a US military base in Djibouti during the H1N1 pandemic of 2009. PLoS ONE.

[43] Usman A., Ball J.D., Rojas D.P., Berhane A., Ghebrat Y., Mebrahtu G., Gebresellasie A., Zehaie A., Mufunda J., Liseth O. (2016). Dengue fever outbreaks in Eritrea, 2005–2015: A case for strengthening surveillance, control and reporting. Glob. Health Res. Policy.

[44] Hampson K., Coudeville L., Lembo T., Sambo M., Kieffer A., Attlan M., Barrat J., Blanton J.D., Briggs D.J., Cleaveland S. (2015). Correction: Estimating the global burden of endemic canine rabies. PLoS Negl. Trop Dis.

[45] Jemberu W.T., Molla W., Almaw G., Alemu S. (2013). Incidence of rabies in humans and domestic animals and people’s awareness in North Gondar Zone, Ethiopia. PLoS Negl. Trop Dis..

[46] Paulos A., Eshetu Y., Bethelhem N., Abebe B., Badeg Z. (2002). A study on the prevalence of animal rabies in Addis Ababa during 1999–2002. Ethiop. Vet. J..

[47] Reusken C.B., Messadi L., Feyisa A., Ularamu H., Godeke G.J., Danmarwa A., Dawo F., Jemli M., Melaku S., Shamaki D. (2014). Geographic distribution of MERS coronavirus among dromedary camels, Africa. Emerg Infect Dis..

[48] Sisay Z., Djikeng A., Berhe N., Belay G., Abegaz W.E., Wang Q.H., Saif L.J. (2016). First detection and molecular characterization of sapoviruses and noroviruses with zoonotic potential in swine in Ethiopia. Arch. Virol..

[49] Salem E., Cook E.A.J., Lbacha H.A., Oliva J., Awoume F., Aplogan G.L., Hymann E.C., Muloi D., Deem S.L., Alali S. (2017). Serologic Evidence for Influenza C and D Virus among Ruminants and Camelids, Africa, 1991–2015. Emerg Infect. Dis..

[50] Shieh W.J., Paddock C.D., Lederman E., Rao C.Y., Gould L.H., Mohamed M., Mosha F., Mghamba J., Bloland P., Njenga M.K. (2010). Pathologic studies on suspect animal and human cases of Rift Valley fever from an outbreak in Eastern Africa, 2006–2007. Am. J. Trop Med. Hyg..

[51] Amimo J.O., El Zowalaty M.E., Githae D., Wamalwa M., Djikeng A., Nasrallah G.K. (2016). Metagenomic analysis demonstrates the diversity of the fecal virome in asymptomatic pigs in East Africa. Arch. Virol..

[52] Caini S., Spreeuwenberg P., Kusznierz G.F., Rudi J.M., Owen R., Pennington K., Wangchuk S., Gyeltshen S., Ferreira de Almeida W.A., Pessanha Henriques C.M. (2018). Distribution of influenza virus types by age using case-based global surveillance data from twenty-nine countries, 1999–2014. BMC Infect. Dis..

[53] Apopo A.A., Kariithi H.M., Ateya L.O., Binepal Y.S., Sirya J.H., Dulu T.D., Welch C.N., Hernandez S.M., Afonso C.L. (2019). A retrospective study of Newcastle disease in Kenya. Trop. Anim. Health Prod..

[54] Ommeh S., Zhang W., Zohaib A., Chen J., Zhang H., Hu B., Ge X.Y., Yang X.L., Masika M., Obanda V. (2018). Genetic Evidence of Middle East Respiratory Syndrome Coronavirus (MERS-Cov) and Widespread Seroprevalence among Camels in Kenya. Virol. Sin..

[55] Raharinosy V., Olive M.M., Andriamiarimanana F.M., Andriamandimby S.F., Ravalohery J.P., Andriamamonjy S., Filippone C., Rakoto D.A.D., Telfer S., Heraud J.M. (2018). Geographical distribution and relative risk of Anjozorobe virus (Thailand orthohantavirus) infection in black rats (Rattus rattus) in Madagascar. Virol. J..

[56] Schwartz-Cornil I., Mertens P.P., Contreras V., Hemati B., Pascale F., Breard E., Mellor P.S., MacLachlan N.J., Zientara S. (2008). Bluetongue virus: Virology, pathogenesis and immunity. Vet. Res..

[57] Andriamandimby S.F., Viarouge C., Ravalohery J.P., Reynes J.M., Sailleau C., Tantely M.L., Elissa N., Cardinale E., Sall A.A., Zientara S. (2015). Detection in and circulation of Bluetongue virus among domestic ruminants in Madagascar. Vet. Microbiol..

[58] Chevalier V., Rakotondrafara T., Jourdan M., Heraud J.M., Andriamanivo H.R., Durand B., Ravaomanana J., Rollin P.E., Rakotondravao R. (2011). An unexpected recurrent transmission of Rift Valley fever virus in cattle in a temperate and mountainous area of Madagascar. PLoS Negl. Trop Dis..

[59] Temmam S., Besnard L., Andriamandimby S.F., Foray C., Rasamoelina-Andriamanivo H., Heraud J.M., Cardinale E., Dellagi K., Pavio N., Pascalis H. (2013). High prevalence of hepatitis E in humans and pigs and evidence of genotype-3 virus in swine, Madagascar. Am. J. Trop Med. Hyg..

[60] Peel A.J., Sargan D.R., Baker K.S., Hayman D.T.S., Barr J.A., Crameri G., Suu-Ire R., Broder C.C., Lembo T., Wang L.F. (2013). Continent-wide panmixia of an African fruit bat facilitates transmission of potentially zoonotic viruses. Nat. Commun..

[61] Haresnape J.M., Lungu S.A., Mamu F.D. (1985). A four-year survey of African swine fever in Malawi. J. Hyg. (Lond.).

[62] Haresnape J.M., Wilkinson P.J., Mellor P.S. (1988). Isolation of African swine fever virus from ticks of the Ornithodoros moubata complex (Ixodoidea: Argasidae) collected within the African swine fever enzootic area of Malawi. Epidemiol. Infect..

[63] Gudo E.S., Lesko B., Vene S., Lagerqvist N., Candido S.I., Razao de Deus N., Pinto F.D., Pinto G., Monteiro V., Evaristo V.L. (2016). Seroepidemiologic Screening for Zoonotic Viral Infections, Maputo, Mozambique. Emerg. Infect. Dis..

[64] Tivane A., Daniels R., Nguenha N., Machalele L., Nacoto A., Pale M., Mateonane E., Mavale S., Chilundo J., Muteto D. (2018). Antigenic and genetic characterization of influenza viruses isolated in Mozambique during the 2015 season. PLoS ONE.

[65] Sebastiani A., Aceti A., Paparo B.S., Pennica A., Ilardi I., Bile K., Mohamud O.M. (1985). Hepatitis B virus circulation in three different villages of Somalia. Trans. R. Soc. Trop Med. Hyg..

[66] Watts D.M., Corwin A.L., Omar M.A., Hyams K.C. (1994). Low risk of sexual transmission of hepatitis C virus in Somalia. Trans. R. Soc. Trop Med. Hyg..

[67] Baker K.S., Todd S., Marsh G.A., Crameri G., Barr J., Kamins A.O., Peel A.J., Yu M., Hayman D.T., Nadjm B. (2013). Novel, potentially zoonotic paramyxoviruses from the African straw-colored fruit bat Eidolon helvum. J. Virol..

[68] Cleaveland S., Appel M.G., Chalmers W.S., Chillingworth C., Kaare M., Dye C. (2000). Serological and demographic evidence for domestic dogs as a source of canine distemper virus infection for Serengeti wildlife. Vet. Microbiol..

[69] Roelke-Parker M.E., Munson L., Packer C., Kock R., Cleaveland S., Carpenter M., O’Brien S.J., Pospischil A., Hofmann-Lehmann R., Lutz H. (1996). A canine distemper virus epidemic in Serengeti lions (Panthera leo). Nature.

[70] Mtui-Malamsha N., Sallu R., Mahiti G.R., Mohamed H., OleNeselle M., Rubegwa B., Swai E.S., Makungu S., Otieno E.G., Lupindu A.M. (2019). Ecological and Epidemiological Findings Associated with Zoonotic Rabies Outbreaks and Control in Moshi, Tanzania, 2017–2018. Int. J. Environ. Res. Public Health.

[71] Leroy E.M., Epelboin A., Mondonge V., Pourrut X., Gonzalez J.P., Muyembe-Tamfum J.J., Formenty P. (2009). Human Ebola outbreak resulting from direct exposure to fruit bats in Luebo, Democratic Republic of Congo, 2007. Vector Borne Zoonotic Dis..

[72] Palmenberg A.C. (2017). Rhinovirus C, Asthma, and Cell Surface Expression of Virus Receptor CDHR3. J. Virol..

[73] Scully E.J., Basnet S., Wrangham R.W., Muller M.N., Otali E., Hyeroba D., Grindle K.A., Pappas T.E., Thompson M.E., Machanda Z. (2018). Lethal Respiratory Disease Associated with Human Rhinovirus C in Wild Chimpanzees, Uganda, 2013. Emerg. Infect. Dis..

[74] Edridge A.W.D., Deijs M., Namazzi R., Cristella C., Jebbink M.F., Maurer I., Kootstra N.A., Buluma L.R., van Woensel J.B.M., de Jong M.D. (2019). Novel Orthobunyavirus Identified in the Cerebrospinal Fluid of a Ugandan Child with Severe Encephalopathy. Clin. Infect. Dis..

[75] Smithburn K.C., Hughes T.P., Burke A.W., Paul J.H. (1940). A neurotropic virus isolated from the blood of a native of Uganda. Am. J. Trop. Med. Hyg..

[76] Go Y.Y., Balasuriya U.B., Lee C.K. (2014). Zoonotic encephalitides caused by arboviruses: Transmission and epidemiology of alphaviruses and flaviviruses. Clin Exp Vaccine Res..

[77] Byarugaba D.K., Erima B., Millard M., Kibuuka H., Lkwago L., Bwogi J., Mimbe D., Kiconco J.B., Tugume T., Mworozi E.A. (2016). Whole-genome analysis of influenza A(H1N1)pdm09 viruses isolated in Uganda from 2009 to 2011. Influenza Other Respir. Viruses.

[78] Ndumu D., Zecchin B., Fusaro A., Arinaitwe E., Erechu R., Kidega E., Kayiwa J., Muwanga E., Kirumira M., Kirembe G. (2018). Highly pathogenic avian influenza H5N8 Clade 2.3.4.4B virus in Uganda, 2017. Infect. Genet Evol..

[79] Simulundu E., Chambaro H.M., Sinkala Y., Kajihara M., Ogawa H., Mori A., Ndebe J., Dautu G., Mataa L., Lubaba C.H. (2018). Co-circulation of multiple genotypes of African swine fever viruses among domestic pigs in Zambia (2013–2015). Transbound Emerg Dis..

[80] Simulundu E., Sinkala Y., Chambaro H.M., Chinyemba A., Banda F., Mooya L.E., Ndebe J., Chitanga S., Makungu C., Munthali G. (2018). Genetic characterisation of African swine fever virus from 2017 outbreaks in Zambia: Identification of p72 genotype II variants in domestic pigs. Onderstepoort J. Vet. Res..

[81] Bailey A.L., Lauck M., Ghai R.R., Nelson C.W., Heimbruch K., Hughes A.L., Goldberg T.L., Kuhn J.H., Jasinska A.J., Freimer N.B. (2016). Arteriviruses, Pegiviruses, and Lentiviruses Are Common among Wild African Monkeys. J. Virol..

[82] Orba Y., Hang’ombe B.M., Mweene A.S., Wada Y., Anindita P.D., Phongphaew W., Qiu Y., Kajihara M., Mori-Kajihara A., Eto Y. (2018). First isolation of West Nile virus in Zambia from mosquitoes. Transbound Emerg Dis..

[83] Mweene-Ndumba I., Siziya S., Monze M., Mazaba M.L., Masaninga F., Songolo P., Mwaba P., Babaniyi O.A. (2015). Seroprevalence of West Nile Virus specific IgG and IgM antibodies in North-Western and Western provinces of Zambia. Afr. Health Sci..

[84] Kapiya J., Nalubamba K.S., Kaimoyo E., Changula K., Chidumayo N., Saasa N., Simuunza M.C., Takada A., Mweene A.S., Chitanga S. (2019). First genetic detection and characterization of canine parvovirus from diarrheic dogs in Zambia. Arch. Virol..

[85] Muleya W., Chambaro H.M., Sasaki M., Gwenhure L.F., Mwenechanya R., Kajihara M., Saasa N., Mupila Z., Mori-Kajihara A., Qiu Y. (2019). Genetic diversity of rabies virus in different host species and geographic regions of Zambia and Zimbabwe. Virus Genes.

[86] Simulundu E., Mweene A.S., Tomabechi D., Hang’ombe B.M., Ishii A., Suzuki Y., Nakamura I., Sawa H., Sugimoto C., Ito K. (2009). Characterization of H3N6 avian influenza virus isolated from a wild white pelican in Zambia. Arch. Virol..

[87] Simulundu E., Ishii A., Igarashi M., Mweene A.S., Suzuki Y., Hang’ombe B.M., Namangala B., Moonga L., Manzoor R., Ito K. (2011). Characterization of influenza A viruses isolated from wild waterfowl in Zambia. J. Gen. Virol..

[88] Wastika C.E., Sasaki M., Yoshii K., Anindita P.D., Hang’ombe B.M., Mweene A.S., Kobayashi S., Kariwa H., Carr M.J., Hall W.W. (2019). Serological evidence of Zika virus infection in non-human primates in Zambia. Arch. Virol..

[89] Caron A., Chiweshe N., Mundava J., Abolnik C., Capobianco Dondona A., Scacchia M., Gaidet N. (2017). Avian Viral Pathogens in Swallows, Zimbabwe: Infectious Diseases in Hirundinidae: A Risk to Swallow?. Ecohealth.

[90] Coetzer A., Gwenhure L., Makaya P., Markotter W., Nel L. (2019). Epidemiological aspects of the persistent transmission of rabies during an outbreak (2010–2017) in Harare, Zimbabwe. PLoS ONE.

[91] Couacy-Hymann E., Kouakou V.A., Aplogan G.L., Awoume F., Kouakou C.K., Kakpo L., Sharp B.R., McClenaghan L., McKenzie P., Webster R.G. (2012). Surveillance for influenza viruses in poultry and swine, west Africa, 2006–2008. Emerg. Infect. Dis..

[92] Boussini H., Lamien C.E., Nacoulma O.G., Kabore A., Poda G., Viljoen G. (2014). Prevalence of Rift Valley fever in domestic ruminants in the central and northern regions of Burkina Faso. Rev. Sci. Tech..

[93] Sanou A.M., Wandaogo S.C.M., Poda A., Tamini L., Kyere A.E., Sagna T., Ouedraogo M.S., Pauly M., Hubschen J.M., Muller C.P. (2018). Epidemiology and molecular characterization of influenza viruses in Burkina Faso, sub-Saharan Africa. Influenza Other Respir. Viruses.

[94] Zecchin B., Minoungou G., Fusaro A., Moctar S., Ouedraogo-Kabore A., Schivo A., Salviato A., Marciano S., Monne I. (2017). Influenza A(H9N2) Virus, Burkina Faso. Emerg. Infect. Dis..

[95] Dia N., Ndiaye M.N., Monteiro Mde L., Koivogui L., Bara M.O., Diop O.M. (2013). A subregional analysis of epidemiologic and genetic characteristics of influenza A(H1N1)pdm09 in Africa: Senegal, Cape Verde, Mauritania, and Guinea, 2009–2010. Am. J. Trop Med. Hyg..

[96] Lourie B., Bingham P.G., Evans H.H., Foster S.O., Nakano J.H., Herrmann K.L. (1972). Human infection with monkeypox virus: Laboratory investigation of six cases in West Africa. Bull. World Health Organ..

[97] Sklenovska N., Van Ranst M. (2018). Emergence of Monkeypox as the Most Important Orthopoxvirus Infection in Humans. Front. Public Health.

[98] Morozov V.A., Leendertz F.H., Junglen S., Boesch C., Pauli G., Ellerbrok H. (2009). Frequent foamy virus infection in free-living chimpanzees of the Tai National Park (Cote d’Ivoire). J. Gen. Virol..

[99] Ayouba A., Akoua-Koffi C., Calvignac-Spencer S., Esteban A., Locatelli S., Li H., Li Y., Hahn B.H., Delaporte E., Leendertz F.H. (2013). Evidence for continuing cross-species transmission of SIVsmm to humans: Characterization of a new HIV-2 lineage in rural Cote d’Ivoire. AIDS.

[100] Kouakou A.V., Kouakou V., Kouakou C., Godji P., Kouassi A.L., Krou H.A., Langeois Q., Webby R.J., Ducatez M.F., Couacy-Hymann E. (2015). Prevalence of Newcastle disease virus and infectious bronchitis virus in avian influenza negative birds from live bird markets and backyard and commercial farms in Ivory-Coast. Res. Vet. Sci..

[101] Bittaye M., Idoko P., Ekele B.A., Obed S.A., Nyan O. (2019). Hepatitis B virus sero-prevalence amongst pregnant women in the Gambia. BMC Infect Dis..

[102] Adeola O.A., Olugasa B.O., Emikpe B.O. (2016). Antigenic Detection of Human Strain of Influenza Virus A (H3N2) in Swine Populations at Three Locations in Nigeria and Ghana during the Dry Early Months of 2014. Zoonoses Public Health.

[103] Adeola O.A., Olugasa B.O., Emikpe B.O., Folitse R.D. (2019). Syndromic survey and molecular analysis of influenza viruses at the human-swine interface in two West African cosmopolitan cities suggest the possibility of bidirectional interspecies transmission. Zoonoses Public Health.

[104] Awuni J.A., Bianco A., Dogbey O.J., Fusaro A., Yingar D.T., Salviato A., Ababio P.T., Milani A., Bonfante F., Monne I. (2019). Avian influenza H9N2 subtype in Ghana: Virus characterization and evidence of co-infection. Avian Pathol..

[105] Dzotsi E.K., Ohene S.A., Asiedu-Bekoe F., Amankwa J., Sarkodie B., Adjabeng M., Thouphique A.M., Ofei A., Oduro J., Atitogo D. (2012). The first cases of Lassa fever in Ghana. Ghana Med. J..

[106] Punguyire D.T., Osei-Tutu A., Aleser E.V., Letsa T. (2017). Level and pattern of human rabies and dog bites in Techiman Municipality in the Middle Belt of Ghana: A six year retrospective records review. Pan. Afr. Med. J..

[107] Bonney J.H.K., Hayashi T., Dadzie S., Agbosu E., Pratt D., Nyarko S., Asiedu-Bekoe F., Ido E., Sarkodie B., Ohta N. (2018). Molecular detection of dengue virus in patients suspected of Ebola virus disease in Ghana. PLoS ONE.

[108] Ankrah G.A., Bonney J.H.K., Agbosu E.E., Pratt D., Adiku T.K. (2019). Serological evidence of Zika virus infection in febrile patients at Greater Accra Regional Hospital, Accra Ghana. BMC Res. Notes.

[109] Gatherer D. (2014). The 2014 Ebola virus disease outbreak in West Africa. J. Gen. Virol..

[110] International Ebola Response T., Agua-Agum J., Ariyarajah A., Aylward B., Bawo L., Bilivogui P., Blake I.M., Brennan R.J., Cawthorne A., Cleary E. (2016). Exposure Patterns Driving Ebola Transmission in West Africa: A Retrospective Observational Study. PLoS Med..

[111] Sissoko D., Keita M., Diallo B., Aliabadi N., Fitter D.L., Dahl B.A., Akoi Bore J., Raymond Koundouno F., Singethan K., Meisel S. (2017). Ebola Virus Persistence in Breast Milk After No Reported Illness: A Likely Source of Virus Transmission From Mother to Child. Clin. Infect. Dis..

[112] Mate S.E., Kugelman J.R., Nyenswah T.G., Ladner J.T., Wiley M.R., Cordier-Lassalle T., Christie A., Schroth G.P., Gross S.M., Davies-Wayne G.J. (2015). Molecular Evidence of Sexual Transmission of Ebola Virus. N. Engl. J. Med..

[113] Keita M., Duraffour S., Loman N.J., Rambaut A., Diallo B., Magassouba N., Carroll M.W., Quick J., Sall A.A., Glynn J.R. (2016). Unusual Ebola Virus Chain of Transmission, Conakry, Guinea, 2014–2015. Emerg. Infect. Dis..

[114] Diallo B., Sissoko D., Loman N.J., Bah H.A., Bah H., Worrell M.C., Conde L.S., Sacko R., Mesfin S., Loua A. (2016). Resurgence of Ebola Virus Disease in Guinea Linked to a Survivor with Virus Persistence in Seminal Fluid for More Than 500 Days. Clin. Infect. Dis..

[115] Bausch D.G., Demby A.H., Coulibaly M., Kanu J., Goba A., Bah A., Conde N., Wurtzel H.L., Cavallaro K.F., Lloyd E. (2001). Lassa fever in Guinea: I. Epidemiology of human disease and clinical observations. Vector Borne Zoonotic Dis..

[116] Demby A.H., Inapogui A., Kargbo K., Koninga J., Kourouma K., Kanu J., Coulibaly M., Wagoner K.D., Ksiazek T.G., Peters C.J. (2001). Lassa fever in Guinea: II. Distribution and prevalence of Lassa virus infection in small mammals. Vector Borne Zoonotic Dis..

[117] van Tienen C., de Silva T.I., Alcantara L.C., Onyango C.O., Jarju S., Goncalves N., Vincent T., Aaby P., Whittle H., Schim van der Loeff M. (2012). Molecular epidemiology of endemic human T-lymphotropic virus type 1 in a rural community in Guinea-Bissau. PLoS Negl. Trop Dis..

[118] Blackley D.J., Wiley M.R., Ladner J.T., Fallah M., Lo T., Gilbert M.L., Gregory C., D’Ambrozio J., Coulter S., Mate S. (2016). Reduced evolutionary rate in reemerged Ebola virus transmission chains. Sci. Adv..

[119] Durski K.N., McCollum A.M., Nakazawa Y., Petersen B.W., Reynolds M.G., Briand S., Djingarey M.H., Olson V., Damon I.K., Khalakdina A. (2018). Emergence of Monkeypox-West and Central Africa, 1970–2017. MMWR Morb. Mortal. Wkly Rep..

[120] Safronetz D., Sogoba N., Lopez J.E., Maiga O., Dahlstrom E., Zivcec M., Feldmann F., Haddock E., Fischer R.J., Anderson J.M. (2013). Geographic distribution and genetic characterization of Lassa virus in sub-Saharan Mali. PLoS Negl. Trop Dis..

[121] Keesing F., Belden L.K., Daszak P., Dobson A., Harvell C.D., Holt R.D., Hudson P., Jolles A., Jones K.E., Mitchell C.E. (2010). Impacts of biodiversity on the emergence and transmission of infectious diseases. Nature.

[122] Koita O.A., Sangare L., Poudiougou B., Aboubacar B., Samake Y., Coulibaly T., Pronyk P., Salez N., Kieffer A., Ninove L. (2012). A seroepidemiological study of pandemic A/H1N1(2009) influenza in a rural population of Mali. Clin. Microbiol. Infect..

[123] Bob N.S., Ba H., Fall G., Ishagh E., Diallo M.Y., Sow A., Sembene P.M., Faye O., El Kouri B., Sidi M.L. (2017). Detection of the Northeastern African Rift Valley Fever Virus Lineage During the 2015 Outbreak in Mauritania. Open Forum Infect Dis..

[124] Lagare A., Fall G., Ibrahim A., Ousmane S., Sadio B., Abdoulaye M., Alhassane A., Mahaman A.E., Issaka B., Sidikou F. (2019). First occurrence of Rift Valley fever outbreak in Niger, 2016. Vet. Med. Sci..

[125] Okoye J., Eze D., Krueger W.S., Heil G.L., Friary J.A., Gray G.C. (2013). Serologic evidence of avian influenza virus infections among Nigerian agricultural workers. J. Med. Virol..

[126] Munoz O., De Nardi M., van der Meulen K., van Reeth K., Koopmans M., Harris K., von Dobschuetz S., Freidl G., Meijer A., Breed A. (2016). Genetic Adaptation of Influenza A Viruses in Domestic Animals and Their Potential Role in Interspecies Transmission: A Literature Review. Ecohealth.

[127] Adeola O.A., Adeniji J.A. (2010). Prevalence of antibodies to influenza viruses among handlers of live pigs at three locations in Ibadan, Nigeria. Vet. Ital..

[128] Awosanya E.J., Ogundipe G., Babalobi O., Omilabu S. (2013). Prevalence and correlates of influenza-A in piggery workers and pigs in two communities in Lagos, Nigeria. Pan. Afr. Med. J..

[129] Snoeck C.J., Abiola O.J., Sausy A., Okwen M.P., Olubayo A.G., Owoade A.A., Muller C.P. (2015). Serological evidence of pandemic (H1N1) 2009 virus in pigs, West and Central Africa. Vet. Microbiol..

[130] Oluwayelu D.O., Bankole O., Ajagbe O., Adebiyi A.I., Abiola J.O., Otuh P., Omobowale O.T. (2014). Serological survey for emerging canine H3N8 and H3N2 influenza viruses in pet and village dogs in Nigeria. Afr. J. Med. Med. Sci..

[131] Faye O., Pratt C.B., Faye M., Fall G., Chitty J.A., Diagne M.M., Wiley M.R., Yinka-Ogunleye A.F., Aruna S., Etebu E.N. (2018). Genomic characterisation of human monkeypox virus in Nigeria. Lancet Infect Dis..

[132] Kabuga A.I., El Zowalaty M.E. (2019). A review of the monkeypox virus and a recent outbreak of skin rash disease in Nigeria. J. Med. Virol..

[133] World Health Organization (2018). Weekly Bulletin on Outbreaks and Other Emergencies. https://apps.who.int/iris/handle/10665/272981.

[134] Moore D.L., Causey O.R., Carey D.E., Reddy S., Cooke A.R., Akinkugbe F.M., David-West T.S., Kemp G.E. (1975). Arthropod-borne viral infections of man in Nigeria, 1964–1970. Ann. Trop. Med. Parasitol..

[135] Antia R.E., Adekola A.A., Jubril A.J., Ohore O.G., Emikpe B.O. (2018). Hepatitis E Virus infection seroprevalence and the associated risk factors in animals raised in Ibadan, Nigeria. J. Immunoass. Immunochem..

[136] Ogunro B.N., Olugasa B.O., Verschoor E.J., Olarinmoye A.O., Theyse I., Niphuis H. (2018). Serological Detection of Ebola Virus Exposures in Native Non-human Primates of Southern Nigeria. J. Epidemiol. Glob. Health.

[137] Welch C.N., Shittu I., Abolnik C., Solomon P., Dimitrov K.M., Taylor T.L., Williams-Coplin D., Goraichuk I.V., Meseko C.A., Ibu J.O. (2019). Genomic comparison of Newcastle disease viruses isolated in Nigeria between 2002 and 2015 reveals circulation of highly diverse genotypes and spillover into wild birds. Arch. Virol..

[138] Shittu I., Sharma P., Joannis T.M., Volkening J.D., Odaibo G.N., Olaleye D.O., Williams-Coplin D., Solomon P., Abolnik C., Miller P.J. (2016). Complete Genome Sequence of a Genotype XVII Newcastle Disease Virus, Isolated from an Apparently Healthy Domestic Duck in Nigeria. Genome Announc..

[139] Traore-Lamizana M., Zeller H.G., Mondo M., Hervy J.P., Adam F., Digoutte J.P. (1994). Isolations of West Nile and Bagaza viruses from mosquitoes (Diptera: Culicidae) in central Senegal (Ferlo). J. Med. Entomol..

[140] Chevalier V., Lancelot R., Diaite A., Mondet B., Sall B., De Lamballerie X. (2006). Serological assessment of West Nile fever virus activity in the pastoral system of Ferlo, Senegal. Ann. N. Y. Acad. Sci..

[141] Chevalier V., Reynaud P., Lefrancois T., Durand B., Baillon F., Balanca G., Gaidet N., Mondet B., Lancelot R. (2009). Predicting West Nile virus seroprevalence in wild birds in Senegal. Vector Borne Zoonotic Dis..

[142] Seck M.C., Badiane A.S., Thwing J., Moss D., Fall F.B., Gomis J.F., Deme A.B., Diongue K., Sy M., Mbaye A. (2019). Serological Data Shows Low Levels of Chikungunya Exposure in Senegalese Nomadic Pastoralists. Pathogens.

[143] Sow A., Faye O., Faye O., Diallo D., Sadio B.D., Weaver S.C., Diallo M., Sall A.A. (2014). Rift Valley fever in Kedougou, southeastern Senegal, 2012. Emerg. Infect. Dis..

[144] Arias A., Watson S.J., Asogun D., Tobin E.A., Lu J., Phan M.V.T., Jah U., Wadoum R.E.G., Meredith L., Thorne L. (2016). Rapid outbreak sequencing of Ebola virus in Sierra Leone identifies transmission chains linked to sporadic cases. Virus Evol..

[145] Alpren C., Sloan M., Boegler K.A., Martin D.W., Ervin E., Washburn F., Rickert R., Singh T., Redd J.T., Interagency Investigation T. (2016). Notes from The Field: Ebola Virus Disease Cluster-Northern Sierra Leone, January 2016. MMWR Morb. Mortal. Wkly Rep..

[146] Subissi L., Keita M., Mesfin S., Rezza G., Diallo B., Van Gucht S., Musa E.O., Yoti Z., Keita S., Djingarey M.H. (2018). Ebola Virus Transmission Caused by Persistently Infected Survivors of the 2014–2016 Outbreak in West Africa. J. Infect. Dis..

[147] Park D.J., Dudas G., Wohl S., Goba A., Whitmer S.L., Andersen K.G., Sealfon R.S., Ladner J.T., Kugelman J.R., Matranga C.B. (2015). Ebola Virus Epidemiology, Transmission, and Evolution during Seven Months in Sierra Leone. Cell.

[148] Hill S.C., Neto de Vasconcelos J., Granja B.G., Theze J., Jandondo D., Neto Z., Mirandela M., Sebastiao C.D.S., Candido A.L.M., Clemente C. (2019). Early Genomic Detection of Cosmopolitan Genotype of Dengue Virus Serotype 2, Angola, 2018. Emerg. Infect. Dis..

[149] Ntagirabiri R., Poveda J.D., Mumana A., Ndayishimiye H. (2014). Genotypes and subtypes of hepatitis C virus in Burundi: A particularity in sub-Saharan Africa. Pan. Afr. Med. J..

[150] Roy S., Vandenberghe L.H., Kryazhimskiy S., Grant R., Calcedo R., Yuan X., Keough M., Sandhu A., Wang Q., Medina-Jaszek C.A. (2009). Isolation and characterization of adenoviruses persistently shed from the gastrointestinal tract of non-human primates. PLoS Pathog..

[151] Larison B., Njabo K.Y., Chasar A., Fuller T., Harrigan R.J., Smith T.B. (2014). Spillover of pH1N1 to swine in Cameroon: An investigation of risk factors. BMC Vet. Res..

[152] Wade A., Jumbo S.D., Zecchin B., Fusaro A., Taiga T., Bianco A., Rodrigue P.N., Salomoni A., Kameni J.M.F., Zamperin G. (2018). Highly Pathogenic Avian Influenza A(H5N8) Virus, Cameroon, 2017. Emerg. Infect. Dis..

[153] Sadeuh-Mba S.A., Yonga Wansi G.M., Demanou M., Gessain A., Njouom R. (2018). Serological evidence of Rift VAlley fever Phlebovirus and Crimean-Congo hemorrhagic fever orthonairovirus infections among pygmies in the east region of Cameroon. Virol. J..

[154] Harvala H., Sharp C.P., Ngole E.M., Delaporte E., Peeters M., Simmonds P. (2011). Detection and genetic characterization of enteroviruses circulating among wild populations of chimpanzees in Cameroon: Relationship with human and simian enteroviruses. J. Virol..

[155] Pernet O., Schneider B.S., Beaty S.M., LeBreton M., Yun T.E., Park A., Zachariah T.T., Bowden T.A., Hitchens P., Ramirez C.M. (2014). Evidence for henipavirus spillover into human populations in Africa. Nat. Commun..

[156] Modiyinji A.F., Atsama M.A., Monamele G.C., Nola M., Njouom R. (2018). High seroprevalence of hepatitis E among pigs suggests an animal reservoir in Cameroon. J. Infect. Dev. Ctries..

[157] Switzer W.M., Garcia A.D., Yang C., Wright A., Kalish M.L., Folks T.M., Heneine W. (2008). Coinfection with HIV-1 and simian foamy virus in West Central Africans. J. Infect. Dis..

[158] LeBreton M., Yang O., Tamoufe U., Mpoudi-Ngole E., Torimiro J.N., Djoko C.F., Carr J.K., Tassy Prosser A., Rimoin A.W., Birx D.L. (2007). Exposure to wild primates among HIV-infected persons. Emerg. Infect. Dis..

[159] Zheng H., Wolfe N.D., Sintasath D.M., Tamoufe U., Lebreton M., Djoko C.F., Diffo Jle D., Pike B.L., Heneine W., Switzer W.M. (2010). Emergence of a novel and highly divergent HTLV-3 in a primate hunter in Cameroon. Virology.

[160] Wolfe N.D., Heneine W., Carr J.K., Garcia A.D., Shanmugam V., Tamoufe U., Torimiro J.N., Prosser A.T., Lebreton M., Mpoudi-Ngole E. (2005). Emergence of unique primate T-lymphotropic viruses among central African bushmeat hunters. Proc. Natl. Acad. Sci. USA.

[161] Calattini S., Betsem E., Bassot S., Chevalier S.A., Tortevoye P., Njouom R., Mahieux R., Froment A., Gessain A. (2011). Multiple retroviral infection by HTLV type 1, 2, 3 and simian foamy virus in a family of Pygmies from Cameroon. Virology.

[162] Calattini S., Betsem E.B., Froment A., Mauclere P., Tortevoye P., Schmitt C., Njouom R., Saib A., Gessain A. (2007). Simian foamy virus transmission from apes to humans, rural Cameroon. Emerg. Infect. Dis..

[163] Betsem E., Rua R., Tortevoye P., Froment A., Gessain A. (2011). Frequent and recent human acquisition of simian foamy viruses through apes’ bites in central Africa. PLoS Pathog..

[164] Wolfe N.D., Switzer W.M., Carr J.K., Bhullar V.B., Shanmugam V., Tamoufe U., Prosser A.T., Torimiro J.N., Wright A., Mpoudi-Ngole E. (2004). Naturally acquired simian retrovirus infections in central African hunters. Lancet.

[165] Courgnaud V., Van Dooren S., Liegeois F., Pourrut X., Abela B., Loul S., Mpoudi-Ngole E., Vandamme A., Delaporte E., Peeters M. (2004). Simian T-cell leukemia virus (STLV) infection in wild primate populations in Cameroon: Evidence for dual STLV type 1 and type 3 infection in agile mangabeys (*Cercocebus agilis*). J. Virol..

[166] Adlhoch C., Kaiser M., Kingsley M.T., Schwarz N.G., Ulrich M., de Paula V.S., Ehlers J., Lowa A., Daniel A.M., Poppert S. (2013). Porcine hokovirus in domestic pigs, Cameroon. Emerg. Infect. Dis..

[167] Monamele C.G., Karlsson E.A., Vernet M.A., Wade A., Okomo M.A., Abah A.S.A., Yann S., Etoundi G.A.M., Mohamadou N.R., Feussom J.M. (2019). Evidence of exposure and human seroconversion during an outbreak of avian influenza A(H5N1) among poultry in Cameroon. Emerg. Microbes. Infect..

[168] Besombes C., Gonofio E., Konamna X., Selekon B., Grant R., Gessain A., Berthet N., Manuguerra J.C., Fontanet A., Nakoune E. (2019). Intrafamily Transmission of Monkeypox Virus, Central African Republic, 2018. Emerg. Infect. Dis..

[169] Abakar M.F., Nare N.B., Schelling E., Hattendorf J., Alfaroukh I.O., Zinsstag J. (2014). Seroprevalence of Rift Valley fever, Q fever, and brucellosis in ruminants on the southeastern shore of Lake Chad. Vector Borne Zoonotic Dis..

[170] Twabela A.T., Tshilenge G.M., Sakoda Y., Okamatsu M., Bushu E., Kone P., Wiersma L., Zamperin G., Drago A., Zecchin B. (2018). Highly Pathogenic Avian Influenza A(H5N8) Virus, Democratic Republic of the Congo, 2017. Emerg. Infect. Dis..

[171] Hutin Y.J., Williams R.J., Malfait P., Pebody R., Loparev V.N., Ropp S.L., Rodriguez M., Knight J.C., Tshioko F.K., Khan A.S. (2001). Outbreak of human monkeypox, Democratic Republic of Congo, 1996 to 1997. Emerg. Infect. Dis..

[172] Learned L.A., Reynolds M.G., Wassa D.W., Li Y., Olson V.A., Karem K., Stempora L.L., Braden Z.H., Kline R., Likos A. (2005). Extended interhuman transmission of monkeypox in a hospital community in the Republic of the Congo, 2003. Am. J. Trop Med. Hyg..

[173] Meyer H., Perrichot M., Stemmler M., Emmerich P., Schmitz H., Varaine F., Shungu R., Tshioko F., Formenty P. (2002). Outbreaks of disease suspected of being due to human monkeypox virus infection in the Democratic Republic of Congo in 2001. J. Clin. Microbiol..

[174] Gessain A., Rua R., Betsem E., Turpin J., Mahieux R. (2013). HTLV-3/4 and simian foamy retroviruses in humans: Discovery, epidemiology, cross-species transmission and molecular virology. Virology.

[175] Falcone V., Leupold J., Clotten J., Urbanyi E., Herchenroder O., Spatz W., Volk B., Bohm N., Toniolo A., Neumann-Haefelin D. (1999). Sites of simian foamy virus persistence in naturally infected African green monkeys: Latent provirus is ubiquitous, whereas viral replication is restricted to the oral mucosa. Virology.

[176] Murray S.M., Linial M.L. (2006). Foamy virus infection in primates. J. Med. Primatol..

[177] Murray S.M., Picker L.J., Axthelm M.K., Hudkins K., Alpers C.E., Linial M.L. (2008). Replication in a superficial epithelial cell niche explains the lack of pathogenicity of primate foamy virus infections. J. Virol.

[178] Switzer W.M., Tang S., Ahuka-Mundeke S., Shankar A., Hanson D.L., Zheng H., Ayouba A., Wolfe N.D., LeBreton M., Djoko C.F. (2012). Novel simian foamy virus infections from multiple monkey species in women from the Democratic Republic of Congo. Retrovirology.

[179] Halawi A.D., Saasa N., Pongombo B.L., Kajihara M., Chambaro H.M., Hity M., Sawa H., Takada A., Mweene A.S., Nsembo L.L. (2019). Seroprevalence of Rift Valley fever in cattle of smallholder farmers in Kwilu Province in the Democratic Republic of Congo. Trop Anim. Health Prod..

[180] Xie D.D., Li J., Chen J.T., Eyi U.M., Matesa R.A., Obono M.M., Ehapo C.S., Yang L.Y., Yang H., Yang H.T. (2015). Seroprevalence of Human Immunodeficiency Virus, Hepatitis B Virus, Hepatitis C Virus, and Treponema pallidum Infections among Blood Donors on Bioko Island, Equatorial Guinea. PLoS ONE.

[181] Paupy C., Kassa Kassa F., Caron M., Nkoghe D., Leroy E.M. (2012). A chikungunya outbreak associated with the vector Aedes albopictus in remote villages of Gabon. Vector Borne Zoonotic Dis..

[182] Heymann D.L., Szczeniowski M., Esteves K. (1998). Re-emergence of monkeypox in Africa: A review of the past six years. Br. Med. Bull..

[183] Meyer A., Esposito J.J., Gras F., Kolakowski T., Fatras M., Muller G. (1991). First appearance of monkey pox in human beings in Gabon. Med. Trop (Mars).

[184] Mouinga-Ondeme A., Caron M., Nkoghe D., Telfer P., Marx P., Saib A., Leroy E., Gonzalez J.P., Gessain A., Kazanji M. (2012). Cross-species transmission of simian foamy virus to humans in rural Gabon, Central Africa. J. Virol..

[185] Mouinga-Ondeme A., Betsem E., Caron M., Makuwa M., Salle B., Renault N., Saib A., Telfer P., Marx P., Gessain A. (2010). Two distinct variants of simian foamy virus in naturally infected mandrills (Mandrillus sphinx) and cross-species transmission to humans. Retrovirology.

[186] Mouinga-Ondeme A., Kazanji M. (2013). Simian foamy virus in non-human primates and cross-species transmission to humans in Gabon: An emerging zoonotic disease in central Africa?. Viruses.

[187] Liegeois F., Boue V., Mouacha F., Butel C., Ondo B.M., Pourrut X., Leroy E., Peeters M., Rouet F. (2012). New STLV-3 strains and a divergent SIVmus strain identified in non-human primate bushmeat in Gabon. Retrovirology.

[188] Nurtop E., Moyen N., Dzia-Lepfoundzou A., Dimi Y., Ninove L., Drexler J.F., Gallian P., de Lamballerie X., Priet S. (2019). A Report of Zika Virus Seroprevalence in Republic of the Congo. Vector Borne Zoonotic Dis..

[189] Umuhoza T., Berkvens D., Gafarasi I., Rukelibuga J., Mushonga B., Biryomumaisho S. (2017). Seroprevalence of Rift Valley fever in cattle along the Akagera-Nyabarongo rivers, Rwanda. J. S. Afr. Vet. Assoc..

[190] Wane J., Nyatanyi T., Nkunda R., Rukelibuga J., Ahmed Z., Biedron C., Kabeja A., Muhimpundu M.A., Kabanda A., Antara S. (2012). 2009 pandemic influenza A (H1N1) virus outbreak and response–Rwanda, October, 2009–May, 2010. PLoS ONE.

[191] Yen T.Y., Trovoada dos Santos Mde J., Tseng L.F., Chang S.F., Cheng C.F., Carvalho A.V., Shu P.Y., Lien J.C., Tsai K.H. (2016). Seroprevalence of antibodies against dengue virus among pregnant women in the Democratic Republic of Sao Tome and Principe. Acta Trop.

[192] Formenty P., Muntasir M.O., Damon I., Chowdhary V., Opoka M.L., Monimart C., Mutasim E.M., Manuguerra J.C., Davidson W.B., Karem K.L. (2010). Human monkeypox outbreak caused by novel virus belonging to Congo Basin clade, Sudan, 2005. Emerg. Infect. Dis..

[193] Jeevan T., Darnell D., Gradi E.A., Benali Y., Kara R., Guetarni D., Rubrum A., Seiler P.J., Crumpton J.C., Webby R.J. (2019). A(H9N2) influenza viruses associated with chicken mortality in outbreaks in Algeria 2017. Influenza Other Respir Viruses.

[194] Kautman M., Tiar G., Papa A., Siroky P. (2016). AP92-like Crimean-Congo Hemorrhagic Fever Virus in Hyalomma aegyptium Ticks, Algeria. Emerg. Infect. Dis..

[195] Laabassi F., Lecouturier F., Amelot G., Gaudaire D., Mamache B., Laugier C., Legrand L., Zientara S., Hans A. (2015). Epidemiology and Genetic Characterization of H3N8 Equine Influenza Virus Responsible for Clinical Disease in Algeria in 2011. Transbound Emerg. Dis..

[196] Kandeel A., Manoncourt S., Abd el Kareem E., Mohamed Ahmed A.N., El-Refaie S., Essmat H., Tjaden J., de Mattos C.C., Earhart K.C., Marfin A.A. (2010). Zoonotic transmission of avian influenza virus (H5N1), Egypt, 2006–2009. Emerg. Infect. Dis..

[197] Saad M.D., Ahmed L.S., Gamal-Eldein M.A., Fouda M.K., Khalil F., Yingst S.L., Parker M.A., Montevillel M.R. (2007). Possible avian influenza (H5N1) from migratory bird, Egypt. Emerg. Infect. Dis..

[198] Younan M., Poh M.K., Elassal E., Davis T., Rivailler P., Balish A.L., Simpson N., Jones J., Deyde V., Loughlin R. (2013). Microevolution of highly pathogenic avian influenza A(H5N1) viruses isolated from humans, Egypt, 2007–2011. Emerg. Infect. Dis..

[199] Bahgat M.M., Kutkat M.A., Nasraa M.H., Mostafa A., Webby R., Bahgat I.M., Ali M.A. (2009). Characterization of an avian influenza virus H5N1 Egyptian isolate. J. Virol Methods.

[200] Mostafa A., Abdelwhab E.M., Mettenleiter T.C., Pleschka S. (2018). Zoonotic Potential of Influenza A Viruses: A Comprehensive Overview. Viruses.

[201] Arafa A.S., Yamada S., Imai M., Watanabe T., Yamayoshi S., Iwatsuki-Horimoto K., Kiso M., Sakai-Tagawa Y., Ito M., Imamura T. (2016). Risk assessment of recent Egyptian H5N1 influenza viruses. Sci. Rep..

[202] Helmy Y.A., El-Adawy H., Abdelwhab E.M. (2017). A Comprehensive Review of Common Bacterial, Parasitic and Viral Zoonoses at the Human-Animal Interface in Egypt. Pathogens.

[203] Imam I.Z., El-Karamany R., Darwish M.A. (1979). An epidemic of Rift Valley fever in Egypt. 2. Isolation of the virus from animals. Bull. World Health Organ..

[204] Samy A.M., Peterson A.T., Hall M. (2017). Phylogeography of Rift Valley Fever Virus in Africa and the Arabian Peninsula. PLoS Negl. Trop Dis..

[205] Meegan J.M., Hoogstraal H., Moussa M.I. (1979). An epizootic of Rift Valley fever in Egypt in 1977. Vet. Rec..

[206] Han H.J., Yu H., Yu X.J. (2016). Evidence for zoonotic origins of Middle East respiratory syndrome coronavirus. J. Gen. Virol..

[207] Ali M.A., Shehata M.M., Gomaa M.R., Kandeil A., El-Shesheny R., Kayed A.S., El-Taweel A.N., Atea M., Hassan N., Bagato O. (2017). Systematic, active surveillance for Middle East respiratory syndrome coronavirus in camels in Egypt. Emerg. Microbes Infect..

[208] Perera R.A., Wang P., Gomaa M.R., El-Shesheny R., Kandeil A., Bagato O., Siu L.Y., Shehata M.M., Kayed A.S., Moatasim Y. (2013). Seroepidemiology for MERS coronavirus using microneutralisation and pseudoparticle virus neutralisation assays reveal a high prevalence of antibody in dromedary camels in Egypt, June 2013. Euro Surveill.

[209] Darwish M.A., Imam I.Z., Omar F.M., Hoogstraal H. (1978). Results of a preliminary seroepidemiological survey for Crimean-Congo hemorrhagic fever virus in Egypt. Acta Virol..

[210] Horton K.C., Wasfy M., Samaha H., Abdel-Rahman B., Safwat S., Abdel Fadeel M., Mohareb E., Dueger E. (2014). Serosurvey for zoonotic viral and bacterial pathogens among slaughtered livestock in Egypt. Vector Borne Zoonotic Dis..

[211] Weidmann M., Avsic-Zupanc T., Bino S., Bouloy M., Burt F., Chinikar S., Christova I., Dedushaj I., El-Sanousi A., Elaldi N. (2016). Biosafety standards for working with Crimean-Congo hemorrhagic fever virus. J. Gen. Virol..

[212] Soliman A., Mohareb E., Salman D., Saad M., Salama S., Fayez C., Hanafi H., Medhat I., Labib E., Rakha M. (2010). Studies on West Nile virus infection in Egypt. J Infect Public Health.

[213] Kamal S.A. (2011). Observations on Rift Valley fever virus and vaccines in Egypt. Virol. J..

[214] Arthur R.R., el-Sharkawy M.S., Cope S.E., Botros B.A., Oun S., Morrill J.C., Shope R.E., Hibbs R.G., Darwish M.A., Imam I.Z. (1993). Recurrence of Rift Valley fever in Egypt. Lancet.

[215] Imam I.Z., Darwish M.A., El-Karamany R. (1979). An epidemic of Rift Valley fever in Egypt. 1. Diagnosis of Rift Valley fever in man. Bull. World Health Organ..

[216] Abdel-Moneim A.S., Afifi M.A., El-Kady M.F. (2012). Isolation and mutation trend analysis of influenza A virus subtype H9N2 in Egypt. Virol. J..

[217] Anis A., AboElkhair M., Ibrahim M. (2018). Characterization of highly pathogenic avian influenza H5N8 virus from Egyptian domestic waterfowl in 2017. Avian Pathol..

[218] Elgendy E.M., Watanabe Y., Daidoji T., Arai Y., Ikuta K., Ibrahim M.S., Nakaya T. (2016). Genetic characterization of highly pathogenic avian influenza H5N1 viruses isolated from naturally infected pigeons in Egypt. Virus Genes.

[219] El-Zoghby E.F., Arafa A.S., Hassan M.K., Aly M.M., Selim A., Kilany W.H., Selim U., Nasef S., Aggor M.G., Abdelwhab E.M. (2012). Isolation of H9N2 avian influenza virus from bobwhite quail (Colinus virginianus) in Egypt. Arch. Virol..

[220] Gomaa M.R., Kandeil A., Kayed A.S., Elabd M.A., Zaki S.A., Abu Zeid D., El Rifay A.S., Mousa A.A., Farag M.M., McKenzie P.P. (2016). Serological Evidence of Human Infection with Avian Influenza A H7virus in Egyptian Poultry Growers. PLoS ONE.

[221] Selim A.A., Erfan A.M., Hagag N., Zanaty A., Samir A.H., Samy M., Abdelhalim A., Arafa A.A., Soliman M.A., Shaheen M. (2017). Highly Pathogenic Avian Influenza Virus (H5N8) Clade 2.3.4.4 Infection in Migratory Birds, Egypt. Emerg. Infect. Dis..

[222] Kammon A., Heidari A., Dayhum A., Eldaghayes I., Sharif M., Monne I., Cattoli G., Asheg A., Farhat M., Kraim E. (2015). Characterization of Avian Influenza and Newcastle Disease Viruses from Poultry in Libya. Avian Dis..

[223] Lahlou Amine I., Bajjou T., El Rhaffouli H., Laraqui A., Hilali F., Menouar K., Ennibi K., Boudlal M., Bouaiti E.A., Sbai K. (2011). Pandemic influenza A(H1N1)2009 in Morocco: Experience of the Mohammed V Military Teaching Hospital, Rabat, 12 June to 24 December 2009. Euro. Surveill.

[224] Boukharta M., Azlmat S., Elharrak M., Ennaji M.M. (2015). Multiple alignment comparison of the non-structural genes of three strains of equine influenza viruses (H3N8) isolated in Morocco. BMC Res. Notes.

[225] El Houadfi M., Fellahi S., Nassik S., Guerin J.L., Ducatez M.F. (2016). First outbreaks and phylogenetic analyses of avian influenza H9N2 viruses isolated from poultry flocks in Morocco. Virol. J..

[226] Gould L.H., Osman M.S., Farnon E.C., Griffith K.S., Godsey M.S., Karch S., Mulenda B., El Kholy A., Grandesso F., de Radigues X. (2008). An outbreak of yellow fever with concurrent chikungunya virus transmission in South Kordofan, Sudan, 2005. Trans. R. Soc. Trop Med. Hyg..

[227] Malik A., Earhart K., Mohareb E., Saad M., Saeed M., Ageep A., Soliman A. (2011). Dengue hemorrhagic fever outbreak in children in Port Sudan. J. Infect. Public Health.

[228] Himatt S., Osman K.E., Okoued S.I., Seidahmed O.E., Beatty M.E., Soghaier M.A., Elmusharaf K. (2015). Sero-prevalence of dengue infections in the Kassala state in the eastern part of the Sudan in 2011. J. Infect. Public Health.

[229] Soghaier M.A., Hagar A., Abbas M.A., Elmangory M.M., Eltahir K.M., Sall A.A. (2013). Yellow Fever outbreak in Darfur, Sudan in October 2012; the initial outbreak investigation report. J. Infect. Public Health.

[230] El Moussi A., Ben Hadj Kacem M.A., Ledesma J., Pozo F., Teresa Cuevas M., Casas I., Slim A. (2013). Genetic diversity of influenza B virus in 2009–2010 and 2010–2011 in Tunisia. Med. Mal. Infect..

[231] Wasfi F., Dowall S., Ghabbari T., Bosworth A., Chakroun M., Varghese A., Tiouiri H., Ben Jemaa M., Znazen A., Hewson R. (2016). Sero-epidemiological survey of Crimean-Congo hemorrhagic fever virus in Tunisia. Parasite.

[232] Jori F., Alexander K.A., Mokopasetso M., Munstermann S., Moagabo K., Paweska J.T. (2015). Serological Evidence of Rift Valley Fever Virus Circulation in Domestic Cattle and African Buffalo in Northern Botswana (2010–2011). Front Vet. Sci..

[233] Coetzer A., Coertse J., Makalo M.J., Molomo M., Markotter W., Nel L.H. (2017). Epidemiology of Rabies in Lesotho: The Importance of Routine Surveillance and Virus Characterization. Trop Med. Infect Dis..

[234] Bellan S.E., Cizauskas C.A., Miyen J., Ebersohn K., Kusters M., Prager K.C., Van Vuuren M., Sabeta C., Getz W.M. (2012). Black-backed jackal exposure to rabies virus, canine distemper virus, and Bacillus anthracis in Etosha National Park, Namibia. J. Wildl. Dis..

[235] Umberto M., Aikukutu G., Roux J.P., Kemper J., Ntahonshikira C., Marruchella G., Khaiseb S., Cattoli G., Dundon W.G. (2019). Avian Influenza H5N8 Outbreak in African Penguins (Spheniscus demersus), Namibia, 2019. J. Wildl. Dis..

[236] Monaco F., Pinoni C., Cosseddu G.M., Khaiseb S., Calistri P., Molini U., Bishi A., Conte A., Scacchia M., Lelli R. (2013). Rift Valley fever in Namibia, 2010. Emerg. Infect. Dis..

[237] Thalwitzer S., Wachter B., Robert N., Wibbelt G., Muller T., Lonzer J., Meli M.L., Bay G., Hofer H., Lutz H. (2010). Seroprevalences to viral pathogens in free-ranging and captive cheetahs (Acinonyx jubatus) on Namibian Farmland. Clin. Vaccine Immunol..

[238] Becker W.B. (1966). The isolation and classification of Tern virus: Influenza A-Tern South Africa–1961. J. Hyg. (Lond.).

[239] Thompson P.N., Sinclair M., Ganzevoort B. (2008). Risk factors for seropositivity to H5 avian influenza virus in ostrich farms in the Western Cape Province, South Africa. Prev. Vet. Med..

[240] van Helden L.S., Sinclair M., Koen P., Grewar J.D. (2016). Description of an outbreak of highly pathogenic avian influenza in domestic ostriches (Struthio camelus) in South Africa in 2011. Prev. Vet. Med..

[241] Abolnik C., Pieterse R., Peyrot B.M., Choma P., Phiri T.P., Ebersohn K., Heerden C.J.V., Vorster A.A., Zel G.V., Geertsma P.J. (2019). The Incursion and Spread of Highly Pathogenic Avian Influenza H5N8 Clade 2.3.4.4 within South Africa. Avian Dis..

[242] Abolnik C., Fehrsen J., Olivier A., van Wyngaardt W., Fosgate G., Ellis C. (2013). Serological investigation of highly pathogenic avian influenza H5N2 in ostriches (Struthio camelus). Avian Pathol..

[243] Abolnik C., Olivier A., Reynolds C., Henry D., Cumming G., Rauff D., Romito M., Petty D., Falch C. (2016). Susceptibility and Status of Avian Influenza in Ostriches. Avian Dis..

[244] Abolnik C., Bisschop S.P., Gerdes G.H., Olivier A.J., Horner R.F. (2007). Phylogenetic analysis of low-pathogenicity avian influenza H6N2 viruses from chicken outbreaks (2001–2005) suggest that they are reassortants of historic ostrich low-pathogenicity avian influenza H9N2 and H6N8 viruses. Avian Dis..

[245] Poen M.J., Fouchier R.A., Webby R.J., Webster R.G., El Zowalaty M.E. (2019). Evidence of the Presence of Low Pathogenic Avian Influenza A Viruses in Wild Waterfowl in 2018 in South Africa. Pathogens.

[246] Abolnik C., Strydom C. (2019). Complete Genome Sequence of a Class I Avian Orthoavulavirus 1 Isolated from Commercial Ostriches. Microbiol. Resour. Announc..

[247] Archer B.N., Timothy G.A., Cohen C., Tempia S., Huma M., Blumberg L., Naidoo D., Cengimbo A., Schoub B.D. (2012). Introduction of 2009 pandemic influenza A virus subtype H1N1 into South Africa: Clinical presentation, epidemiology, and transmissibility of the first 100 cases. J. Infect. Dis..

[248] Cohen A.L., Hellferscee O., Pretorius M., Treurnicht F., Walaza S., Madhi S., Groome M., Dawood H., Variava E., Kahn K. (2014). Epidemiology of influenza virus types and subtypes in South Africa, 2009–2012. Emerg. Infect. Dis..

[249] Drewe J.A., O’Riain M.J., Beamish E., Currie H., Parsons S. (2012). Survey of infections transmissible between baboons and humans, Cape Town, South Africa. Emerg. Infect. Dis..

[250] Dickens C., Kew M.C., Purcell R.H., Kramvis A. (2013). Occult hepatitis B virus infection in chacma baboons, South Africa. Emerg. Infect. Dis..

[251] Mathengtheng L., Burt F.J. (2014). Use of envelope domain III protein for detection and differentiation of flaviviruses in the Free State Province, South Africa. Vector Borne Zoonotic Dis..

[252] Komar N., Langevin S., Hinten S., Nemeth N., Edwards E., Hettler D., Davis B., Bowen R., Bunning M. (2003). Experimental infection of North American birds with the New York 1999 strain of West Nile virus. Emerg Infect. Dis..

[253] Mackenzie J.S., Gubler D.J., Petersen L.R. (2004). Emerging flaviviruses: The spread and resurgence of Japanese encephalitis, West Nile and dengue viruses. Nat. Med..

[254] McLean R.G., Ubico S.R., Bourne D., Komar N. (2002). West Nile virus in livestock and wildlife. Curr. Top. Microbiol. Immunol..

[255] Venter M., Swanepoel R. (2010). West Nile virus lineage 2 as a cause of zoonotic neurological disease in humans and horses in southern Africa. Vector Borne Zoonotic Dis..

[256] Venter M., Pretorius M., Fuller J.A., Botha E., Rakgotho M., Stivaktas V., Weyer C., Romito M., Williams J. (2017). West Nile Virus Lineage 2 in Horses and Other Animals with Neurologic Disease, South Africa, 2008–2015. Emerg. Infect. Dis..

[257] Hollidge B.S., Gonzalez-Scarano F., Soldan S.S. (2010). Arboviral encephalitides: Transmission, emergence, and pathogenesis. J. Neuroimmune Pharmacol..

[258] van Eeden C., Williams J.H., Gerdes T.G., van Wilpe E., Viljoen A., Swanepoel R., Venter M. (2012). Shuni virus as cause of neurologic disease in horses. Emerg Infect. Dis..

[259] Malherbe H., Strickland-Cholmley M., Jackson A.L. (1963). Sindbis virus infection in man. Report of a case with recovery of virus from skin lesions. S. Afr. Med. J..

[260] Storm N., Weyer J., Markotter W., Kemp A., Leman P.A., Dermaux-Msimang V., Nel L.H., Paweska J.T. (2014). Human cases of Sindbis fever in South Africa, 2006–2010. Epidemiol. Infect..

[261] Weyer J., Thomas J., Leman P.A., Grobbelaar A.A., Kemp A., Paweska J.T. (2013). Human cases of Wesselsbron disease, South Africa 2010–2011. Vector Borne Zoonotic Dis..

[262] Weiss K.E., Haig D.A., Alexander R.A. (1956). Wesselsbron virus—A virus not previously described associated with abortion in domestic animals. Onderstepoort J. Vet. Res..

[263] van Niekerk S., Human S., Williams J., van Wilpe E., Pretorius M., Swanepoel R., Venter M. (2015). Sindbis and Middelburg Old World Alphaviruses Associated with Neurologic Disease in Horses, South Africa. Emerg. Infect. Dis..

[264] Calisher C.H., Karabatsos N. (1988). Arbovirus Serogroups: Definition and Geographic Distribution.

[265] Vawda S., Goedhals D., Bester P.A., Burt F. (2018). Seroepidemiologic Survey of Crimean-Congo Hemorrhagic Fever Virus in Selected Risk Groups, South Africa. Emerg. Infect. Dis..

[266] Paweska J.T., Sewlall N.H., Ksiazek T.G., Blumberg L.H., Hale M.J., Lipkin W.I., Weyer J., Nichol S.T., Rollin P.E., McMullan L.K. (2009). Nosocomial outbreak of novel arenavirus infection, southern Africa. Emerg. Infect. Dis..

[267] Briese T., Paweska J.T., McMullan L.K., Hutchison S.K., Street C., Palacios G., Khristova M.L., Weyer J., Swanepoel R., Egholm M. (2009). Genetic detection and characterization of Lujo virus, a new hemorrhagic fever-associated arenavirus from southern Africa. PLoS Pathog..

[268] Paweska J.T., Jansen van Vuren P., Kemp A., Storm N., Grobbelaar A.A., Wiley M.R., Palacios G., Markotter W. (2018). Marburg Virus Infection in Egyptian Rousette Bats, South Africa, 2013–2014(1). Emerg. Infect. Dis..

[269] Geldenhuys M., Weyer J., Nel L.H., Markotter W. (2013). Coronaviruses in South African bats. Vector Borne Zoonotic Dis..

[270] von Teichman B.F., de Koker W.C., Bosch S.J., Bishop G.C., Meredith C.D., Bingham J. (1998). Mokola virus infection: Description of recent South African cases and a review of the virus epidemiology. J. S. Afr. Vet. Assoc..

[271] Shope R.E., Murphy F.A., Harrison A.K., Causey O.R., Kemp G.E., Simpson D.I., Moore D.L. (1970). Two African viruses serologically and morphologically related to rabies virus. J. Virol..

[272] Kemp G.E., Causey O.R., Moore D.L., Odelola A., Fabiyi A. (1972). Mokola virus. Further studies on IbAn 27377, a new rabies-related etiologic agent of zoonosis in nigeria. Am. J. Trop Med. Hyg..

[273] Mkhize G.C., Ngoepe E.C., Du Plessis B.J., Reininghaus B., Sabeta C.T. (2010). Re-emergence of dog rabies in Mpumalanga province, South Africa. Vector. Borne Zoonotic Dis..

[274] Bishop G.C., Durrhein D.N., Kloeck P.E., Godlonton J.D., Bingham J., Speare R., Blumberg L., Weyer J., Pienaar H., Markotter W. (2010). Rabies-Guide for the Medical, Veterinary and Allied Professions.

[275] Grover M., Bessell P.R., Conan A., Polak P., Sabeta C.T., Reininghaus B., Knobel D.L. (2018). Spatiotemporal epidemiology of rabies at an interface between domestic dogs and wildlife in South Africa. Sci. Rep..

[276] Gummow B. (2003). A survey of zoonotic diseases contracted by South African veterinarians. J. S Afr Vet. Assoc.

[277] Mundel B., Gear J. (1951). Rift Valley fever; I. The occurrence of human cases in Johannesburg. S. Afr. Med. J..

[278] Gear J., De Meillon B., Le Roux A.F., Kofsky R., Innes R.R., Steyn J.J., Oliff W.D., Schulz K.H. (1955). Rift Valley fever in South Africa; a study of the 1953 outbreak in the Orange Free State, with special reference to the vectors and possible reservoir hosts. S. Afr. Med. J..

[279] Jansen van Vuren P., Kgaladi J., Patharoo V., Ohaebosim P., Msimang V., Nyokong B., Paweska J.T. (2018). Human Cases of Rift Valley Fever in South Africa, 2018. Vector Borne Zoonotic Dis..

[280] Archer B.N., Thomas J., Weyer J., Cengimbo A., Landoh D.E., Jacobs C., Ntuli S., Modise M., Mathonsi M., Mashishi M.S. (2013). Epidemiologic Investigations into Outbreaks of Rift Valley Fever in Humans, South Africa, 2008–2011. Emerg. Infect. Dis..

[281] Fasina F.O., Mokoele J.M., Spencer B.T., Van Leengoed L.A., Bevis Y., Booysen I. (2015). Spatio-temporal patterns and movement analysis of pigs from smallholder farms and implications for African swine fever spread, Limpopo province, South Africa. Onderstepoort J. Vet. Res..

[282] van den Bergh C., Venter E.H., Swanepoel R., Thompson P.N. (2019). High seroconversion rate to Rift Valley fever virus in cattle and goats in far northern KwaZulu-Natal, South Africa, in the absence of reported outbreaks. PLoS Negl. Trop Dis..

[283] Drew T.W., Grierson S.S., King D.P., Hicks D., Done S., Neser J.A., Evans D.P., Grimbeek P., Banks M. (2004). Genetic similarity between porcine circovirus type 2 isolated from the first reported case of PMWS in South Africa and North American isolates. Vet. Rec..

[284] Afolabi K.O., Iweriebor B.C., Obi L.C., Okoh A.I. (2017). Molecular detection of Porcine circovirus type 2 in swine herds of Eastern Cape Province South Africa. BMC Microbiol..

[285] Afolabi K.O., Iweriebor B.C., Obi L.C., Okoh A.I. (2019). Prevalence of porcine parvoviruses in some South African swine herds with background of porcine circovirus type 2 infection. Acta Trop.

[286] Pini A. (1975). Porcine parvovirus in pig herds in Southern Africa. J. S. Afr. Vet. Assoc..

[287] Korsman S., Hardie D., Kaba M. (2019). Hepatitis E virus in patients with acute hepatitis in Cape Town, South Africa, 2011. S. Afr. Med. J..

[288] Nyaga M.M., Peenze I., Potgieter C.A., Seheri L.M., Page N.A., Yinda C.K., Steele A.D., Matthijnssens J., Mphahlele M.J. (2016). Complete genome analyses of the first porcine rotavirus group H identified from a South African pig does not provide evidence for recent interspecies transmission events. Infect. Genet. Evol.

[289] Prozesky L., Theodoridis A. (1977). Diarrhoea in pigs induced by rotavirus. Onderstepoort J. Vet. Res..

[290] Pfitzer S., Verwoerd D.J., Gerdes G.H., Labuschagne A.E., Erasmus A., Manvell R.J., Grund C. (2000). Newcastle disease and avian influenza A virus in wild waterfowl in South Africa. Avian Dis..

[291] Scagliarini A., Piovesana S., Turrini F., Savini F., Sithole F., McCrindle C.M. (2012). Orf in South Africa: Endemic but neglected. Onderstepoort J. Vet. Res..

[292] Famoroti T., Sibanda W., Ndung’u T. (2018). Prevalence and seasonality of common viral respiratory pathogens, including Cytomegalovirus in children, between 0–5 years of age in KwaZulu-Natal, an HIV endemic province in South Africa. BMC Pediatr..

[293] Moyes J., Walaza S., Pretorius M., Groome M., von Gottberg A., Wolter N., Haffejee S., Variava E., Cohen A.L., Tempia S. (2017). Respiratory syncytial virus in adults with severe acute respiratory illness in a high HIV prevalence setting. J. Infect..

[294] Spengane Z., Korsman S., Mkentane K., Davids L.M., Zemanay W., Africa M., Mbhele S., Nicol M., Gumedze F., Ngwanya D. (2018). Blood and virus detection on barber clippers. S. Afr. Med. J..

[295] Meredith C.D., Prossouw A.P., Koch H. (1971). An unusual case of human rabies thought to be of chiropteran origin. S Afr Med. J..

[296] Justman J., Reed J.B., Bicego G., Donnell D., Li K., Bock N., Koler A., Philip N.M., Mlambo C.K., Parekh B.S. (2017). Swaziland HIV Incidence Measurement Survey (SHIMS): A prospective national cohort study. Lancet HIV.

[297] Potter C.W. (2001). A history of influenza. J. Appl Microbiol.

[298] Lowen A.C., Steel J. (2014). Roles of humidity and temperature in shaping influenza seasonality. J. Virol..

[299] Bloom D.E., Black S., Rappuoli R. (2017). Emerging infectious diseases: A proactive approach. Proc. Natl. Acad. Sci. USA.

[300] Bartsch S.M., Gorham K., Lee B.Y. (2015). The cost of an Ebola case. Pathog Glob. Health.

[301] Dimitri N. (2015). The Economics of Epidemic Diseases. PLoS ONE.

[302] Linthicum K.J., Anyamba A., Tucker C.J., Kelley P.W., Myers M.F., Peters C.J. (1999). Climate and satellite indicators to forecast Rift Valley fever epidemics in Kenya. Science.

[303] Zell R. (2004). Global climate change and the emergence/re-emergence of infectious diseases. Int. J. Med. Microbiol..

[304] de Vries R.D., Ludlow M., Verburgh R.J., van Amerongen G., Yuksel S., Nguyen D.T., McQuaid S., Osterhaus A.D., Duprex W.P., de Swart R.L. (2014). Measles vaccination of nonhuman primates provides partial protection against infection with canine distemper virus. J. Virol..

[305] Otsuki N., Nakatsu Y., Kubota T., Sekizuka T., Seki F., Sakai K., Kuroda M., Yamaguchi R., Takeda M. (2013). The V protein of canine distemper virus is required for virus replication in human epithelial cells. PLoS ONE.

[306] Rendon-Marin S., da Fontoura Budaszewski R., Canal C.W., Ruiz-Saenz J. (2019). Tropism and molecular pathogenesis of canine distemper virus. Virol. J..

[307] Lebov J., Grieger K., Womack D., Zaccaro D., Whitehead N., Kowalcyk B., MacDonald P.D.M. (2017). A framework for One Health research. One Health.

[308] Mackenzie J.S., Jeggo M. (2019). The One Health Approach-Why Is It So Important?. Trop Med. Infect. Dis..

[309] El Zowalaty M.E., Järhult J.D. (2020). From SARS to COVID-19: A previously unknown SARS-CoV-2 virus of pandemic potential infecting humans–Call for a One Health approach. One Health.

[310] Andersen K.G., Rambaut A., Lipkin W.I., Holmes E.C., Garry R.F. (2020). The proximal origin of SARS-CoV-2. Nat. Med..

[311] (2020). Coronavirus COVID-19 Global Cases by Johns Hopkins, The Center for Systems Science and Engineering (CSSE). https://gisanddata.maps.arcgis.com/apps/opsdashboard/index.html#/bda7594740fd40299423467b48e9ecf6.

[312] World Health Organization (2019). Novel Coronavirus (2019-nCoV) Situation Report-51.

[313] Morse S.S. (2007). Global infectious disease surveillance and health intelligence. Health Aff. (Millwood).

