# Supplementary material for: Systematic Review of Important Viral Diseases in Africa in Light of the ‘One Health’ Concept
Source: Pathogens. 2020 Apr 20;9(4):301. doi: 10.3390/pathogens9040301 (PMC7238228; doi:10.3390/pathogens9040301)
Supplement: Supplementary file 1 [file pathogens-09-00301-s001.pdf]

Table S1: Zoonotic viruses reported from Africa in selected publications until September 2019.

No.	Country	Virus	Host(s)	References
1.	Algeria	H9N2	Poultry	[193]
		CCHFV	Tick	[194]
		H3N8	Horse	[195]
2.	Angola	Dengue virus	Human	[148]
3.	Benin	IDV	Cattle	[49]
4.	Botswana	RVFV	African buffalo, cattle	[232]
5.	Burkina Faso	H3N2, A(H1N1)pdm09, IBV	Human	[93]
		H9N2	Poultry	[94]
		RVFV	Cattle, goat, sheep	[92]
6.	Burundi	HCV	Human	[149]
7.	Cameroon	Adenovirus, enterovirus, HIV-1, STLV-1, 2	Non-human primates	[154, 165]
		H3N2, IBV, CCHFV, H1V, HIV-1, HTLV-1, 2, 3, 4, MPXV, RVFV, SFV	Human	[1, 52, 97, 153, 157-164]
		H5N1	Poultry	[167]
		H5N8	Pigeon, chicken, guinea fowl, duck	[152]
		HEV, H1N1, H3N2, A(H1N1)pdm09, Porcine hokovirus	Pig	[32, 129, 151, 156, 166]
8.	Cape Verde	H3N2, A(H1N1)pdm09, IBV	Human	[95]
9.	Central African Republic	MPXV	Human	[168]
10.	Chad	RVFV	Cattle, goat, sheep	[169]
11.	Comoros	DUVV, LBV	Bat	[41]
12.	Cote d' Ivoire	Infectious bronchitis virus, NDV	Chicken, duck, guinea fowl	[100]
		H3N2, A(H1N1)pdm09, IBV, Ebola virus, HIV-1, 2, MPXV	Human	[52, 99]
		IAV	Bird, pig	[91]
		SFV	Chimpanzee	[98]
13.	Democratic Republic of Congo	Ebola virus, HIV, MPXV, SFV, Varicella zoster virus, Orthopox virus	Human	[71, 97, 109, 157, 171]
		Adenovirus	Chimpanzee, gorilla	[150]
		H5N8	Duck	[170]
		MPXV	Wild animals	[171]
		RVFV	Cattle	[179]
14.	Djibouti	H3N2, A(H1N1)pdm09	Human	[42]
15.	Egypt	H5N1, H7N7, RVFV, WNV	Human	[196, 199-200, 205, 212, 220]
		H5N1	Pigeon	[218]
		H5N8	Duck, migratory bird	[217, 221]
		H9N2	Poultry, Bobwhite quail	[216, 219]
		IAV, H5N1, H9N2, A(H1N1)pdm09	Pig	[34]
		CCHFV, RVFV	Cow	[202-204, 210]
		MERS-CoV, RVFV	Camel	[207-209]
		RVFV	Cattle, buffalo, sheep, goat, horse, rat	[203-204, 210]
		Rabies virus	Dog, cat, jackal	[10, 202]
16.	Equatorial Guinea	HBV, HCV, HIV	Human	[180]
17.	Eritrea	Dengue virus	Human	[43]
18.	Ethiopia	Calicivirus	Pig	[48]

		MERS-CoV	Camel	[47]
		Rabies virus	Dog, human, cattle, horse, goat	[44-46]
19.	Gabon	Ebola virus, MPXV,SFV	Human	[182-184]
		HIV, SFV, HTLV	Non-human primates	[2-3, 71, 184]
		Chikungunya virus	Mosquito	[181]
20.	Gambia	HIV, HBV	Human	[101]
21.	Ghana	Achimota virus, A(H1N1)pdm09, Dengue virus, Zika virus, Lassa virus, Rabies virus	Human	[67, 103, 105-107]
		H3N2, A(H1N1)pdm09	Pig	[31, 103]
		H5N1, H9N2, IBV	Poultry	[14, 104]
		Nipah virus, Rubula virus	Bat	[60, 67]
22.	Guinea	Ebola virus, H3N2, IBV, A(H1N1)pdm09, Lassa virus	Human	[109-115]
		Lassa virus	Rodents	[116]
23.	Guinea-Bissau	HTLV-1	Human	[117]
24.	Kenya	H1N1, H3N2, A(H1N1)pdm09, IBV, RVFV	Human	[50, 52]
		H1N1, H3N2, A(H1N1)pdm09, Porcine bocavirus, Porcine circovirus, Porcine rotavirus, Kobuvirus, Mamastrovirus, Sapelovirus, Swine pasivirus-1, Porcine teschovirus, Picobirnavirus, Posavirus	Pig	[29, 51]
		ICV, IDV, MERS-CoV	Camel	[49, 54]
		H4N6	Water bird	[19]
		AOaV-1	Poultry	[53]
		IAV	Chicken, dog, duck	[29]
		RVFV	Cattle	[50]
25.	La Reunion	DUVV, LBV, EBLV-1	Bat	[41]
26.	Lesotho	Rabies virus	Dog, cat, cattle, goat, horse, sheep, pig	[233]
27.	Liberia	Ebola virus, MPXV	Human	[97, 110, 112, 118, 119]
28.	Libya	H5N1, H9N2, APMV-1	Poultry	[222]
29.	Madagascar	H1N1, H3N2, A(H1N1)pdm09, IBV, CCHFV	Human	[7, 52]
		DUVV, LBV, EBLV-1	Bat	[41]
		Anjozorobe virus	Rat	[55]
		Bluetongue virus	Mosquito	[57]
		HEV	Pig	[59]
		RVFV	Cattle	[58]
30.	Malawi	ASFV	Pig, tick	[62]
		Nipah virus	Bat	[60]
31.	Mali	A(H1N1)pdm09	Human	[122]
		Lassa virus	Rodents	[120]
32.	Mauritania	H1N1, H3N2, A(H1N1)pdm09, IBV, RVFV, Dengue virus, CCHFV, WNV	Human	[95, 123]
33.	Mauritius	DUVV, LBV, EBLV-1	Bat	[41]
34.	Morocco	A(H1N1)pdm09	Human	[223]
		H9N2	Poultry	[225]
		H3N8	Horse, donkey, mule	[224]
		IDV	Cattle	[49]
35.	Mozambique	H3N2, A(H1N1)pdm09, Chikungunya virus, Dengue virus, IBV, RVFV, WNV	Human	[63-64]

36.	Namibia	H5N8	Penguin	[235]
		CDV, Rabies virus	Jackal, kudu, dog	[234]
		CDV, Feline herpesvirus, Feline calicivirus, Feline parvovirus, Feline coronavirus, Rabies virus, Puma lentivirus	Cheetah, Caracal	[237]
		RVFV	Sheep, goat	[236]
37.	Niger	RVFV	Ruminants	[124]
38.	Nigeria	H1N1, H3N2, H5N1, H5N2, H9N2, H11N2, Chikungunya virus, Dengue virus, YFV, WNV, Zika virus	Human	[4, 35, 125, 103, 128, 131-134]
		H1N1, H3N2, A(H1N1)pdm09, H5N1, HEV	Pig	[23, 30, 31, 102, 103,135]
		Ebola virus	Non-human primates	[136]
		H3N8	Dog	[130]
		MERS-CoV	Camel	[47]
		NDV	Bird, duck	[137]
39.	Republic of Congo	Ebola virus, MPXV, Zika virus	Human	[71, 97, 188]
40.	Rwanda	H1N1, H3N2, A(H1N1)pdm09	Human	[190]
		RVFV	Cattle	[189]
41.	Sao Tome and Principe	Dengue virus	Human	[191]
42.	Senegal	H1N1, H3N2, A(H1N1)pdm09, Chikungunya virus, IBV, RVFV	Human	[95, 142, 143]
		WNV	Horse, wild bird	[140, 141]
43.	Seychelles	DUVV, LBV, EBLV-1	Bat	[41]
44.	Sierra Leone	Ebola virus, MPXV	Human	[96, 97, 110, 144-147]
45.	Somalia	HBV, HCV, HIV-1	Human	[65, 66]
46.	South Africa	Adenovirus, H5N2, H7N1, IAV, H3N2, A(H1N1)pdm09, IBV, CCHFV, Cytomegalovirus, DUVV, HIV, HBV, HEV, Kunjin virus, Human rhinovirus, Langat virus, Lujo virus, Orf virus, Rabies virus, RSV, RVFV, Sindbis virus, Wesselsbron virus, WNV	Human	[39, 52, 246, 247, 250, 259, 260, 264-266, 277, 279, 291-294]
		ASFV, PCV-2, PPV-1, 2, 3, 4, PBo-likeV, PBoV-1, 2, Rotavirus	Pig	[280, 282-285, 287,288]
		AHSV, Equine encephalitis virus, Kunjin virus, Middelburg virus, Shuni virus, Sindbis virus, WNV	Horse	[254-257, 262]
		H5N2, H7N1, H1N2, H6N2, H9N2, H6N1, H7N7, H6N8, H10N1, AOaV-1,	Ostrich	[5, 13, 239, 240, 242-245]
		Avian influenza (Tern virus)	Tern	[238]
		H5N8, H6N2	Poultry	[241]
		Coronavirus, DUVV, Marburg virus	Bat	[267, 268]
		H5N2, H4N2, H1N8	Egyptian geese	[243]
		H3N8	Cape shoveller	[243]
		H4N8, H11N2	Red-billed teal	[243]
		H5N1	Yellow-billed duck	[243]
		H10N7	Pekin duck	[243]
		H7N8	Shell duck	[243]
		Influenza A virus (H?N?)	Egyptian geese	[245]
		H10N9, NDV	Aquatic wild bird	[290]

		Cytomegalovirus, Epstein-Barr virus, HAV, HBV	Baboon, Chacma baboon	[249, 250]
		DMVV-1	Vervet monkey	[81]
		Kunjin virus, Langat virus, Orf virus, RVFV, Wesselsbron virus, WNV	Cattle	[251, 282, 291]
		Kunjin virus, Langat virus, Orf virus, Wesselsbron virus, RVFV, WNV	Sheep	[251, 278, 279, 291]
		Orf virus, RVFV	Goat	[282,291]
		Mokola virus	Cat	[270]
		Rabies virus	Dog, spotted genet, baboon	[274, 275]
47.	South Sudan	MPXV, Varicella-zoster virus	Human	[192]
48.	Sudan	Chikungunya virus, Dengue virus, Ebola virus, YFV	Human	[71, 226-229]
49.	Swaziland	HIV	Human	[296]
50.	Tanzania	Achimota virus, Nipah virus	Bat	[60, 67]
		CDV	Lion	[69]
		Rabies virus	Dog	[70]
51.	Togo	A(H1N1)pdm09	Pig	[33]
		IDV	Cattle, sheep, goat	[49]
52.	Tunisia	IAV, IBV, CCHFV	Human	[230, 231]
		MERS-CoV	Camel	[47]
53.	Uganda	Ebola virus, Ntwetwe virus, A(H1N1)pdm09, WNV	Human	[74-77]
		Porcine bocavirus, Porcine circovirus, Porcine rotavirus, Kobuvirus, Mamastrovirus, Sapelovirus, Swine pasivirus-1, Porcine teschovirus, Picobirnavirus, Posavirus	Pig	[51]
		H5N8	Bird	[78]
		Human Rhinovirus C	Chimpanzee	[73]
		Nipah virus	Bat	[60]
		RVFV	Cattle, goat, sheep	[9]
54.	Zambia	ASFV	Pig	[79, 80]
		H3N6, H9N1	White pelican	[87]
		H3N8, H6N2, H11N9	Wild duck	[87]
		H3N8, H4N6, H11N9	Wild goose	[87]
		Canine parvovirus	Dog	[84]
		Rabies virus	Cat, cow, dog, human, jackal, pig	[85]
		Nipah virus	Bat	[60]
		Simian pegivirus	Malbroucks monkey	[81]
		WNV	Mosquito	[82]
		Zika virus	Non-human primates	[88]
55.	Zimbabwe	Avian influenza virus, APMV-1, WNV	Bird	[89]
		Rabies virus	Bat, bushbaby, cat, cattle, civet, cow, dog, duiker, kudu, genet, goat, horse, jackal, lion, pig, monkey, rabbit, rodents, sable antelope, sheep, wildebeest, zebra	[85, 90]

Abbreviations: AHSV= African Horse Sickness Virus, APMV-1= Avian Paramyxovirus type-1, AOaV-1= Avian Orthoavulavirus-1, ASFV= African Swine Fever Virus, CCHFV= Crimean-Congo Haemorrhagic Fever Virus, DUVV= Duvenhage lyssavirus, LBV= Lagos bat lyssavirus, EBLV-1=

European bat lyssavirus-1, HTLV= Human T-Lymphotropic Virus, IAV= Influenza A virus, IBV= Influenza B virus, ICV= Influenza C virus, IDV= Influenza D virus, CDV= Canine Distemper Virus, HAV= Hepatitis A virus, HBV= Hepatitis B virus, HCV= Hepatitis C virus, HEV= Hepatitis E virus, HIV= Human Immunodeficiency Virus, MERS-CoV= Middle East Respiratory Syndrome-Coronavirus, NDV= Newcastle Disease Virus, PCV-2= Porcine Circovirus type-2, PPV= Porcine Parvovirus, PBoV1= Porcine Bocavirus 1, PBo-likeV= Porcine Bocavirus-like virus, RVFV= Rift Valley Fever Virus, SFV= Simian foamy virus, STLV= Simian T-cell leukemia virus, WNV= West Nile Virus, YFV= Yellow Fever Virus.
